# Source Geometry and Receiver Ascertainment Limit Spatial Perturbation Inference

**DOI:** 10.34133/csbj.0258

**Published:** 2026-11-10

**Authors:** Zongnan Lyu, Qi Yu, Renyu Yang, Chunxue Shao, Guang Yang, Ziheng Wang

**Affiliations:** Division of Computational Biology, Chinese Center of Exercise Epidemiology, Northeast Normal University, Changchun, 130024, Jilin, China.

## Abstract

Spatial perturbation assays locate targeted cells, but neighboring-cell responses can also reflect tissue structure, imperfect guide detection, and dependent sampling. We evaluated these alternatives with a spatial perturbation field diagnostic workflow combining independently specified receiver scores, spatial adjustment, and explicit source references. In 3 Spatial Perturb-Seq chips spanning 4 mice, a *Clu*-associated score was not exceptional under the stricter reference; training-only mouse holdouts also failed to establish stable program transfer. Known-effect injections showed that refitting spatial background retained 73.7% of the contrast gain at a 150-μm decay scale and 63.3% at 600 μm. In a separate count-level simulation, 3.82% receiver contamination by missed sources produced 33/100 zero-field rejections with source-autonomous expression changes, versus 3/100 without them. Perturb-map stromal contrasts were positive in 2 animals, but dependent sections, unresolved clone identities, and insufficient matched-placement support prevented population-level confirmation. External assays, injury, and clinical cohorts did not establish broad transportability or incremental prediction. These results distinguish limits in receiver ascertainment, source-reference validity, and biological replication, providing reproducible diagnostics rather than a generally calibrated test of causal propagation.

## Introduction

Genetic and pharmacological perturbations are usually summarized by comparing directly targeted cells with untreated or nontargeting controls. Pooled single-cell CRISPR screens link perturbation identity to transcriptional readouts [1–4], with targeted and genome-scale implementations [5,6]. This cell-autonomous view is powerful in dissociated systems, but it is incomplete in intact tissues. Secreted factors can diffuse or be relayed between cells; membrane-bound ligands can act across physical contacts; extracellular matrix can store, release, or mechanically transmit signals; vascular routes can connect spatially separated niches; and recruited immune cells can amplify an initially local event. A perturbation may therefore alter cells that never received the guide, drug or injury. The resulting response is not simply a property of a target cell, but a spatially organized change in a multicellular system.

Spatial RNA measurements include spatially barcoded arrays [7,8], bead-based capture [9,10], dense spatial barcoding [11–13] and multiplexed imaging [14,15]. Spatial perturbation assays couple molecular readouts to the locations of targeted cells. Spatial Perturb-Seq measures guide identity and expression in intact tissue [16]; Perturb-map combines genetically labeled tumor lesions with spatial profiling [17]; and Perturb-DBiT comeasures RNA and guides in spatial capture spots [18]. A source is a measured cell or spot carrying the focal guide, or a genetically labeled lesion. A receiver is an eligible measurement outside that observed source set. In a guide assay, an undetected guide does not prove that a cell is unperturbed. Table 1 distinguishes these designs before their results are compared.

**Table 1.** Assays, source definitions and the units available for inference. Counts are descriptive coverage, not interchangeable independent replicates.

System and source	Source / receiver measurement	Coverage and inferential unit
Spatial Perturb-Seq [16]	One detected focal guide / guide-undetected cell; cell-resolved Stereo-seq	229,775 cells, 3 chips spanning 4 mice: chip 1, mouse 1; chip 2, mice 2/3; chip 3, mouse 4. Chip 2 pools animals in 1 analysis block.
Perturb-map [17]	Genetically labeled lesion / unlabeled or periphery-annotated Visium spot	6,363 spots, 4 sections from 3 mice; focal comparison uses 4 *Tgfbr2* and 10 KP lesion annotations in 3 sections from 2 mice. Gene Expression Omnibus (GEO)-based grouping: KP-1/KP-2 share mouse 4; KP-4 is mouse 7. KP-3 (mouse 5) is excluded.
Perturb-DBiT [18]	Guide-positive capture spot / guide-undetected spot	16,878 tissue/expression-matched spots in 6 audited sections; 2 sections per external focal test; animal independence not assumed.
Myocardial infarction (MI) [64]	Composition-defined injury proxy / other Visium spots	88,704 spots, 27 sections, 20 patients; patient-level inference.
Skin injury (GSE278169)	High-inflammation proxy / remaining spots	3,885 spots in 5 expression-valid sections; cross-sectional section-level comparisons.
Paired hepatocellular carcinoma (HCC) [51] (Zenodo 19123188)	Apoptosis proxy / remaining Visium spots	104,418 spots, 22 sections, 11 patients; 1 posttreatment sample is nodal metastasis; patient-level validation.
External HCC [40]	Apoptosis proxy / remaining Visium spots	17,292 spots, 7 resection specimens; specimen-level locked validation.

Our question is whether a molecular response in receivers remains associated with source proximity after accounting for receiver identity, endogenous spatial structure, and source geometry. This distinction matters biologically: A niche shared by source and receiver cells does not establish an extracellular target or a therapeutic opportunity to interrupt transmission. Giotto and Squidpy quantify spatial neighborhood structure [19,20]; SpatialDE, trendsceek and SPARK detect spatial expression [21–23]; and CellPhoneDB, CellChat, and NicheNet nominate communication or ligand–target relationships [24–26]. Their endpoints differ from a perturbation-associated receiver response. Spatial reference tests have precedents in multitype point-pattern analysis [27] and spatial-map correspondence testing [28]. We adapt design-aware permutation principles [29] to the receiver-response question.

Graph-based spatial-domain representations describe tissue organization [30]; GRAS4T uses modality-aware and subspace-enhanced graph contrastive learning for domain identification [31]. Reviews of perturbation modeling cover molecular and multicellular response prediction across single-cell modalities [32,33]. These prediction and representation tasks differ from testing a source-conditioned spatial reference.

The central inferential problem is that perturbation-positive cells are rarely placed independently and uniformly in tissue. They form clones, colonies, injection tracks, or injury cores, and those structures preferentially occupy particular anatomical or transcriptional niches. Nearby guide-undetected cells can share that niche even if the perturbation has no noncell-autonomous effect. A permutation that scatters the correct number of pseudo-sources destroys the clustering that generated the confounding and can therefore produce an apparently compelling false-positive field. Changes in cell composition create a second ambiguity: A neighborhood score can increase because resident cells changed state, because a different cell type entered the region, or both. Cell-type deconvolution methods explicitly address the related problem of mixed-cell spatial measurements [34]. Endogenous 2-dimensional gradients, disconnected tissue components, and boundaries further complicate interpretation. Spatial profiling of the human cortex illustrates how strongly expression patterns can track preexisting anatomical layers [35]. Consequently, neither ligand–receptor coexpression nor monotonic decline with Euclidean distance is sufficient evidence for a transmitted causal chain.

We use spatial perturbation field (SPF) to denote a diagnostic workflow and its estimated distance profile (Fig. 1). Let *Y_ip_* be a frozen molecular program score for receiver *i* and perturbation *p*, *Z_i_* the measured background covariates, and $r_{\mathrm{ip}}=Y_{\mathrm{ip}}-{\hat{m}}_{p}\left(Z_{i}\right)$ the adjusted score. For a distance bin *I*, we estimate:

$${\hat{K}}_{\mathrm{sp}}\left(I\right)=\frac{\sum_{i\in R_{s}}1\left\{d\left(i,{\;S}_{\mathrm{sp}}\right)\in I\right\}r_{\mathrm{ip}}}{\sum_{i\in R_{s}}1\left\{d\left(i,{\;S}_{\mathrm{sp}}\right)\in I\right\}}-\frac{1}{B}\sum_{b=1}^{B}\frac{\sum_{i\in R_{s}^{\left(b\right)}}1\left\{d\left(i,{\;S}_{\mathrm{sp}}^{\left(b\right)}\right)\in I\right\}r_{\mathrm{ip}}}{\sum_{i\in R_{s}^{\left(b\right)}}1\left\{d\left(i,{\;S}_{\mathrm{sp}}^{\left(b\right)}\right)\in I\right\}}.$$ (1)

**Fig. 1.**
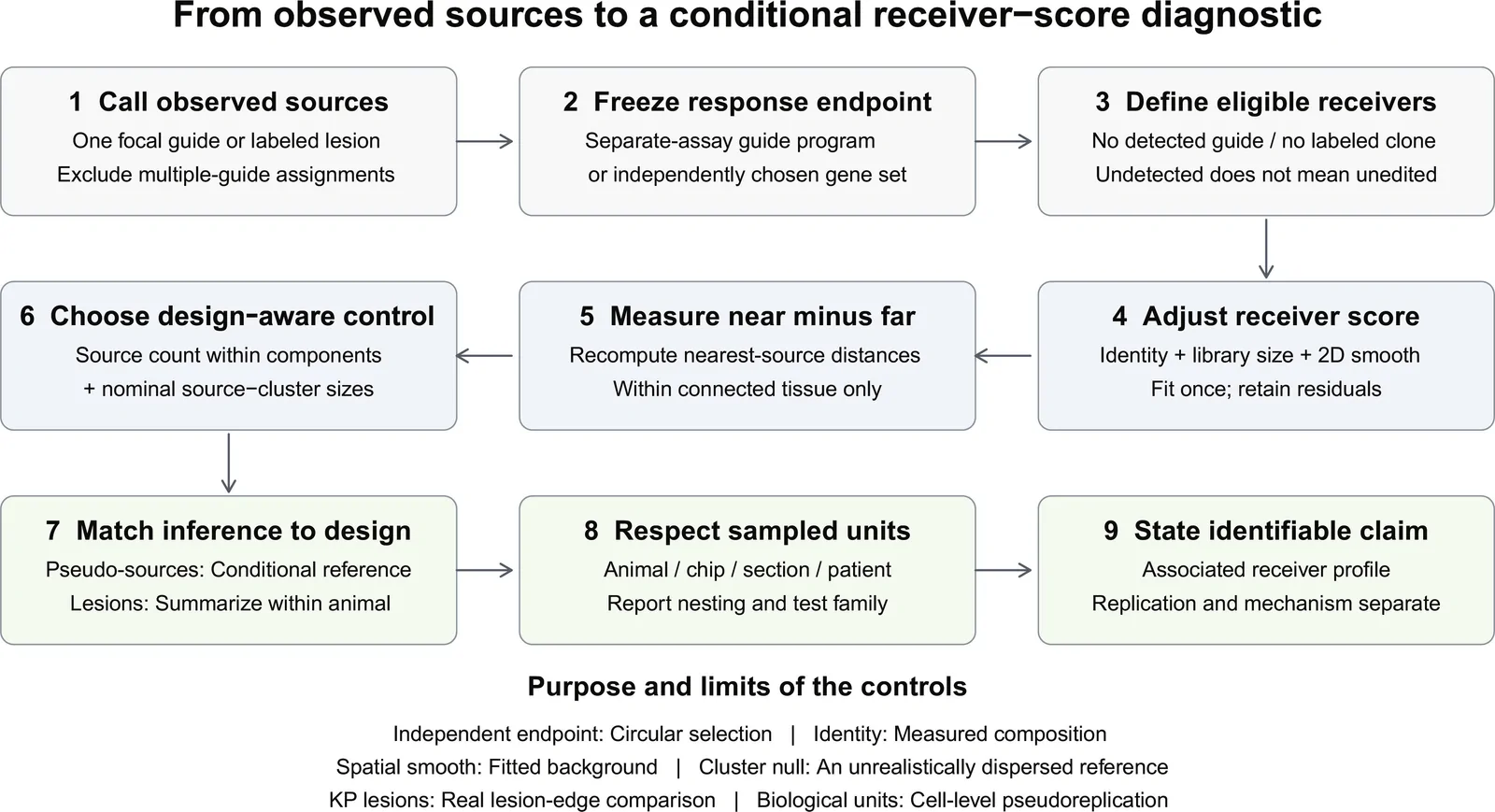
Spatial perturbation field (SPF) workflow and the competing explanations addressed by each control. Source calling defines observed perturbation locations; independent program learning prevents neighborhood-driven feature selection; receiver eligibility excludes detected guides or labeled clones. Receiver identity and spatial adjustment address measured composition and endogenous gradients. Pseudo-sources preserve component-specific source counts and, for the stricter reference, nominal placement-group sizes derived from observed source-connected clusters; this addresses the use of an unrealistically dispersed source reference but does not ensure niche exchangeability. Real KP lesions provide a separate within-section boundary control. Scores and covariates remain fixed across source randomizations, whereas source distances, pseudo-source receiver exclusions, and contrasts are recomputed. Biological-unit inference follows the sampled design. The diagram is schematic and contains no additional biological observations.

Here, *s* is a section or chip, $R_{s}$ its observed eligible receiver set, $R_{s}^{\left(b\right)}=R_{s}\backslash S_{\mathrm{sp}}^{\left(b\right)}$ the receiver set after excluding cells at selected pseudo-source positions, $S_{\mathrm{sp}}$ the observed sources, *d* nearest-source distance within connected tissue in assay-specific units (micrometers where calibrated), and $S_{\mathrm{sp}}^{\left(b\right)}$ a geometry-constrained pseudo-source set. The first term is the observed mean adjusted response; the second is its mean under the specified null. Empty bins are unestimable. The comparison uses a near–far contrast of these scores and the corresponding pseudo-source configurations. Perturb-map instead compares the near–far contrasts of real perturbed and *Kras*^G12D^/*Trp53*-null (KP) control lesions within sections. Thus, the field is a conditional, program-specific association; it is not a diffusion coefficient or an identified causal transport kernel.

Response means a measured molecular-score change. Field means its distance profile. Reorganization includes changes in composition or cellular state. We reserve propagation for a noncell-autonomous process requiring additional causal evidence and use transportability for reproducibility of a response representation in another dataset. Boundary permeability, transport mechanism, and propagation speed are not estimated here.

The contribution is a set of executable diagnostics for receiver ascertainment, source-reference validity, and biological replication. We examine these requirements in Spatial Perturb-Seq and Perturb-map, using known-effect benchmarks, real-coordinate injections, guide-error simulations, and training-only mouse holdouts. The *Clu* example asks how a fixed score changes under source and background controls; the *Tgfbr2* reanalysis describes a control-lesion-relative stromal score profile in 2 animals, extending the spatial description of the phenotype already reported by the source study [17]. *Clu* encodes clusterin, a secreted chaperone; *Tgfbr2* encodes a transforming growth factor-*β* (TGF-*β*) receptor. External assay, injury, and patient analyses are stress tests of scope. The inferential scope of these analyses depends on the source-assignment law, receiver ascertainment, and biological replication.

## Results

### A multiplatform design distinguishes genetic and proxy sources

The analyses were grouped by source definition and sampling design. Their roles and analysis choices were not prospectively registered. Spatial Perturb-Seq and Perturb-map formed the primary genetic examples because they provided observed perturbation sources. Perturb-DBiT was acquired after the primary analyses were frozen and was used only as an external assay stress test. Human myocardial infarction (MI), a mouse skin-wound series and 2 hepatocellular carcinoma (HCC) treatment cohorts tested transfer to observational proxy sources. These injury and clinical samples were not randomized perturbation experiments; their high-score regions were treated as proxy sources, and their results were evaluated separately from the genetic perturbation analyses. Table 2 summarizes the evidence status of the principal spatial-field claims.

**Table 2.** Evidence status of the principal spatial-field claims

System	Primary claim	Decisive control or result	Status
Spatial Perturb-Seq	*Clu* receiver field	Strict chip-wise *P*_ref_ = 0.78,0.91,0.99	Not exceptional under reference
Perturb-map	*Tgfbr2* stromal association	Two animal contrasts: 0.9707 and 1.6820; unresolved cross-sectional clones	Descriptive; unconfirmed
Perturb-DBiT	*ATG4A* held-out field	Cluster reference *P*_ref_ = 0.994	Same-block diagnostic
Perturb-DBiT	*Tgfbr2* target field	Same-block section fractions *P*_ref_ = 0.381, 0.614	Not exceptional under reference
Injury	Conserved persistent wave	Patient/section exact tests all nonsignificant	No conserved field established
HCC	Incremental clinical field embedding	Locked external SPF AUC 0.667; internal receiver-only performance poor	No consistent predictive gain

SPF, spatial perturbation field; AUC, area under the receiver operating characteristic curve

The frozen resource contained 68 spatial assay blocks (3 Stereo-seq chips and 65 other sections) and 450,437 cells or spots across the primary and transport datasets (Table 1); the subsequently acquired Perturb-DBiT sections were additional stress tests. These totals describe coverage, not an aggregate number of independent biological replicates. Spatial Perturb-Seq included 4,755 guide-detected cells: 3,669 single-guide and 1,086 multiple-guide cells. The 45 guide–chip pairs with at least 5 sources are coverage summaries, not a justified power threshold. Multiple-guide cells were excluded from both source assignment and receiver inference. Source number, niche coverage, and independent replication must be assessed separately for every perturbation (Supplementary Methods, SM2, SM3, and SM6).

Single-guide sources and broad receiver identities occupied structured tissue domains (Fig. 2A and B). This spatial organization is the confounding structure tested below; it does not itself imply a receiver effect.

**Fig. 2.**
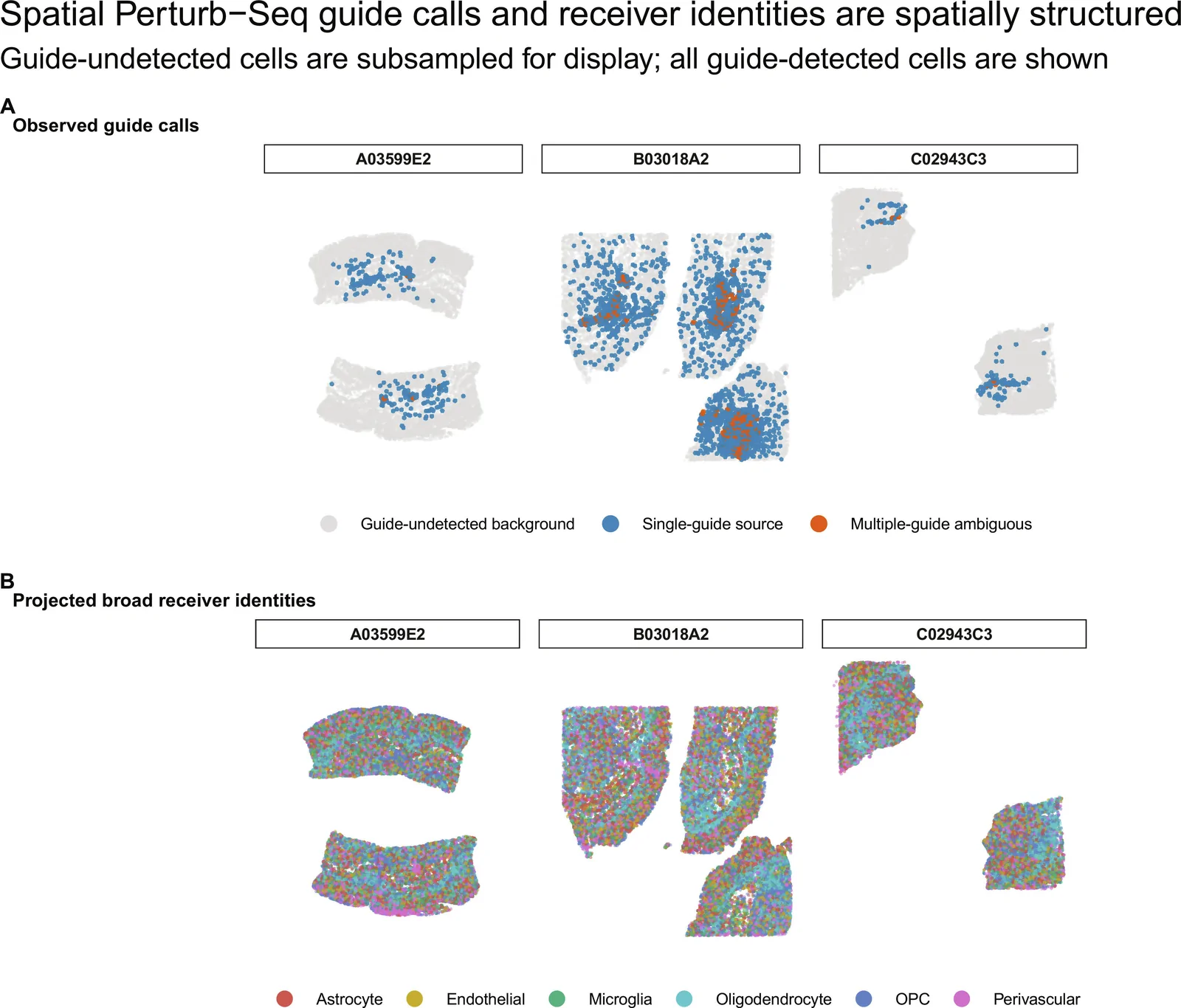
Spatial Perturb-Seq guide calls and receiver identities are spatially structured. (A) Observed guide calls across 3 Stereo-seq chips spanning 4 published mice (229,775 cells). Chip 2 pools mice 2 and 3; their cells cannot be assigned to separate animals from the available metadata. Blue cells carry one detected guide and define eligible sources; orange cells carry multiple guides, remain assignment-ambiguous, and are excluded from downstream source definitions. All guide-detected cells are shown; guide-undetected background cells were subsampled only for visualization. (B) Broad identities projected for guide-undetected receivers in the same tissue coordinates. The joint spatial structure of source placement and receiver identity motivates a null that preserves source clusters and endogenous 2-dimensional background.

### Direct-response programs are learned outside the spatial receiver test

The need to separate feature selection from evaluation is established in high-dimensional expression prediction [36]. Program-level interpretation has precedents in gene set enrichment analysis [37] and in the curated Molecular Signatures Database (MSigDB) and its Hallmark collection [38,39]. Here, learned direct-response weights and predefined gene-set scores were used as receiver endpoints; a program score was not itself treated as evidence for spatial propagation.

A receiver program selected from the same spatial neighborhood in which it is tested can encode local background and inflate the apparent field. Direct-response programs were learned in a matched 5-mouse single-cell Perturb-seq experiment, and their gene weights were frozen before projection into Spatial Perturb-Seq. Single-guide cells were matched without replacement to guide-undetected cells from the same mouse in a continuous transcriptional-state space (Methods, section “Program learning and projection”; Supplementary Methods, SM2 to SM5). Differential expression was estimated by paired mouse-level pseudobulk analysis with mouse and guide-group terms; transcriptional state entered matching, while no explicit identity regression covariate was included. Genes altered by the mSafe control were removed as an empirical negative-control blacklist. This procedure yielded guide-associated direct-response programs for 11 perturbations, defined relative to guide-undetected controls. These controls could themselves have experienced neighboring perturbations; the weights therefore do not isolate a pure cell-autonomous effect. Ten program names matched evaluable spatial guide labels; *Dpp6* was not matched to the differently named *Dpp5* spatial feature.

As an internal check, nonguide genes were analyzed across the 3 spatial chips using chips, rather than cells, as replicates; none passed the stated criteria. The externally learned *Clu* program used 78 source cells across 5 mice and 12 genes, reduced to 9 after immediate-early-gene exclusion. We then rebuilt preprocessing, matching, mSafe calibration, and program learning in each 4-mouse training fold. Query matching used a frozen projection from principal component analysis fitted on the training data because the fitted Harmony workflow supplied no unseen-animal operator (SM13). Primary *Clu* no-immediate-early-gene areas under the receiver operating characteristic curve (AUCs) were 0.531 and 0.359; 3 folds lacked 3 eligible mSafe training mice. All 17 non-mSafe guides, 2 matching routes, and 2 gene variants are reported, including unavailable folds. Neither the exact joint-reference replay nor these transfer results establishes program specificity or stable generalization. The spatial analysis therefore evaluates this fixed score. Its result does not exclude *Clu* receiver responses that the score fails to capture.

### *Clu* score contrasts depend on background and source reference

For each frozen perturbation program, we calculated the distance from every guide-undetected receiver to its nearest compatible source within the same connected tissue component. The initial endpoint contrasted receivers within 100 μm with those 400 to 800 μm away. Pseudo-source configurations preserved source number in each disconnected component. Under this source-count control, the no-immediate-early-gene *Clu* program was positive in all 3 chips. Its mean reference-adjusted near–far effect was 0.092 robust SDs, about one-tenth of the receiver-score dispersion and not a 9.2% expression change. The historical cross-chip normal-score approximation gave a Benjamini–Hochberg (BH)-adjusted reference value of 0.00425 across 10 evaluable programs; the approximation does not provide calibrated false-discovery-rate control for these source placements. It is a screening result across 3 chip blocks spanning 4 mice, not a 4-mouse effect estimate or biological validation of the program. The original declining profile and strict-null profile are supplied together in Fig. S22A and B.

A stricter source reference combined spatial background adjustment with component counts and nominal placement-group sizes derived from observed source-connected clusters. Broad receiver identities were projected from guide-undetected reference cells; program scores were adjusted for receiver identity, library size, and a 2-dimensional smooth of endogenous expression. The resulting groups need not retain the observed connected geometry (Methods and SM5). Under this joint control, all-receiver excesses were 0.0078, −0.0024, and 0.0011 in the 3 chips, with conditional Monte Carlo reference fractions $P_{\mathrm{ref}}=0.78$, 0.91, and 0.99, respectively (Fig. 3D and E, Fig. S24B, and SM14). No identity had a BH-adjusted reference value below 0.05; this descriptive adjustment does not establish false-discovery-rate control. Directional amplitudes were also compatible with their 1-sided clustered-source reference (0.93, 0.13, and 0.70; SM5), and quarter-peak radius estimates varied from 200 to 400 μm without a common preferred axis.

**Fig. 3.**
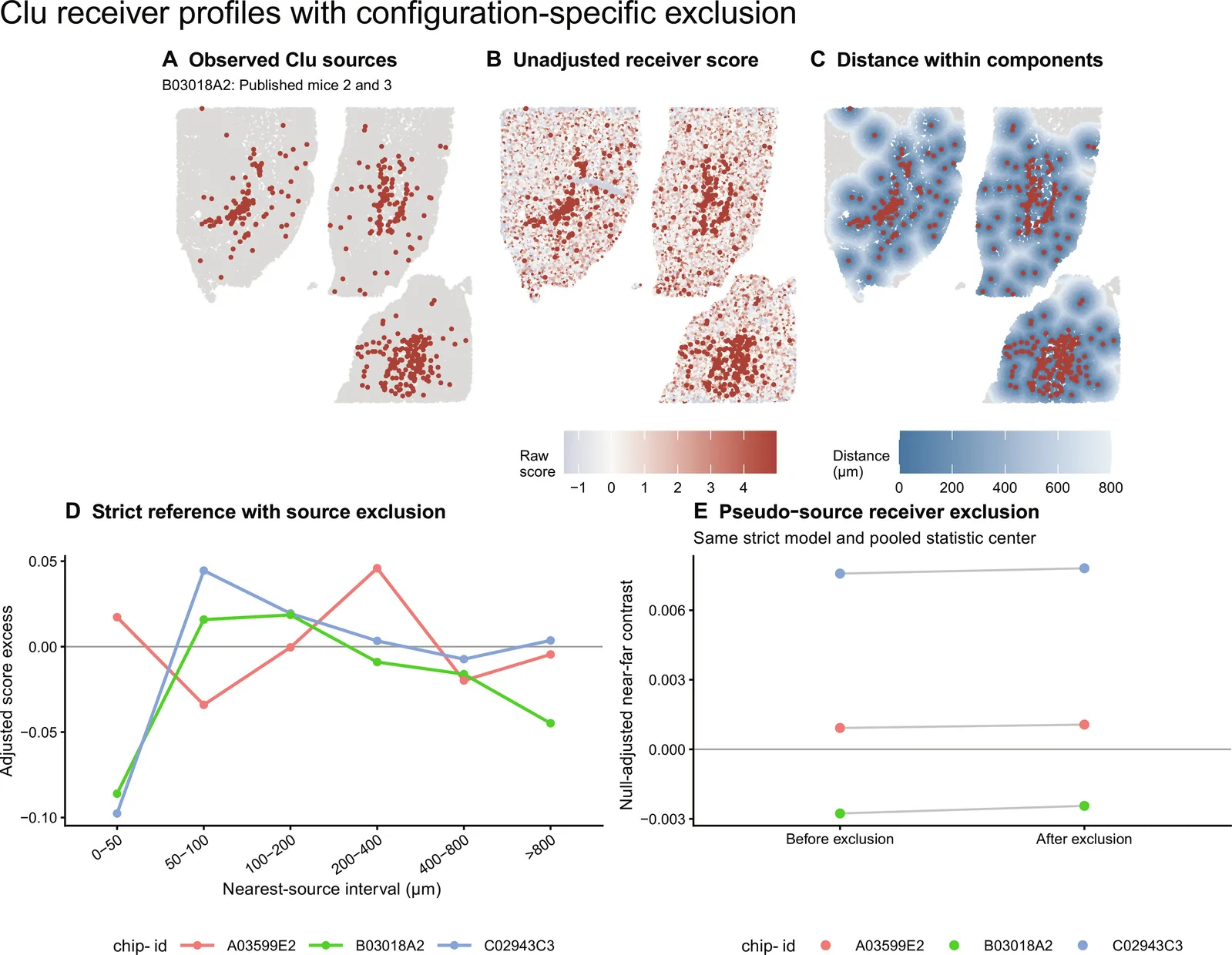
The *Clu* receiver score depends on source and background controls. (A) Tissue coordinates and observed single-guide *Clu* sources in the source-rich pooled chip. (B) Unadjusted receiver program in that chip. (C) Continuous same-component nearest-source distance; receivers beyond 800 μm are background and sources are overlaid. (D) Adjusted, source-reference-subtracted profiles in all 3 chips. (E) Chip-level strict-reference effects before and after excluding pseudo-source positions from each reference receiver contrast, with background model and placement bank fixed. Panels (A) to (C) show the pooled middle chip. The 3 chip units span 4 published mice; the middle chip does not provide separate mouse estimates.

The source numbers were 29, 419, and 60 across the 3 chips, distributed across 20, 241, and 42 connected source clusters, with largest cluster sizes of 5, 16, and 10. These differences in sampling, tissue composition and source placement help explain the large chip differences; receiver counts cannot compensate for missing independent source contexts. The original comparison changed both background adjustment and null geometry, so it does not isolate the contribution of either control. The control comparisons below examine these factors separately (SM12 and SM14). The adjusted score was not exceptional under the stated reference. Unstable program transfer, signal absorption by adjustment, source selection, and limited independent coverage prevent attributing that result solely to removal of a false biological effect.

A full-chip control comparison held source banks fixed across raw, identity, library-smooth, and full generalized additive model (GAM) score adjustments, with a separate global-linear branch (SM14 and Fig. S24). Cluster-reference fractions changed from 0.01/0.17/0.06 without adjustment to 0.78/0.91/0.99 with full adjustment across the 3 chips. Clustering was not uniformly more conservative than source-count matching. Moreover, 84.6%, 75.1%, and 62.6% of *Clu* near-window receivers were also within 100 μm of another detected single-guide source, including control guides. This detected spatial overlap limits attribution to *Clu* alone, without establishing a molecular effect of the other guides.

As a receiver-selection sensitivity, we excluded receivers within 100 μm of another detected single-guide or multiple-guide cell, retaining the original GAM and reference bank. The 3 reference fractions became 0.35, 0.05, and 0.54; the pooled middle chip lies at the nominal reference boundary in this restricted population. An mSafe-position comparison gave frozen-GAM fractions 0.39, 0.67, and 0.07. These additional diagnostics show sensitivity to placement and eligibility, without establishing a *Clu*-specific effect, an unexposed receiver population or empirical assay calibration (SM15; Table S18).

### Known-effect benchmarks show conditional detection and calibration limits

Fourteen synthetic scenarios with 100 repeated datasets each crossed 4 source references with no, linear, and GAM background adjustment (199 configurations per comparison; SM12). Under the baseline zero-effect setting, cluster-size placement with GAM rejected 1/100 datasets (95% Wilson interval 0.18% to 5.45%); at unit effect it detected 74/100 (64.6% to 81.6%). Disconnected tissue gave 10/100 zero-effect rejections (5.5% to 17.4%). Across all 168 zero-effect scenario/background/reference combinations, 24 Wilson lower limits exceeded 5%; these include deliberately mismatched procedures rather than 24 failures of a single default. The original runs condition on a fixed configuration bank per scenario. A separate known-assignment benchmark redraws the bank in every repetition and compares the same continuous endpoint under design-matched and cluster-only source laws: The matched law gives 1 to 7/100 zero-effect rejections across 6 settings (SM14). Neither simulation makes the unknown source-selection law of the real assays exchangeable.

For a direct injection check, we retained the observed B03018A2 *Clu* sources and tissue coordinates and sampled a receiver subset. Without injection, the GAM/cluster reference fraction was $P_{\mathrm{ref}}=0.775$. Adding a unit-height response with a 150-μm exponential decay scale produced source-reference-adjusted effects of 0.608 with the preinjection background fixed and 0.469 after refitting background, both $P_{\mathrm{ref}}=0.005$. Refitting retained 73.7% of the observed near–far contrast gain before source-reference subtraction at this decay scale and 63.3% at 600 μm, demonstrating that adjustment can absorb a genuine source-aligned response. A further 25/50% source-thinning check retained detection in this example but changed the estimated contrast nonmonotonically; it did not model dropped sources entering the receiver pool (SM12). These single-chip deterministic sensitivities are not power estimates or biological ground truth for *Clu*.

Separately, native Squidpy 1.6.5 neighborhood enrichment was run on the complete exported *Clu* chip [20]: 419 sources and 119,664 guide-undetected receivers, using a fixed within-component 6-nearest-neighbor graph and 499 label permutations. The high-score receiver class, defined by the overall receiver median, was enriched next to *Clu* sources (native count 1,576; *z* = 4.87), whereas the low-score class was depleted (1,247; *z* = −5.81); source–source adjacency was also enriched (130 directed incidences; *z* = 26.35). The strict continuous-score diagnostic in this chip gave $P_{\mathrm{ref}}=0.91$. These results address different endpoints and conditioning assumptions: categorical adjacency under random labels versus an adjusted near–far score under clustered sources. Receiver downsampling changed the native high-score result to depletion, illustrating joint sensitivity to receiver sampling, graph construction, and the sample-specific score threshold (SM12). The application programming interface supplies counts and *z*-scores, not empirical *P* values; neither comparison establishes biological ground truth or universal software superiority.

A count-level benchmark explicitly modeled guide detection and receiver contamination rather than merely thinning sources. False-negative true sources entered the observed receiver pool, and 4-gene negative-binomial counts separated direct source changes from an extrinsic field. At mean guide sensitivity 0.5025, mean receiver contamination was 3.82%. With no extrinsic field, GAM/cluster testing rejected 33/100 observed-label datasets when hidden sources retained a direct expression effect, versus 3/100 after removing that effect; oracle receivers also gave 3/100. Increased observed-label rejection with a positive field is therefore not calibrated power. All 57,600 records, 576 summaries and unavailable outcomes are retained (SM13). This identifies a mechanism of false receiver attribution in the stated model, not the real assay’s detection sensitivity or error rate.

Native Squidpy was additionally run on 600 generated cases using a categorical adjacency endpoint (SM13). Under its fixed 2-sided Gaussian z-score rule, ideal guide detection produced 87/100 zero-extrinsic-field exceedances, all negative adjacency depletion. Source clustering itself violates that categorical-label null, so these are not molecular-field false-positive rates. Native and continuous-score references answer different questions; their disagreement does not establish method superiority.

Detection in these benchmarks depended on the generating model, receiver definition and source reference; several tested settings remained poorly calibrated.

### Perturb-map stromal contrasts are descriptive at the animal level

We next analyzed Perturb-map lung tumors containing *Tgfbr2*-null and KP lesion annotations. Clone-labeled spots were sources; unlabeled or publisher-annotated “periphery” spots were receivers. Periphery can include a tumor edge and is not a proven tumor-free compartment. The specified stromal set included *Serpine1*, *Ctgf*, collagen, fibronectin, tenascin, tissue-inhibitor, and inflammatory-remodeling genes. Nine of the 10 requested symbols were available; *Ctgf* was absent under that symbol, and no alias was substituted. The mean profile around the 4 focal annotations was 2.10 robust score units at 0 to 100 μm, then declined across the successive 100–200, 200–400, and 400–800-μm intervals to 1.18, 0.50 and 0.11, respectively; beyond 800 μm, it was −0.13. These are observed score profiles, not independent estimates of a propagation radius (Fig. 4A to D).

**Fig. 4.**
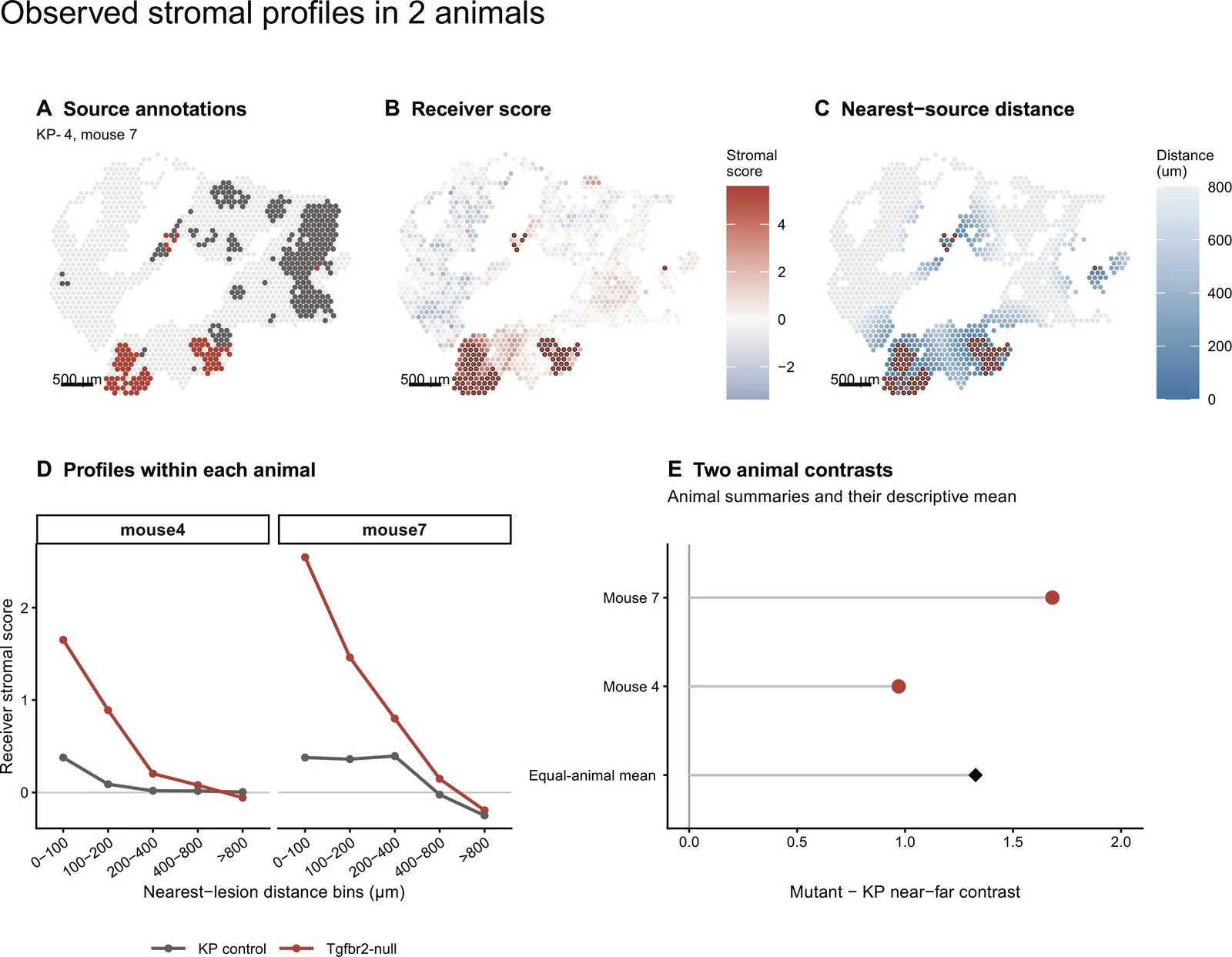
Observed stromal profiles in 2 Perturb-map animals. (A to C) Source annotations, receiver stromal score and nearest-source distance in KP-4 (mouse 7). Directly labeled lesions are excluded from receivers. (D) Profiles are averaged over lesions within section and then sections within animal, separately for the 2 lesion classes; KP-3 is excluded from both classes. Each animal is shown separately without lesion-based uncertainty bands. (E) The within-animal mutant-minus-KP near–far contrast for mouse 4 (KP-1/KP-2) and mouse 7 (KP-4); the diamond is their equal-animal descriptive mean. Four mutant and 10 KP annotations are nested in 2 animals, and same-mouse cross-sectional clone correspondence is unknown. No animal-population confidence interval or lesion-level significance claim is displayed.

Animal provenance changes the inferential interpretation. Gene Expression Omnibus (GEO) titles and the 3-mouse/4-section design support KP-1/KP-2 as mouse 4, KP-3 as mouse 5 and KP-4 as mouse 7 (Methods). The focal comparison contains 4 mutant and 10 KP annotations: 1 mutant/3 KP in each of KP-1 and KP-2, and 2 mutant/4 KP in KP-4. KP-3 contains no focal mutant and is excluded. Thus only 2 animals contribute, and the 2 mouse-4 focal annotations are not verified distinct 3-dimensional clones.

We first averaged the mutant-minus-KP near–far contrast over sections within each animal, retaining all lesion annotations as dependent subsamples. The stromal contrasts were 0.9707 in mouse 4 and 1.6820 in mouse 7; their equal-animal mean was 1.3263 robust score units (Fig. 4E). Corresponding T-cell contrasts were −0.0635 and −0.2319, with mean −0.1477. These 2 observed animals show concordant directions but do not establish a population effect or relative T-cell exclusion. Animal-level uncertainty cannot be estimated reliably from these dependent lesion annotations.

The 240-label enumeration independently reassigned labels in the 2 sections from mouse 4 and therefore did not preserve possible cross-sectional clone-linked labels. Equal-animal weighting leaves this reference construction unchanged, so the resulting section-label fractions and family-wise adjustment do not support animal-level inference. Under the working assumption of independent, zero-centered symmetric animal contrasts, flipping each whole-animal contrast gives 4 sign configurations. The observed stromal mean and its complete sign reversal have equal absolute magnitude, yielding a 2-sided reference fraction of 2/4=0.50. This calculation reflects the limited resolution of 2 animals and is distinct from treatment-randomization inference.

KP boundaries retain real tumor context but do not balance lesion size, location, or receiver composition. A fully adjusted descriptive regression had opposite genotype-coefficient signs in the 2 animals, while coarse composition standardization and broad matching to real KP lesions retained both original stromal directions (SM7 and SM13). Rigid transport of complete observed footprints produced 1,424 within-mask placements, but only 1 of 14 annotations had any location/composition-matched position; the complete animal endpoint remained unestimable. The mask is an annotated capture domain, not a verified nonlesion or histological boundary. Positive sensitivity summaries therefore coexist with unresolved placement/state confounding and insufficient independent replication; size and composition may themselves be perturbation consequences.

### Specification and influence checks do not create additional animals

Four related program definitions and 16 distance specifications produced 64 correlated checks. Positive pooled contrasts describe stability of a summary within these same animals; they are not independent replications or a substitute for animal-level inference. Leaving out an animal leaves only one independent animal, with stromal contrast 0.9707 or 1.6820. Individual lesion deletion also changes the mix of dependent subsamples and is retained as an influence diagnostic, without inferential lesion-level *P* values in the main text. Both animals had positive stromal contrasts under every specification; equal-animal primary-program summaries ranged from 1.0228 to 1.7059. Complete animal-specific results and section-label sensitivity calculations are reported in Supplementary Methods SM7 and SM8.

Removing each of the 9 measured stromal genes in turn retained positive contrasts in both animals (equal-animal means 1.1775 to 1.5830). Removing all 5 matrix genes left a 4-gene primary score with mean 0.4726; a separately defined 5-gene nonmatrix target score also remained positive. Individual *Serpine1 and Spp1* contrasts reversed in mouse 7, so aggregate stability does not imply that every gene agrees. These fixed-window checks address gene influence and overlap within the same animals, without resolving cell mixture or validating a pathway (SM15 and Table S17).

### Molecular covariation does not identify a mediation mechanism

Section-adjusted ordinary-least-squares fits described lower source *Tgfbr2* expression (coefficient −0.226) and higher source TGF-*β* ligand score (0.531) in mutant annotations. The requested receiver target program combined *Serpine1*, *Ctgf*, *Smad7*, contractile, and matrix genes; the measured score used 10 of the 11 requested symbols because *Ctgf* was absent, without alias substitution. Receiver receptor score was included in the conditional model. The total target coefficient was 1.677, the source-ligand-to-target coefficient was 2.93, and the fitted product of the genotype-to-ligand coefficient (0.531) and that coefficient was 1.556. The matrix product was 2.675, exceeding the total coefficient (Fig. 5C).

**Fig. 5.**
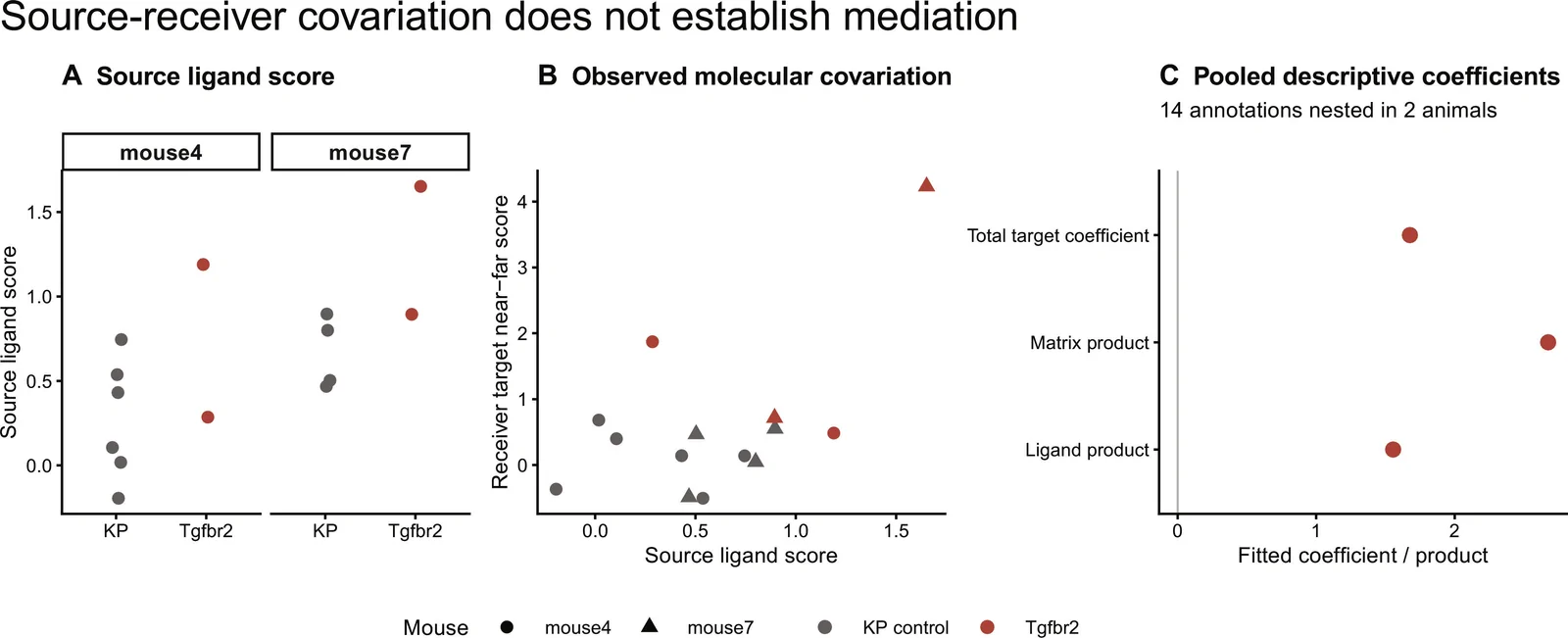
Descriptive source–receiver molecular covariation. (A) Source ligand scores for the 4 mutant and 10 KP lesion annotations, shown separately by animal. (B) Source ligand versus receiver target near–far score; symbols identify animals and colors identify lesion class. No regression confidence band treats lesion annotations as independent replicates. (C) Pooled section-adjusted ordinary-least-squares coefficient summaries, including the soluble-ligand product, matrix product and total target coefficient. They are descriptive fits to 14 annotations nested in 2 animals; no inferential error bars, permutation significance, or mediated percentage is claimed.

These descriptive coefficients summarize 14 lesion annotations nested in 2 animals. Section-wise assignment fractions, the 6-mediator false-discovery adjustment and section/class lesion bootstrap do not account for animal-level or possible same-clone dependence; their reference fractions and bootstrap percentiles are not used as significance tests or confidence intervals. A mixed model or cluster-robust standard error fitted to 2 animals would not supply the missing replication.

The matrix and receiver target share collagen, fibronectin, and tenascin genes and may reflect the same stromal mixture. The total and conditional regressions use different covariates, so total, direct, and indirect coefficients need not satisfy an additive decomposition. Shared composition, overlapping receivers, suppression, and mediator–outcome confounding remain alternative explanations. These analyses do not identify a mediated proportion, preferred transmission route or causal mediation effect; model formulas and section-level calculations are documented in SM8.

### External platform, injury, and clinical tests limit generalization

The external analyses use different source definitions and therefore test transportability rather than supply additional exchangeable primary replicates (Table 1; Supplementary Results and Figs. S6 to S13). In Perturb-DBiT, a program selected in Bru.LC.6 was frozen before the Bru.LC.8 *ATG4A* receiver test; the receiver contrast was not exceptional under its source reference ($P_{\mathrm{ref}}=0.994$). The final source Table S1 assigns both sections to block 33-1, making this a within-block section holdout. A version-frozen audit using MSigDB 2026.1.Hs reproduced selection of the positive TGF-β Hallmark program and the held-section result (498/501); the original run had not recorded the gene-set release. The fixed *Tgfbr2* program had only 15 and 12 source spots in 2 liver sections from the same published block MSTGN3R; their separate conditional source-placement fractions were $P_{\mathrm{ref}}=0.381$ and 0.614. Independently generated section nulls cannot provide a dependence-preserving joint animal test for this shared block. These tests do not establish replication and cannot distinguish low power from context dependence.

Composition-defined myocardial-infarction cores and high-inflammation skin spots are observational proxy sources. With the myocardial score denominator set to whole-transcriptome counts, the 4 patient-label values ranged from 0.215 to 0.588. Patient- or section-level comparisons did not establish a conserved persistent field; different wounds at different times do not identify propagation speed. The ordinal response-profile atlas contained 62 complete curves and resolved 2 descriptive shapes (mean silhouette 0.489), not distinct mechanisms. Its B1 to B5 labels denote ordered near-to-far intervals, not a common physical distance scale across assays. Reconstruction and active-bin agreement concern curve summaries, not recovery of anatomical barriers; the supplementary audit distinguishes descriptive checks from held-out evaluation.

The HCC feature analysis excluded direct proxy-source spots from receiver curves and slopes and removed the source-response variable from all predictors. Visium coordinates used an isotropic hexagonal embedding, with image-pixel coordinates as an independent check. The top-2% apoptosis proxy sources, programs, score standardization, ridge grid, and validation structure were held fixed. In 11 paired patients, including 1 liver-to-lymph-node sample pair, nested leave-one-patient-out (LOPO) SPF-only AUC was 0.000 with Brier score 0.3652; adding receiver features to direct features worsened internal Brier score from 0.0664 to 0.2062 (AUC 0.9667 to 0.7667). An intercept-only leave-one-out diagnostic also had AUC 0 because holding out a responder versus nonresponder changes the training class proportion. Scores were evaluated without sign reversal. A same-model nested leave-pair-out sensitivity, withholding 1 responder and 1 nonresponder together, gave SPF AUC 0.600 and combined AUC 0.867, below direct-only 0.967. This sensitivity assesses within-fit ranking rather than LOPO probability error; the 30 pairs reuse patients. Restricting to 10 patients with liver samples at both time points gave SPF/combined pairwise AUCs 0.625/0.875, below direct-only 0.958 (SM15).

In 7 locked external resections [40], SPF-only and combined-model AUCs were 0.6667 and 0.5833, versus 0.8333 for direct features. Cross-cohort program membership was harmonized to the 35 genes measured in both assays; *CD8B*, *LDHA*, and *BNIP3* were absent from the training assay. Primary external AUCs were unchanged; SPF Brier error was 0.2647, slightly worse than the training-prevalence baseline of 0.2586 despite outperforming the direct model’s probability error. A fixed 17,779-gene common-library-denominator sensitivity gave an external SPF AUC of 0.750, but its Brier score (0.2611) still exceeded that baseline; combined AUC remained 0.583. These results indicate measurement and cohort dependence. Pixel-coordinate sensitivity gave the same AUCs, while source-threshold/window changes exposed coverage failures (SM10 and SM13). Across the 11-patient training and 7-specimen external cohorts, the combined model did not show consistent incremental performance. Small cohorts, different pathological endpoints, leave-one-out class proportions and posttreatment features limit clinical biomarker interpretation.

## Discussion

The principal result is that receiver ascertainment, source-reference validity, and biological replication impose separate limits on spatial perturbation inference. A score can lose its distance association after adjustment without showing that the biological effect was false; a positive profile can survive sensitivity checks without becoming an animal-generalizable effect. *Clu* was not exceptional under the specified reference, while training-only mouse holdouts left program transfer unresolved. Perturb-map stromal contrasts were positive in the same 2 animals, but dependent sections and unavailable matching support did not supply independent confirmation.

Spatial-domain methods represent expression and neighborhood structure [30,31], while classical spatial reference tests condition on selected features of point patterns or maps [27,28]. Controlled analyses identify 2 concrete failure mechanisms. Refitting a spatial background absorbed part of a deliberately injected source-aligned response, retaining 73.7% of its contrast gain at a 150-μm decay scale and 63.3% at 600 μm. Separately, missed sources entered the receiver pool with their autonomous expression changes: 3.82% contamination in the specified count model produced 33/100 zero-field rejections, compared with 3/100 when the autonomous term was removed. These concern stated generators, not measured public-assay error rates. Source-count constraints and nominal cluster-size matching are also incomplete descriptions of placement: They preserve neither exact geometry nor all niche selection. The workflow identifies these dependencies rather than guaranteeing that their adjustment recovers a causal field.

The observed *Tgfbr2* stromal profiles are compatible with the phenotype reported by the source study [17] but do not establish an animal-generalizable receiver field. Independently reallocating labels in repeated sections can violate the joint structure of a shared, unobserved clone [29]. Equal-animal weighting changes the estimand, not the exchangeability scheme. The 2 animal contrasts are therefore summarized descriptively; section-label significance, lesion-bootstrap confidence intervals and mediation claims are not supported. Source geometry, cell mixture, and shared program genes remain competing explanations. Causal mediation would require additional identification assumptions beyond these descriptive regressions [41].

Neither the 4 lesion annotations, the 64 correlated specifications nor lesion-deletion fits create additional animals. With 2 contributing animals, a whole-animal sign-flip reference has only 4 configurations and cannot deliver a 2-sided value below 0.50; it additionally requires a sign-symmetry assumption that is not guaranteed by observational lesion labels. The Perturb-DBiT focal pairs each share a tissue block and provide no independent-animal confirmation. Reliable biological confirmation requires independently sampled animals with traceable clone/section identities and appropriate source/receiver controls [42,43].

Prior experiments provide biological context without independently validating the field measured here. Integrin-mediated activation of latent TGF-*β* and contraction-dependent release of matrix-stored TGF-*β* provide experimentally demonstrated routes for local extracellular regulation [44,45]. In colorectal cancer models, tumor-derived TGF-*β* can act through stromal programs to promote metastasis initiation [46]; TGF-*β* signalling has also been implicated in immune evasion and T-cell exclusion in colorectal and other tumor settings [47,48]. Pancreatic cancer studies identified spatially distinct inflammatory and myofibroblastic fibroblast populations [49], with opposing interleukin-1 and TGF-*β* signals helping to shape those states [50]. These findings support testing both receiver identity and receiver state, rather than interpreting all fibro-stromal enrichment as the same mechanism.

The negative transport analyses further constrain generalization. Composition-defined injury cores are not equivalent to observed perturbation sources, and sections collected at different times do not track the same tissue longitudinally. The failure to recover conserved myocardial or skin fields may reflect source misclassification, limited controls, section-to-section sampling, or genuine organ specificity. Similarly, the HCC embedding did not add stable predictive information beyond direct or pretreatment features and did not establish consistent incremental performance in the locked external cohort. The source HCC studies also differed in treatment regimens and pathological-response definitions [40,51,52]. A common coordinate system for fields can organize heterogeneous datasets without implying that they share a causal kernel.

Several proposed quantities remain nonidentifiable. Snapshot-based single-cell profiles provide incomplete information about dynamic responses [33]. Directional amplitudes could be estimated within some Spatial Perturb-Seq chips, but preferred axes could not be aligned across tissues. Clone boundaries provided controls, not annotated anatomical barriers, so boundary penetration was not measured. Injury time points came from different specimens, preventing within-tissue speed or persistence estimates. Cell trafficking, vascular transport, and contact-dependent signalling were not experimentally isolated. The 2 ordinal profile groups summarize curve shapes without identifying their mechanisms. The atlas retains these evidence limits and unavailable measurements.

Machine-learning approaches model perturbation responses in complex cellular systems [32]. Generative digital twins and multiscale multiomics models aim to simulate biological systems and predict their responses [53,54]. Receiver-field tests could provide a diagnostic for such models, but the present transportability results do not validate a generative tissue model. Static endpoint maps also do not identify whether final source geometry arose through clonal growth, migration, tropism, or selection.

Application requires 4 checks: receiver-score validity, contamination by undetected sources, support for the source reference, and replication at the biological sampling unit. When a check fails, interpretation is restricted to the observed score and reference (SM14). Computational diagnostics alone do not identify causality. Mechanistic inference requires receiver-side intervention, while temporal claims require repeated or otherwise causally aligned sampling.

The *Tgfbr2* reanalysis describes a local, control-lesion-relative fibro-stromal profile in 2 sampled animals and does not establish animal-level significance. The *Clu* and external examples delimit what the same workflow can establish. Positive and negative results remain conditional on source ascertainment, program choice, exchangeability, and biological replication.

## Methods

### Study design, analysis units, and operational field definitions

The analyses comprise genetic perturbation examples, external assay tests, injury comparisons, and clinical prediction. These analyses were not prospectively registered. Inclusion required a readable expression matrix, spatial coordinates, and observed or proxy source labels. Analysis blocking followed the source design: chip for Spatial Perturb-Seq, animal-level descriptive summaries for Perturb-map, patient for paired HCC and MI, and section for the skin and Perturb-DBiT stress tests. Biological replication was assessed separately: 4 Spatial Perturb-Seq mice are partitioned across 3 chips, while the focal Perturb-map comparison has only 2 mice despite 3 informative sections. Cells and spots supplied spatial resolution but were not treated as independent biological replicates. This separation follows the experimental-unit principle underlying pseudoreplication control [42,43] and evidence that ignoring biological-replicate structure can inflate single-cell differential-expression discoveries [55]. Multiple-guide Spatial Perturb-Seq cells were excluded as sources, and 2 corrupted skin matrices were excluded after gzip integrity testing. Perturb-DBiT sections were not added to the frozen primary animal count because the public metadata included related models and adjacent-section designs.

Within this design, response classes were defined consistently across datasets wherever the available spatial resolution permitted. Direct response denoted molecular change in guide-positive or clone-labeled source units; neighbor response was evaluated in eligible nonsource receivers within 100 μm of the nearest source; and bystander response referred to receivers 100 to 400 μm away. A systemic response required support beyond 800 μm or across disconnected tissue components; otherwise these regions served as distance-defined background references. For visualization, sources formed the perturbation core, 0 to 100 μm the interface, 100 to 400 μm the response border, 400 to 800 μm the remote niche and distances beyond 800 μm the distance-defined background region; absence of a biological effect was not established. These labels were operational and did not imply histological boundaries where none were annotated. Platform-specific primary endpoints could pool adjacent operational classes when required by the assay design; such pooled windows did not redefine the general neighbor and bystander categories.

### Program learning and projection

Spatial Perturb-Seq data in the Gene Expression File (GEF) format from GSE274447 were read with rhdf5. Exactly one detected sgrna- feature defined a single-guide source; no detected guide defined a receiver candidate; multiple-guide cells were excluded. File resolution was 500 nm per native coordinate. Connected tissue was identified by 8-neighbor occupancy on 500 native coordinate units, equivalent to 250 μm, so distances did not cross gaps between disconnected pieces. This grid defines tissue connectivity and does not aggregate cell-level expression.

GSE274058 and GSE274447 are the dissociated single-cell and Stereo-seq arms of the same hippocampal perturbation study [16], using a pooled barcoded adeno-associated virus library covering 18 loci (17 genes and the mSafe safe-harbor control). In the source experiment, a perturbation barcode was linked to a construct carrying 3 guides. Here, “single-guide” operationally means 1 detected annotated guide/target feature in the processed data, rather than proof of 1 physical editing RNA per cell. These annotations report perturbation-linked barcode detection, not independent verification of all guides or editing. Dissociation and spatial capture recover different cell populations; fragile neurons can be underrecovered by dissociation. Single-cell programs used GSE274058. GEO describes 5 dissociated reference mice as biological replicates across 3 batches. Their sample labels B1-1, B1-2, B2-1, B2-2, and B3-1 denote batch and within-batch mouse; they are separate assay samples from the spatial measurements, but public records do not establish whether the 2 assay arms share animals. They are not paired measurements of identical cells or a verified animal-disjoint training/test split. Within each mouse, expression-state matching selected distinct guide-undetected controls. For gene *g*, pseudobulk counts obeyed the edgeR negative-binomial model [56], with effective library sizes incorporating trimmed mean of M-values normalization:

$$C_{\mathrm{gmq}}∼\mathrm{NB}\left(\mu_{\mathrm{gmq}},\phi_{g}\right),\;\text{log}\;\mu_{\mathrm{gmq}}=\text{log}\;L_{\mathrm{mq}}^{\mathrm{TMM}}+\alpha_{g}+\eta_{\mathrm{gm}}+\beta_{\mathrm{gp}}1\left\{q=p\right\}.$$ (2)

Here, m indexes mouse, q focal-guide or matched-control pseudobulk, and $L^{\mathrm{TMM}}$ is the effective library-size offset. The fitted design was mouse_id+group; matching used transcriptional state, without an explicit identity regression coefficient. Gene filtering, quasi-likelihood testing, thresholds, control matching, and the mSafe blacklist are specified in Supplementary Methods. Weights $w_{\mathrm{gp}}$ were retained signed log-fold changes. Receiver scores were weighted log-normalized expression sums divided by $\sum_{g}|{\;w}_{\mathrm{gp}\;}|$, then centered and scaled using the median and 1.4826-scaled median absolute deviation of guide-undetected cells in the same chip (SD fallback if needed). The reference-to-spatial projection was held fixed in the reported spatial tests. The complete training-only preprocessing holdouts and the conditional weight-only diagnostic are specified in SM13 and SM2. The former rebuilt Seurat/Harmony in 4 training mice per fold and projected the query using fixed training principal component analysis loadings; its 2 estimable primary *Clu* no-immediate-early-gene AUCs were 0.531 and 0.359, with 3 unavailable mSafe folds. Neither diagnostic establishes stable program generalization.

Signed weights express concordance with the guide-associated reference response and are readily interpretable. They are not an optimal classifier: A covariance-aware discriminant would require reliable covariance estimation, and a neighboring cell may respond through a different program. Binary signed weights, shrinkage-weighted effects, and curated receiver-specific endpoints are alternatives requiring independent selection and sensitivity analysis. Failure of this projection does not exclude all possible receiver responses.

### Spatial receiver adjustment, contrasts, and nulls

For the strict *Clu* analysis, a Gaussian identity-link additive model was fitted to guide-undetected receivers separately in each chip, using each receiver’s unique molecular identifier count as a library-size covariate [57]:

$$Y_{\mathrm{ip}}=\alpha +\gamma_{c\left(i\right)}+f_{1}\left(\text{log}\left(1+{\mathrm{UMI}}_{i}\right)\right)+f_{2}\left(x_{i},y_{i}\right)+\epsilon_{i},\;\epsilon_{i}∼N\left(0,\sigma^{2}\right).$$ (3)

The implementation was mgcv::bam, method=“fREML”, discrete=TRUE, with *k* = 5 for library size and k=30 for the 2-dimensional smooth. Scores, rather than raw counts, were the response; there was no count offset, perturbation term, distance term, interaction, or observation weight in this adjustment. Receiver identity was projected using 25 reference markers per broad class. Each chip had a separate fit, with no within-fit chip or section coefficient. The GAM was fitted once and residuals held fixed across null configurations. The model is flexible enough to represent broad endogenous structure but can also absorb a genuine response aligned with that structure; this is an identification limit, not a guarantee of calibration.

For source set S, the unweighted near–far statistic was:

$$T\left(S\right)={\bar{r}}_{d\left(i,S\right)\leq 100}-{\bar{r}}_{400<d\left(i,S\right)\leq 800}.$$ (4)

Distances are in micrometers and use only same-component receivers. The original source-count screen used the unadjusted standardized program, *r* = *Y*, and 199 pseudo-source configurations. It preserved source counts in each component. The strict *Clu* analysis used adjusted residuals and 99 configurations, additionally matching nominal group sizes derived from observed sources linked within 100 μm. Clusters were rebuilt from nearby candidate locations, not translated as rigid observed shapes; exact shape and niche occupancy were not preserved. For the strict 2-sided source-placement reference, we used the plus-one counting convention [58]; let B denote the number of valid configurations:

$$\begin{array}{c} P_{\mathrm{ref}}=\frac{1+\sum_{b=1}^{B}1\left\{|T\left(S^{\left(b\right)}\right)-{\bar{T}}_{*}|\geq |T\left(S\right)-{\bar{T}}_{*}|\right\}}{B+1},\\ \;{\bar{T}}_{*}=\frac{T\left(S\right)+\sum_{b}T\left(S^{\left(b\right)}\right)}{B+1}. \end{array}$$ (5)

The source-count screen used $P_{\text{count}}=[1+\sum_{b}1\left\{|T\left(S^{\left(b\right)}\right)|\geq \right.\left.|T\left(S\right)|\right\}]/\left(B+1\right)$, without centering, and serves as an exploratory screen. For each reference draw, source locations, distances, window memberships, receiver exclusions, and contrasts are recomputed. Program weights, normalization, identities, tissue components, and fitted GAM residuals remain fixed. Each selected pseudo-source position is excluded from that draw’s receiver statistic, as observed detected sources are excluded from the observed statistic. With 99 valid configurations, strict values have resolution 0.01; when configurations are unavailable, both center and denominator use only valid configurations. The reported effect remains $T\left(S\right)-B^{-1}\sum_{b}T\left(S^{\left(b\right)}\right)$, distinct from the symmetric center used for the 2-sided reference. Here, $P_{\mathrm{ref}}$ denotes a finite-configuration conditional tail fraction. Common centering removes the observed/null centering asymmetry but does not make the approximate source generator exchangeable or provide an exact randomization theorem. Calibration remains conditional and fails in some benchmark settings (SM12 and SM13). In the real *Clu* analysis, observed guide-positive sources are outside the guide-undetected candidate pool, and cluster specifications are estimated from the observed source graph. Excluding each pseudo-source from its own statistic does not make this complete assignment procedure symmetric or recover a known randomization law. Consequently these fractions and their BH adjustments are descriptive reference diagnostics, not guaranteed type-I-error or false-discovery-rate control. The known-assignment rank argument in SM14 requires assumptions not supplied by this assay.

The final source publication assigns chip 1 to mouse 1, chip 2 to mice 2 and 3, and chip 3 to mouse 4 (Supplementary Data 3 of the original study [16]); Supplementary Fig. 7 of the original study [16] indicates that chip 2 contains 2 animals.Native GEF cell counts uniquely match the published chip columns: C02943C3, 39,862; B03018A2, 123,921; A03599E2, and 65,992. GEO titles the 3 assay records mouse 1/2/3, whereas the published grouping assigns 4 mice across the 3 chips. The analyses use the detailed animal grouping in Supplementary Data 3 of the original study [16]. The chips therefore span disjoint published animal groups, but chip 2 cannot be separated into mouse-level effects because no cell/section-to-animal map is supplied. The source supplementary table and figure legend also disagree on whether chip 2 has 2 or 3 sections, so physical section counts are not inferred from chip counts (Supplementary Methods SM1).

The source-count screen combined chip-standardized effects $z_{s}=\left[T_{s}-{\bar{T}}_{0s}\right]/\mathrm{SD}\left(T_{0s}\right)$ as $Z=\sum_{s}z_{s}/\sqrt{K}$, using a 2-sided standard-normal tail followed by BH adjustment [59] across 10 evaluable programs. This is a normal-score approximation, not an exact conversion of empirical chip *P* values; the published animal groups do not overlap between chips, but any other chip dependence or departure from the normal reference would affect this approximation. The strict analysis reported chip-wise reference fractions and their descriptive BH adjustments across the all-receiver endpoint and 6 identities within each chip. No strict cross-chip combined test was used. *Clu* was selected after the screen, so its subsequent audit is diagnostic rather than a fresh independent confirmatory family.

### Thresholds, competing guides, and platform transfer

The 100-, 400-, and 800-μm windows are operational scales chosen for this dataset, not measured diffusion lengths or prospectively registered biological boundaries. The 0- to 200-μm Perturb-map endpoint pools roughly 2 spot spacings to provide receiver coverage. Programs, grids, windows and smoothing dimensions were fixed for the analysis; the available records do not establish whether every choice preceded outcome inspection. The analysis is therefore not described as preregistered, and sensitivity results are reported without selecting post hoc cutoffs.

Each pooled-guide test estimates a marginal association with 1 guide. Guide-undetected receivers may be close to other guide classes or carry an undetected guide; neither competing exposures nor guide interactions were included in the frozen model. A conditional model would add independently defined exposure terms $E_{\mathrm{ip}}=\sum_{j\in S_{p}}h\left(d_{\mathrm{ij}}\right)$ for each guide and estimable interactions, or restrict analysis to nonoverlapping exposure regions. Strong colocalization can make those effects nonidentifiable. A single-chip or single-niche guide cannot support a general perturbation-specific conclusion regardless of cell count.

Cell-resolved coordinates can be analyzed directly without gridding expression; the grid above is only a tissue-mask device. Mixed-cell capture spots require spot-center distances, explicit source/receiver contamination cautions and, where supported, independently estimated cell fractions or within-type endpoints. Euclidean distance is an operational tissue distance, not a travelled molecular path. Spot size, guide capture, registration, and segmentation errors change distance uncertainty, apparent field radius and null exchangeability; thresholds and connectivity must be adapted to physical resolution rather than transferred numerically between platforms. Supplementary Methods provides the design/replication table and worked workflow.

### Perturb-map animal summaries and descriptive molecular fits

Perturb-map GSE193460 10x Hierarchical Data Format version 5 matrices, spot coordinates, and annotations were imported with rhdf5, Matrix and data.table. Clone-labeled spots were treated as sources and unlabeled or publisher-annotated periphery spots as receivers; periphery may include tumor-edge spots. Such spots contain mixed cells and are not proven tumor-free. For each spot, ${\bar{C}}_{i,G}$ was the mean count over available program genes and $L_{i}$ the whole-transcriptome library size. Scores were $\log\;\left(1+10^{4}{\bar{C}}_{i,G}/L_{i}\right)$, then robustly standardized within section using the reference population specified in SM7. Nearest-source profiles used 0–100, 100–200, 200–400, 400–800, and >800-μm bins. The frozen platform-specific primary endpoint pooled receivers at 0 to 200 μm, spanning the operational neighbor zone (0 to 100 μm) and the proximal portion of the bystander zone (100 to 200 μm); it was therefore an assay-specific contrast rather than a redefinition of those response classes. Lesion effects were defined as this 0- to 200-μm receiver score minus that at 400 to 800 μm. *Tgfbr2* and KP lesions were compared within section. GEO titles identify KP-1 to KP-4 as mouse 4.1, 4.3, 5.1, and 7.2; the source analysis code uses matching identifiers max41, max43, max51, and max72. Together with the stated 4-section/3-mouse design, these support a high-confidence reconstruction of KP-1/KP-2 as mouse 4, KP-3 as mouse 5, and KP-4 as mouse 7; an explicit definition of the suffix as section versus lobe was not found. The focal contrast excludes KP-3 and thus contains 2 animals, with 2 mutant lesion annotations per animal. Following the experimental-unit principle, we calculated lesion near–far endpoints, averaged mutant-minus-KP contrasts within each section, then averaged sections within mouse and mice equally. If $\Delta_{c}$ is the section contrast and $C_{m}$ its sections in mouse m, the descriptive summary is:

$$D_{m}={\left|C_{m}\right|}^{-1}\sum_{c\in C_{m}}\Delta_{c},\;\bar{D}=\frac{1}{2}\left(D_{4}+D_{7}\right).$$ (6)

No cell, spot, lesion annotation, or repeated section is counted as an additional animal. Possible cross-sectional identity of the 2 mouse-4 focal annotations remains unknown, so the section mean is a summary of observed tissue rather than a count of independent clonal interventions.

A whole-animal sign-flip resolution check enumerated $\frac{1}{2}\left(s_{4}D_{4}+s_{7}D_{7}\right)$ for $\left(s_{4},{\;s}_{7}\right)\in {\left\{-1,1\right\}}^{2}$. Its 2-sided reference fraction includes all 4 configurations and assumes independent, zero-centered symmetric animal contrasts [29]; animal-level treatment randomization is not reconstructed. With 2 animals, small-sample mixed models, cluster sandwich estimators, and lesion bootstraps cannot provide population precision. Specification and deletion analyses are descriptive.

The 240 section-wise label allocations, 2-program max-statistic adjustment, geometry/composition regressions, and 5,000 section/class bootstrap draws are reported as sensitivity calculations in SM7 and SM8. Their reference assumes no additional cross-sectional label constraints and does not preserve an unidentified shared clone. Accordingly, the label fractions, bootstrap percentiles and 6-mediator adjustments are not interpreted as animal-level *P* values, confidence intervals, or causal evidence. The lesion regression equations and fixed endpoints are documented; mediation products are descriptive and do not identify physical transmission or a mediated proportion.

### Perturb-DBiT audit and external field tests

Processed expression matrices, guide-count tables, and coordinate lists were downloaded from GSE319277. Analyses were restricted to tissue barcodes represented in the expression matrix. Guide-positive spots were excluded from receiver sets. For the *ATG4A* test, 8 fixed human Hallmark programs were scored in Bru.LC.6 and Bru.LC.8. Scores were adjusted for a 2-dimensional spatial smooth and a smooth of a multigene library-size proxy. Program identity and sign were selected solely by the direct source contrast in Bru.LC.6 and then frozen for the Bru.LC.8 receiver test.

For the *Tgfbr2* liver test, a fixed target program contained *Serpine1*, *Ctgf*, *Smad7*, *Acta2*, *Tagln*, *Timp1*, *Col1a1*, *Col1a2*, *Col3a1*, *Fn1*, and *Tnc*. Pseudo-sources were generated within the tissue mask by matching nominal group sizes from the observed 8-neighbor source clusters. Separately placed groups may remain connected. The *ATG4A* analysis used 500 pseudo-source configurations; the *Tgfbr2* analysis used 1,000 per section and separate section-conditional references. Independent product-placement draws do not preserve unknown shared-block dependence and therefore do not provide an animal-level joint test. The 8 Hallmark membership lists and scores were frozen in the audit using MSigDB 2026.1.Hs/msigdbr 26.1.0, reproducing the selected *ATG4A* program and its held-section result. These are fixed-program section stress tests, not independent-animal confirmation or a genome-wide discovery screen.

### Injury and clinical proxy fields

For MI, injury cores were the top 5% of spots with low cardiomyocyte and high myeloid composition. Frozen fibrotic, inflammatory, vascular, and regenerative programs were evaluated in receiver spots. The library denominator was each spot’s whole-transcriptome count (the published n_counts field, verified against raw-count row sums in all 27 sections). Scores average per-gene log-normalized counts over available program genes. MI coordinates were normalized to the section median nearest-neighbor spacing and multiplied by the nominal 100-μm Visium pitch; this is an assumed array scale, not an independently calibrated image ruler. Skin coordinates use the supplied full-resolution spot-diameter scale and nominal 55-μm spot diameter. Patient-label tests compared injury with control while preserving patient as the unit. For skin, the top 5% inflammation-program spots defined proxy cores. Its frozen score is log of the mean normalized count, rather than the mean of logged gene expression used for myocardial and HCC programs. Sample-level near–far effects were compared across unwounded, day-12, day-21, and day-28 sections; the 2 expression-corrupted sections were excluded before scoring.

In HCC, posttreatment spots at or above the fixed per-sample 98th-percentile apoptosis threshold were proxy killing sources, excluded from every receiver curve, slope, and near–far feature. The source-response score is not included in any clinical predictor. Predictive features include posttreatment direct intensity, pretreatment state, and 4 receiver-program slopes against $\log\left(1+d\right)$, where d is normalized nearest-source distance (SM10). Five-bin curves are descriptive rather than predictive inputs. Program memberships are fixed to the 35 requested symbols present in the paired training assay and all external samples; external-only *CD8B*, *LDHA*, and *BNIP3* are excluded. Whole-assay library denominators remain the primary definition; a common uniquely mapped gene-universe sensitivity is reported separately (SM15). For Visium coordinates, we used x=array_col/2 and $y=\sqrt{3}\;\text{array}\_\mathrm{row}/2$, dividing Euclidean distances by each section’s median positive nearest-neighbor spacing. These are isotropic hexagonal-spacing units, not calibrated micrometers. Image-pixel coordinates supplied an independent sensitivity analysis; the uncorrected array-index metric is retained only as a historical comparison. A fixed exploratory grid varied source quantile 0.97/0.98/0.99 and radial-bin scale 0.75/1/1.25 without choosing a favourable specification; empty bins remained unestimable (SM13). Ridge logistic regression used an *L*_2_ coefficient penalty [60]. Nested LOPO validation refitted scaling and penalty selection in every training fold [61]. An additional nested leave-pair-out diagnostic refits the same procedure after withholding 1 responder and 1 nonresponder; only their within-fit ordering enters its AUC, without a Brier score or independent-pair interval (SM15).

The paired cohort contained 11 patients (5 source-labeled responders and 6 nonresponders), including patient 85567 with a liver-primary pretreatment sample and lymph-node-metastasis posttreatment sample; the 10 pairs with liver samples at both time points were evaluated separately (SM15). External GSE238264 contained 7 quality-controlled resection specimens (4 responders and 3 nonresponders), with preprocessing and coefficients locked before external evaluation. The binary outcome was $y_{i}=1$ for the source-provided responder label and $y_{i}=0$ otherwise. The paired study defined responders by major pathological response after 6 weeks of nivolumab with or without ipilimumab, using complete or greater than 70% tumor necrosis [51]. The external cabozantinib/nivolumab study used major or complete pathological response after its 8-week neoadjuvant regimen, with major response defined as at least 90% tumor necrosis [40,52]. These treatment, timing and threshold differences make the external analysis a cross-cohort stress test rather than validation of a harmonized clinical endpoint.

For each patient or external specimen, 1 held-out or locked probability ${\hat{p}}_{i}$ entered evaluation. The receiver-operating-characteristic curve varies the probability cutoff, plotting the fraction of observed responders called positive against the fraction of observed nonresponders called positive. AUC equals the fraction of responder–nonresponder pairs ranked correctly, with half credit for ties (30 pairs internally; 12 externally). Accuracy was $N^{-1}\sum_{i}1\left\{1\left({\hat{p}}_{i}\geq 0.5\right)=y_{i}\right\}$, and the Brier score was $N^{-1}\sum_{i}{\left({\hat{p}}_{i}-y_{i}\right)}^{2}$; these metrics concern clinical labels, not known true or false propagation. External AUCs were tested by exact outcome-label permutation (the Mann–Whitney ranking test), preserving responder counts. Full metric formulas and cohort provenance are supplied in Supplementary Methods. Direct posttreatment features are retrospective response correlates, not pretreatment predictors.

### Archetype analysis and leave-out validation

Complete profiles were aligned only by the order of 5 near-to-far intervals B1 to B5. Original interval labels and coordinate units are retained; HCC distances are expressed in hexagonal spacings. The atlas compares ordinal shapes, not physical field radii across tissues. Curves were centered within field and represented by local contrast, rank monotonicity, and interface-peak features. K-means from 2 to 6 clusters used 100 starts for selection and 500 for the final fit; the chosen solution maximized mean silhouette subject to a minimum size of 3 [62]. MI normalization and HCC receiver masks were used throughout atlas construction and evidence labeling.

Reconstruction used descriptive root mean square error, ordinal energy distance, active-bin agreement, quarter-peak bin-index discrepancy, radial rank concordance, and near-bin score discrepancy. Energy and quarter-peak calculations use indices 1 to 5 and are dimensionless; they do not measure an anatomical boundary, physical propagation radius, or directional vector. The target receiver identity is excluded from its scalar predictor. For Perturb-map animal exclusion, every training curve from the held-out mouse is removed, including other sections; 40 annotation curves represent only 3 mouse groups. A separate program exclusion is labeled leave-program, not leave-niche. Matching uses lesion class/program when available, with the recorded other-animal fallback for 2 Jak2 curves. No spatial-niche holdout or lineage-independent validation is claimed. Zero-shot dataset/platform/tissue-context checks use the remaining-atlas mean and remain descriptive. The tissue-context exclusion pools HCC patient tissues, including the nodal sample, and is not a pure anatomical-organ holdout.

### Software, reproducibility, and statistical reporting

The biological analyses, synthetic validation and figures used R; the native neighborhood comparator used Python and Squidpy 1.6.5. Seeds, package versions, parameters, input provenance, and compact numerical inputs are documented in SM11 to SM15 and the public repository specified in Data Availability. Full-chip and sampled native graphs are regenerated from the shared cell inputs using the repository scripts and recorded dependencies. Core packages included data.table, Matrix, rhdf5, RANN, igraph, mgcv, edgeR, msigdbr, ggplot2, and patchwork. Finite-configuration references state their null assumptions and denominator. No prospective power calculation was performed for the reused public datasets.

## Ethical Approval

This study was a secondary analysis of deidentified data obtained from publicly available datasets. No new human participants were recruited, and no new identifiable private information or biospecimens were collected. Therefore, no additional institutional ethics approval or participant informed consent was required for the present study. The ethics approvals and consent procedures governing the original collection of the human data are described in the corresponding source publications.

## Data Availability

Spatial Perturb-Seq and matched Perturb-seq data are available from GEO under GSE274447 and GSE274058. Perturb-map is available under GSE193460. Perturb-DBiT expression and guide data are available under GSE319277; its adjacent Xenium dataset is GSE319123. Human myocardial-infarction data are available from Zenodo record 6578047. Skin-wound data are available under GSE278169.The paired HCC data are available from Zenodo record 19123188, and the external cabozantinib/nivolumab cohort from GSE238264. Perturb-FISH [63] files are deposited in the Brain Image Library under digital object identifier (DOI) 10.35077/ace-gem-get; they were not included because the repository transfer endpoint was unavailable during acquisition. The numerical implementation and 8 compact inputs are publicly available at https://github.com/azusakou/spatial-perturbation-inference.
