# Supplementary material for: Source Geometry and Receiver Ascertainment Limit Spatial Perturbation Inference
Source: Comput Struct Biotechnol J. 2026 Nov 10;35(1):0258. doi: 10.34133/csbj.0258 (PMC13748624; doi:10.34133/csbj.0258)
Supplement: Supplementary file 1 [file csbj.0258.f1.pdf]

Source geometry and receiver ascertainment limit spatial perturbation inference

Supplementary Information

Zongnan Lyu[a], Qi Yu[a], Renyu Yang[a], Chunxue Shao[a], Guang Yang[a] and Ziheng Wang[a,*]

[a]*Division of Computational Biology, Chinese Center of Exercise Epidemiology, Northeast Normal University, Changchun, 130024, Jilin, China*

A B S T R A C T

This Supplementary Information provides extended methods and results, dataset and validation summaries, sensitivity analyses, quality-control audits, and external stress tests supporting the main article.

Contents

Supplementary Methods **2**

Supplementary Results **14**

Supplementary Figures **22**

*Corresponding author: Ziheng Wang.

wangzh654@nenu.edu.cn (Z. Wang)

ORCID(s): 0000-0002-3953-0493 (Z. Wang)

Supplementary Methods

SM1. Estimand, terminology and inferential scope

A source is a measured cell or spot assigned to the perturbation or lesion being tested. A receiver is an eligible measurement outside the source set used in that configuration. “Guide-undetected” means that no guide transcript passed the detection rule; it does not establish that the cell was untransduced, unedited or unexposed. A response is a measured programme score, and a field is the conditional mean of that score as a function of distance from the observed source set. Let R_c^0 be the observed receiver pool and $R_c(S) = R_c^0 \setminus S$, with subtraction applying to measurement units shared by the pool and source configuration. An operational radial field is

$$F_{pc}(I;S) = \frac{1}{|R_c(I;S)|} \sum_{i \in R_c(I;S)} Y_{ip}, \qquad R_c(I;S) = \{i \in R_c^0 \setminus S : d(i,S) \in I\}. \tag{1}$$

Here p indexes a perturbation programme, S_{pc} is its observed source set, I is a distance interval, and Y_{ip} is the fixed programme score or its adjusted residual, as specified below. Empty or insufficiently covered intervals are not estimable. The operational hypothesis is whether a near–far contrast is unusual relative to a stated spatial-source null or a real control-lesion comparison. A field alone does not identify molecular transport, propagation speed, lineage expansion or cellular movement. We use transportability for transfer of a response definition between datasets; physical transport denotes material or signal movement and requires additional mechanistic evidence. Tissue reorganization can include changes in composition as well as state.

For observed contrast A_0 and B finite source-reference contrasts $A_1, \dots, A_B$, the current two-sided comparison uses the common centre $A_* = (A_0 + \sum_{b=1}^{B} A_b)/(B+1)$ for both observed and simulated absolute deviations (SM5). Descriptive excess remains $A_0 - B^{-1} \sum_b A_b$. This finite-bank symmetry and configuration-specific receiver exclusion do not demonstrate that observational source locations are exchangeable.

The source-count null, cluster-size-matched null and control-lesion null answer different conditional questions. In the pooled screen, the comparison is conditional on the measured tissue mask and eligible receiver landscape. In Perturb-map, the comparison is conditional on the observed lesions and section-specific class counts. Their interpretation depends on explicit exchangeability assumptions, which are not established merely by enumeration; enumerating observational lesion labels is not proof of random lesion placement. Repeated sections from one animal do not constitute independent animal replication. The primary Spatial Perturb-Seq analysis has three chip blocks spanning four published mice, and the descriptive Perturb-map comparison has four mutant lesions in three informative sections from two mice.

An accession-to-file provenance audit retrieved the final source supplements, GEO SOFT, BioSample and SRA metadata and read native GEF cell counts. Supplementary Data 3 of the Spatial Perturb-Seq source assigns Chip 1 to mouse 1, Chip 2 to mice 2 and 3, and Chip 3 to mouse 4. The GEF counts 39,862 (C02943C3), 123,921 (B03018A2) and 65,992 (A03599E2) uniquely match those three columns. Supplementary Fig. 7 and the published author response also confirm two mice in Chip 2 and four mice overall. GEO titles the three records Mouse 1 (2 sections), Mouse 2 and Mouse 3 and describes three mice. The records differ; the animal grouping follows the detailed assignment in Supplementary Data 3 rather than treating a GEO assay title as a unique animal. Chip 2 has two sections according to that table but three according to the figure legend; its precise section count remains unresolved. No native GEF attribute, official example-object metadata or public accession record supplies the cell/section assignment within Chip 2. Connected tissue components are geometric units and are not assigned to its two animals. The published groups are disjoint between chips, but four mouse-level effects cannot be reconstructed from three aggregated chip effects.

For the dissociated reference, GSE274058 explicitly describes five mice as biological replicates in three batches; its five sample titles specify Batch 1 mouse 1/2, Batch 2 mouse 1/2 and Batch 3 mouse 1. This supports the five-mouse pseudobulk blocking. The local `mouse_id` is filename-derived and `orig.ident` is parsed by Seurat from renamed cell identifiers; neither local field independently verifies animal identity. The source supplementary figure describes five mice pooled into three experiments, consistent with that reported batch structure but without an inter-assay animal linkage. Different BioSample/SRA accessions establish different assay records, not different animals. Whether any reference mouse also supplied spatial tissue therefore remains unknown; no animal-disjoint validation claim is made.

For Perturb-map, the GEO titles are mouse 4.1, 4.3, 5.1 and 7.2 for KP-1–KP-4, respectively. Together with the stated four-section/three-mouse design and the original author analysis, these support a high-confidence reconstruction of KP-1/KP-2 as sections from mouse 4, KP-3 from mouse 5 and KP-4 from mouse 7. An explicit definition of the

suffix as section versus lobe was not located; the grouping is reconstructed from consistent titles, author-code identifiers and the three-mouse overall design, not from an independently supplied animal crosswalk. The focal comparison has two animals, not three independent section-level biological replicates. The reconstructed assignments and unresolved metadata conflicts are documented above. The animal crosswalk is also retained as `animal_crosswalk` in the public repository input `data/endpoints.rds`; the repository's `DATA_SOURCES.tsv` identifies the public study records.

SM2. Programme learning from dissociated Perturb-seq

GSE274058 and GSE274447 are the dissociated single-cell and Stereo-seq arms of the same published hippocampal perturbation study, sharing a pooled barcoded AAV library covering 18 loci (17 genes and the mSafe safe-harbour control). Each perturbation barcode was linked to a three-guide construct in that experimental design. Operational single-guide/multiple-guide calls here refer to one or several detected annotated guide/target features in the processed files, not a count of physical editing RNAs within a cell. The feature annotations report perturbation-linked barcode detection and do not independently verify all three guides or editing. Dissociation and spatial capture recover different cell populations, including under-recovery of fragile neurons in the dissociated assay. Their dissociated and spatial measurements are separate assay inputs; "matched" here describes experimental context and the within-reference matching algorithm, not paired measurement of the same cells. Five dissociated mouse samples were processed; their biological-replicate interpretation is supported by GEO, as detailed in SM1. Guide features ended in `_gRNA`; a cell with one positive guide feature was assigned to that guide, one with several to the multiple-guide group, and one with none to the guide-undetected group. Gene-expression QC retained cells with 300–7,500 detected genes, at least 500 RNA counts and at most 20% mitochondrial counts. The retained reference contains 41,140 cells: 40,165 guide-undetected, 694 single-guide and 281 multiple-guide cells.

The reference used Seurat LogNormalize with scale factor 10,000; 3,000 vst variable features were scaled for a 40-component PCA (seed 42). Harmony adjusted the first 35 PCs for mouse identity, consuming the RNG state left by PCA. Neighbours and resolution-0.6 clustering used Harmony dimensions 1–30 (clustering seed 17); UMAP used the same dimensions and seed 17 only for visualization. The first 30 Harmony coordinates defined the matching distance. For each target and mouse, at least three single-guide target cells were required. Each target cell was greedily assigned its nearest unused guide-undetected control among the 50 nearest candidates in that same mouse, or all controls if fewer than 50 were available. The input cell order was retained; the algorithm did not globally optimize the matching or sort cells by matching difficulty. If the algorithm could not supply one unique control for every target cell, that mouse–target pair was omitted. Thus matched target and control pseudobulks contain equal cell numbers within each retained mouse. This is state matching, not an additional cell-identity covariate in the count model.

For gene g, mouse m and target/control group a, raw RNA counts were summed over the matched cells to give C_{gma}. edgeR fitted the paired negative-binomial quasi-likelihood model

$$C_{gma} \sim \mathrm{NB}(\mu_{gma}, \phi_g), \quad \log \mu_{gma} = \log L^{\mathrm{eff}}_{ma} + \alpha_g + \gamma_{gm} + \beta_{gp}\, \mathbf{1}(a = \mathrm{target}). \tag{2}$$

The effective-library-size offset L^{eff} includes TMM normalization. The R design is `~mouse_id + group`. Genes were filtered with `filterByExpr(min.count=5)`; dispersions and quasi-likelihood fits used robust estimation. At least three paired mice were required. The programme included genes with gene-wise BH FDR below 0.10 and $|\log_2 \mathrm{FC}| \geq 0.5$, after exclusion of mitochondrial and ribosomal prefixes. The signed weight w_{gp} was the estimated $\log_2$ FC. Nine genes satisfying the same criteria for the mSafe guide were removed from every target programme; mSafe itself was not used as a primary learned programme. This subtraction is a conservative calibration against a control-guide-associated signature, not an estimate of a guide-detection error rate.

An alternative matching audit sampled equal numbers of guide-undetected controls within mouse and Seurat cluster, using the same paired count model. The cluster-matched Clu programme contained nine genes, whereas the nearest-state primary programme contained 12. These are alternative in-sample programme definitions, not held-out mouse validation. The primary Clu programme contains Wdr72, Junb, Prex2, Fosb, Fos, Samd4b, Apoe, Usp37, Csmd1, Maml2, Cadm2 and Kcnn2; all 12 weights are positive. Removing Fos, Fosb and Junb leaves nine genes. There were 78 Clu-guide cells across the five mice (9, 11, 20, 8 and 30), with an equal number of matched controls. Marker-based broad labels assign 60 of the 78 target cells to oligodendrocytes. This imbalance limits an interpretation as a universal cell-autonomous knockout programme.

The learned weights are therefore *guide-associated direct-response programmes relative to guide-undetected cells*. The control pool may contain edited cells with guide dropout and cells already exposed to neighbouring perturbations.

No independent gold-standard guide assay was available to estimate detection sensitivity. Neither matching nor the mSafe subtraction guarantees that guide-associated differences are exclusively cell autonomous. A receiver can also respond through genes absent from the source programme, so failure of a projected programme is not evidence against every possible non-cell-autonomous response.

For the revision, the exact input-order nearest-state matching was reconstructed from the original processed reference object, without relearning weights. All 78 Clu cells had unique controls, and broad-identity concordance was 100% in each mouse. Median 30-dimensional Harmony distances were 4.216, 3.042, 3.527, 3.933 and 4.307 in mouse order B1-1, B1-2, B2-1, B2-2 and B3-1. Standardized target–control differences in log library size ranged from −0.172 to 0.217, and those in detected-feature counts from −0.215 to 0.243. The denominator for each standardized difference was the square root of the mean of the target and control sample variances. These are training-balance diagnostics in the same state embedding, not held-out programme discrimination or proof against unmeasured confounding. Exact matched pairs for all eligible guide–mouse pairs are retained as `matching_pairs` in the public input `data/reference_counts.rds`; Supplementary Table 2 reports the Clu balance diagnostics.

Complete reconstruction from raw matrices. All five raw 10x matrix/feature/barcode triplets (15 files) were reread without loading the archived processed object. Guide calls, gene intersection, Seurat object construction, QC, normalization, variable-feature selection, scaling, PCA, Harmony, neighbours, clustering and UMAP were rerun. The 41,140 QC cells and 24,295 genes, ordered 3,000 variable features, normalized/scaled matrices, PCA/Harmony/UMAP coordinates and neighbour graphs matched the archived reference exactly. Input-order matching was independently rebuilt: all 671 target/control pairs across 71 mouse–guide comparisons, full-gene pseudobulks, 156 held-out Clu/control count profiles, all programme weights and 150 selected identity markers were unchanged. Refitting all 70,840 primary differential-expression rows and 40,282 holdout-training rows reproduced the recorded results. Pair-distance/library diagnostics differed only where prior values had been text-rounded, by at most 5.33×10^{-14}, without changing pair identity or fitted results. The dependent spatial scores and identity assignments therefore use identical inputs. `optional/reference.R preprocess` and `export` reconstruct the full reference and compact counts; their original public inputs and versions are documented. This replay provides computational verification, not new biological replication.

A conditional Clu weight-learning holdout relearned Clu weights and the mSafe blacklist using four mice at a time, excluding the held-out mouse from both count models. The jointly learned five-mouse Harmony representation and matching were held fixed across these folds. The complete raw reconstruction reproduced that shared representation; consequently, the held-out mouse was excluded from the count models but not from preprocessing. This is a conditional weight-learning holdout rather than a pipeline fitted independently within each training fold. Three of five folds were unestimable because the training-only mSafe comparison had fewer than three eligible paired mice; the Clu count model itself fitted in those folds. Programme size is unavailable when this calibration cannot be constructed. For the two estimable folds, the no-immediate-early-gene programmes contained eight and 19 genes and had held-out target-versus-control AUCs of 0.426 and 0.391. Their mean target–control differences divided by the held-out-control MAD were −0.349 and −0.386. These within-mouse descriptive tests do not establish a mouse-generalizable source-response programme. Folds without support remained unestimable, with no threshold relaxation or fallback programme. Full-gene pseudobulks, held-out cell counts and matching pairs are retained in the public repository input `data/reference_counts.rds`; `Rscript run.R reference` regenerates differential-expression tables, weights and fold metrics through `R/reference.R`.

A subsequent full-pipeline diagnostic rebuilt four training mice from raw counts in each of five folds. No held-out mouse entered gene filtering, QC fitting, variable-feature selection, scaling, PCA, Harmony, graph construction, clustering, programme DE or mSafe selection. The held-out mouse used fixed per-cell QC/normalization and the training gene means, SDs and PCA loadings; no unseen-batch Harmony prediction was assumed. The closest original route matched training cells in fold-specific Harmony and query cells within the frozen training-PCA projection; an auxiliary route used training PCA for both. All 17 non-mSafe guides, both gene versions and both routes were evaluated (340 rows). For the original 11 programmes, the primary no-IEG route was estimable in only 16/55 mouse–programme combinations; 33 lacked eligible mSafe training and six had additional target/gene/query support failures. Primary Clu no-IEG programmes had six and 13 genes, AUCs 0.531 and 0.359, and standardized differences 0.075 and −1.583; the auxiliary route gave AUCs 0.580 and 0.359. The other three Clu folds remained unavailable under the unchanged mSafe rule. SM13 gives all-fold support, exact protocol and independent checks. These results close the

shared-preprocessing validation gap without establishing stable mouse-generalizable programmes or spatial propagation. The original all-five-mouse projection weights are not replaced by whichever fold performs best.

SM3. Spatial guide calls, scores and receiver identity

Spatial Perturb-Seq cell-bin GEF records were analysed directly, without gridding the expression measurements. Spatial guide features began with `sgrna-` or `sgrna_`; underscores in this prefix were normalized to hyphens and names were lower-cased. Exactly one guide with positive counts defined an eligible source. Multiple-guide cells and all guide-positive cells were excluded from the receiver pool. The receiver pool R_c consisted of cells with no detected guide. No imputation of missing guides was performed. This receiver definition excludes detected co-targeted cells but cannot remove edited guide-dropout cells.

For count C_{ig} and recorded transcript count $L_i = \max(\text{expCount}_i, 1)$, spatial expression was $A_{ig} = \log(1 + 10^4 C_{ig}/L_i)$. For genes available on that chip,

$$Q_{ip} = \frac{\sum_{g \in G_p \cap G_c} w_{gp} A_{ig}}{\sum_{g \in G_p \cap G_c} |w_{gp}|}, \qquad Z_{ip} = \frac{Q_{ip} - \text{median}_{j \in R_c} Q_{jp}}{1.4826\,\text{median}_{j \in R_c} |Q_{jp} - \text{median}_{k \in R_c} Q_{kp}|}. \tag{3}$$

If this median absolute deviation was zero or non-finite, the receiver standard deviation was used. The signed weighted mean carries the source programme direction into receivers. It is an interpretable projection rather than a statistically optimal receiver-specific likelihood ratio: the analysis does not estimate precision weights, learn receiver-specific coefficients or infer that source and receiver responses must coincide. Equal-weight, rank-based and receiver-trained scores address different choices and require their own calibration.

The nearest-state procedure yielded 11 target programmes. The spatial projection evaluated a programme only where at least five observed sources and one measured programme gene were available. Ten programmes had at least one evaluable spatial chip. In particular, the dissociated label Dpp6 was not automatically aliased to the spatial feature `sgrna-dpp5`. Missing programme/source combinations are coverage failures, not non-significant results. Several programmes comprise only one gene, making their interpretation particularly sensitive to sparse detection.

Broad receiver identities were projected from guide-undetected reference cells. For each reference identity, up to 25 genes with positive specificity and at least 5% within-identity prevalence were selected, excluding mitochondrial, ribosomal and guide features. Specificity was the identity mean expression minus the maximum mean among other identities. Within each spatial chip, marker expression was centered and divided by its receiver standard deviation; identity scores were positive-specificity-weighted marker means. The largest score determined the identity, and the difference from the second largest was the confidence measure. A non-positive or non-finite margin was unresolved. Six broad categories were represented in the spatial projection: astrocyte, endothelial, microglia, oligodendrocyte, oligodendrocyte precursor and perivascular. These transferred labels are coarse covariates, not validated fine cell types; annotation error is a source of residual confounding.

SM4. Distance, background model and near–far statistic

All three GEF files record a 500-nm coordinate resolution. Distances therefore used $(x, y)_{\mu\text{m}} = (x, y)_{\text{native}} \times 0.5$. The tissue mask was represented by occupied bins of 500 native coordinate units, that is 250 µm, with eight-neighbour connectivity. Cells in bins connected through occupied neighbouring bins belonged to the same tissue component. This mask prevents a nearest-source search from jumping across disconnected pieces, but it is not a cell-interaction graph and does not impose a geodesic path around holes. For receiver i, $d_i(S)$ is the Euclidean distance to its nearest source within its own tissue component; the distance is infinite if its component has no source. Such receivers are excluded from finite near/far windows but retained in the > 800 background-profile bin. In B03018A2 this applies to 84 of the 8,994 receivers in that bin; they do not enter the primary near–far contrast.

The unadjusted screen used $Y_{ip} = Z_{ip}$. The strict Clu analysis removed Fos, Fosb and Junb, then fitted the following Gaussian identity-link model separately in each chip, only among eligible receivers:

$$Z_{i,\text{Clu}} = \alpha_c + \gamma_{c,h(i)} + f_c\{\log(1 + L_i)\} + g_c(x_i, y_i) + \varepsilon_i, \qquad \varepsilon_i \sim N(0, \sigma_c^2). \tag{4}$$

The R formula was `score ~ identity + s(log_lib,k=5) + s(x_um,y_um,k=30)` in `mgcv::bam`, with `method="fREML"` and `discrete=TRUE`. The default univariate and two-dimensional thin-plate regression-spline bases

were used; k denotes a basis-dimension bound, not the fitted degrees of freedom. The residual $Y_i = Z_i - \widehat{Z}_i$ was used downstream. There was no perturbation, distance, identity-by-distance interaction, library-size offset, observation weight, chip coefficient or section coefficient in this within-chip Gaussian model. Library size entered as a smooth covariate after normalization. Spatial correlation remaining in the residuals was not treated as independent-cell evidence: the downstream reference distribution came from spatial-source configurations.

The all-receiver and each identity-restricted contrast used

$$T_c(S;h) = \frac{\sum_{i \in R_c(h;S)} Y_i \mathbf{1}\{d_i(S) \le 100\}}{\sum_{i \in R_c(h;S)} \mathbf{1}\{d_i(S) \le 100\}} - \frac{\sum_{i \in R_c(h;S)} Y_i \mathbf{1}\{400 < d_i(S) \le 800\}}{\sum_{i \in R_c(h;S)} \mathbf{1}\{400 < d_i(S) \le 800\}}. \tag{5}$$

Here $R_c(h;S) = \{i \in R_c^0 \setminus S : h(i) = h\}$, with the identity restriction omitted for the all-receiver endpoint. Thus pseudo-sources are excluded from their own near/far statistic. For strict identity-resolved analyses, both intervals required at least 30 receivers. These cell counts are numerical stability gates, not independent-replicate counts or a power guarantee. Distance-bin means and their cell-level standard errors describe the measured landscape; they do not replace chip-level inference.

The 100-μm near radius and 400–800-μm reference interval were operational windows for a cell-resolved assay. Perturb-map used a 200-μm near radius to span multiple 55-μm capture spots. The intervals are neither estimated diffusion lengths nor universal biological boundaries. The response border (100–400 μm), remote interval (400–800 μm) and > 800 reference are descriptive compartments; the far interval need not be biologically unaffected. For the public-data reanalysis, programme and distance definitions were frozen within each reported projection/test pipeline, but were not prospectively registered before exploratory outcome inspection. The data tables and scripts distinguish the original screen, immediate-early-gene exclusion, strict follow-up and sensitivity grid.

SM5. Source nulls, quantities held fixed and multiplicity

The source-count/component generator samples pseudo-sources without replacement within each source-bearing tissue component, preserving the observed number in that component. It does not preserve source clustering, identity or finer source niche. In the pooled source-count screen, sampled positions remained in the receiver statistic. In the strict source-reference analysis, each configuration removes its pseudo-sources from its own receiver statistic, identity-restricted statistics and distance profiles, using $R_c^0 \setminus S^{(b)}$ as in SM1. An observed source was already outside R_c^0. Where a candidate pool also contains non-receiver units, only its intersection with R_c^0 is additionally removed. These constructions answer different conditional questions.

For the strict Clu null, observed source clusters were the connected components of a source-only graph linking distinct sources at Euclidean distances $0 < d \le 100$ μm, within a tissue component. The list of observed cluster sizes was retained for each component. In each null draw, the sizes were randomly ordered. For each size n, a still-unused guide-undetected seed in that component was sampled and its n nearest unused guide-undetected cells were selected. No cell was selected twice within a configuration. This preserves component-specific source counts and the requested sizes of the sampled groups. It does not enforce exact pairwise shape, require every sampled group to remain connected at 100 μm, prevent separate sampled groups from merging, or match source identity, niche, lesion size or distance to the tissue edge. "Cluster-preserving" is consequently shorthand for this cluster-size-matched construction, rather than an exact rigid translation of the observed point pattern.

The learned weights, measured expression, projected identities, score centre/scale, tissue components, candidate pool, nominal source-cluster-size list and original observed-receiver GAM residuals are held fixed. The GAM and smoothing parameters are fitted once on R_c^0; the statistical receiver mask changes with $S^{(b)}$, while the background fitting population does not. Thus selected pseudo-sources do not contribute to their own null contrast even though their original scores helped fit the frozen background. This is an explicit conditional analysis choice, not a complete exchange of source and receiver labels. Refitting after changing the fitting population, excluding source-near cells or introducing source distance would define a different analysis. Background fitting can absorb a real response correlated with position. Neither fixed adjustment nor matched source counts/sizes proves source–niche exchangeability or guarantees calibrated tail fractions. Failure to reject means only that the statistic is not unusual relative to the specified conditional reference; it does not establish absence of a biological response.

For the archived source-count screen, $B = 199$ configurations were evaluated and the reported chip P value was

$$P_{c,\mathrm{count}} = \frac{1 + \sum_{b=1}^{B} \mathbf{1}\{|T_c(S_c^{(b)})| \geq |T_c(S_c)|\}}{B+1}. \tag{6}$$

The source-count screen used an uncentered absolute statistic even when its null mean was non-zero. The strict analysis used a null-only centre. The primary two-sided reference uses one common centre for the observed and all valid simulated statistics. Let B_c be the number of finite simulated contrasts, and let $\overline{T}_{0c} = B_c^{-1} \sum_b T_c(S_c^{(b)})$. Then

$$T_{*c} = \frac{T_c(S_c) + \sum_{b=1}^{B_c} T_c(S_c^{(b)})}{B_c + 1}, \qquad E_c = T_c(S_c) - \overline{T}_{0c}, \tag{7}$$

$$P_{c,\mathrm{strict}} = \frac{1 + \sum_{b=1}^{B_c} \mathbf{1}\{|T_c(S_c^{(b)}) - T_{*c}| \geq |T_c(S_c) - T_{*c}|\}}{B_c + 1}. \tag{8}$$

The reported excess E_c is observed minus the simulated mean and is distinct from the centring used for the tail comparison. The three-chip Clu reference uses 99 configurations, giving a minimum step of 0.01 when all are valid. Invalid observed contrasts remain unavailable; finite-reference denominators and failures are reported, with no zero imputation or replenishment to reach a target denominator. Common centring removes a finite-bank asymmetry, and source exclusion aligns the within-configuration receiver definition; neither establishes exchangeability of this approximate observational source generator. Strict source-reference all-receiver fractions are 0.78, 0.91 and 0.99 (SM13), compared with 0.84, 0.91 and 1.00 under the archived source-count screen. These are conditional source-reference tail fractions, not exact-randomization probabilities.

Directional amplitude and descriptive radius. For each source configuration, θ_i was the azimuth from the nearest same-component source to receiver i. Receivers with finite scores and $d_i \leq 800$ µm were grouped using `findInterval` with knots 0, 50, 100, 200, 400 and 800 and `all.inside=TRUE`. Let $k(i)$ denote this radial stratum. After subtracting its mean residual, an unweighted regression without an intercept fitted

$$r_i - \overline{r}_{k(i)} = \beta_x \cos\theta_i + \beta_y \sin\theta_i + \varepsilon_i. \tag{9}$$

The amplitude was $A = (\beta_x^2 + \beta_y^2)^{1/2}$ and the preferred angle was $\mathrm{atan2}(\beta_y, \beta_x)$. At least 100 finite receivers and an estimable coefficient fit were required. The same calculation, distances and configuration-specific pseudo-source exclusions were applied in every reference draw. With B_A finite reference amplitudes, its one-sided reference fraction was

$$P_{\mathrm{ref,dir}} = \frac{1 + \sum_{b=1}^{B_A} \mathbf{1}\{A_b \geq A_0\}}{B_A + 1}. \tag{10}$$

This non-negative-amplitude comparison is uncentred and differs from the two-sided contrast reference above. Its inferential limitations are the same unknown source-assignment and ascertainment limitations.

The Clu profile used intervals ending at 50, 100, 200, 400 and 800 µm, plus > 800 µm. For each bin, excess was the observed mean residual minus the reference mean. If the largest finite excess exceeded $\sqrt{\epsilon_{\mathrm{machine}}}$, bins at or above one quarter of that peak were marked active. The descriptive radius was the largest finite upper edge among active bins. An active > 800 bin made the radius right-censored and numerically unavailable; no positive peak also gave an unavailable radius. This summary differs from the ordinal post-peak crossing discrepancy used in reconstruction (SM10) and does not estimate a diffusion length.

The source-count screen combined $z_{pc} = (T_{pc} - \overline{T}_{0pc})/s_{0pc}$ using $Z_p = \sum_c z_{pc}/\sqrt{K_p}$ over its K_p evaluable chips. The two-sided normal approximation was $2\Phi(-|Z_p|)$, followed by BH adjustment across the ten evaluable programmes. This is an equal-chip standardized-statistic combination; it does not transform the empirical P values into normal quantiles, and its normal/independence approximation is not an exact joint randomization test. The strict Clu analysis reports chip-wise conditional reference fractions and descriptive BH adjustment over seven endpoints within each chip (all receivers plus six identities). No independently calibrated strict cross-chip discovery P value is available. The six-identity exploratory family and ten-programme source-count screen are distinct multiplicity families.

SM6. Pooled exposure and design requirements

The guide-by-guide screen estimates a marginal association with distance to one observed guide class, conditional on the stated covariates and null. It does not estimate a partial effect conditional on distances to every other guide, nor a guide-by-guide interaction model. Guide-undetected receivers may be exposed to several observed guide classes, and unobserved edited neighbours are possible. Excluding detected multiple-guide source cells does not resolve overlapping extracellular exposures. Because programme-specific source classes occupy different niches and source coverage is sparse, simultaneous multi-exposure coefficients may be unstable or non-identifiable in these data. A field is therefore not assigned uniquely to one molecular route on this basis. A future multi-exposure analysis would need joint source coverage, an explicit distance/kernel design matrix and a null that preserves the joint exposure structure.

There is no universal minimum source count that makes a design identifiable. Five observed sources and 30 near/far receivers are computational gates used here. Independent animal/specimen replication, control support within each source-bearing context, multiple source clusters and overlap of source/receiver niches must be assessed separately. A guide in one specimen or one niche cannot support a context-general field even if a conditional chip-level test is small. Source clusters are geometric objects, not automatically independent biological replicates. The design table below states which units and controls are required before expanding a claim.

SM7. Perturb-map scores, lesion estimand and exact enumeration

Perturb-map GSE193460 is a Visium-based expression assay with source assignments from the study's lesion annotations. Its capture measurements may mix several cells. Coordinates were converted with 55/spot_diameter_fullres micrometres per image pixel. Spots annotated as a named Tgfbr2, KP, Ifngr2 or Jak2 lesion were sources of that lesion class. "Peripheral" denotes the supplied `periphery` annotation, rather than a new edge-distance threshold. Spots without a recognized lesion class, including peripheral and unlabelled spots, were eligible receivers. Thus a peripheral capture spot may overlap a histological lesion edge, and source exclusion does not guarantee a pure non-tumour cell population.

For a fixed programme G, the spot score was

$$Q_i = \log\left(1 + \frac{10^4}{|G \cap G_c|} \sum_{g \in G \cap G_c} \frac{C_{ig}}{L_i}\right), \qquad Z_i = \frac{Q_i - \mathrm{median}_{j \in R_c} Q_j}{\mathrm{MAD}_{j \in R_c}(Q_j)}. \tag{11}$$

Here R_c is the score-reference population of spots with a missing phenotype annotation or the supplied `periphery` label. This population coincides with eligible receivers in the four analysed sections. MAD includes the 1.4826 consistency factor and uses the reference-population standard deviation as fallback. This log-of-mean-normalized-count score differs from the mean-of-log-expression projection in Spatial Perturb-Seq. The stromal programme was Serpine1, Ctgf, Col1a1, Col1a2, Col3a1, Fn1, Tnc, Timp1, Tgfb1 and Spp1. The T-cell programme was Cd3d, Cd3e, Cd3g, Trac, Cd8a, Cd8b1, Lck and Lat. At least four measured programme genes were required for the primary receiver tests. The primary stromal set used nine of ten requested symbols in every section; Ctgf was absent under that symbol. Downstream, compact and target sets likewise use only available symbols. No Ctgf/Cyr61 alias substitution was made. The auxiliary mechanical set retained only Ankrd1/Amotl2 of four requested symbols and is not a validated mechanical-pathway endpoint; per-section memberships are determined from the measured symbols in the public input `data/perturbmap_spots.tsv.gz` by `R/perturbmap.R`. For each lesion ℓ with at least five assigned source spots, distance was the Euclidean distance to its nearest source spot center. It is a distance to the sampled lesion footprint, not a reconstructed histological boundary or distance from its centroid.

Let $e_{c\ell} = \overline{Z}_{d \le 200} - \overline{Z}_{400 < d \le 800}$ denote lesion annotation ℓ's fixed receiver endpoint in section c. Receiver windows can overlap, and annotations in different sections from one mouse can represent a shared three-dimensional clone. The four mutant and ten KP annotations are therefore dependent tissue subsamples: mouse 4 contributes KP-1/KP-2 (one mutant and three KP in each), and mouse 7 contributes KP-4 (two mutant and four KP). KP-3 (mouse 5) has no focal mutant and does not contribute.

The reported animal summary first calculates $\Delta_c = \bar{e}_{c,T} - \bar{e}_{c,KP}$, then $D_m = |C_m|^{-1} \sum_{c \in C_m} \Delta_c$ and $\overline{D} = (D_4 + D_7)/2$. Stromal $D_4 = 0.9706527750$, $D_7 = 1.6819931353$ and $\overline{D} = 1.3263229552$; T-cell $D_4 = -0.0635143923$, $D_7 = -0.2318877066$ and $\overline{D} = -0.1477010494$. These describe two animals, not fourteen independent lesions. Leaving out one mouse leaves the other's contrast and no replicated animal-level comparison. Choosing only KP-1 or

KP-2 from mouse 4 gives equal-animal stromal summaries 1.4937252692 and 1.1589206411; no section is selected because its result is stronger.

For an explicit resolution check, $T_s = (s_4 D_4 + s_7 D_7)/2$ was enumerated for all four $s \in \{-1, 1\}^2$. Sign flipping changes an entire animal, retaining all internal dependence. Its validity as a null reference additionally requires independent, zero-centred symmetric animal contrasts; that assumption is not established by observational clone labels. The stromal values are $-1.3263, -0.3557, 0.3557, 1.3263$, giving $2/4 = 0.50$ for the two-sided absolute reference (one-sided $1/4 = 0.25$). No two-animal sign-flip result can have a two-sided reference below 0.50. We use this calculation to expose resolution, not to claim treatment-randomized inference, sufficient power or absence of an effect.

The four programme definitions crossed near radii 100/150/200/300 μm, far starts 400/500 μm and far ends 800 μm or section maximum. All 64 stromal specifications were recomputed from the original spot data separately in each animal; both animal contrasts were positive throughout. The equal-animal primary-programme effects ranged from 1.0228 to 1.7059; the other programme definitions are tabulated with all outcomes. All 16 T-cell specifications had negative contrasts in each mouse. These are correlated descriptions of the same two animals. They do not add independent replication, validate source-niche exchangeability or establish a universal distance cutoff.

Section-label sensitivity calculations and inferential limits. The section-weighted statistic was $T_{\text{section}} = \frac{1}{3}\sum_c \Delta_c = 1.2077662284$, assigning mouse 4 two thirds of the weight. All $\binom{4}{1}\binom{4}{1}\binom{6}{2} = 240$ section-wise class-count-preserving assignments were enumerated. The observed allocation was included, without a Monte Carlo plus-one correction, giving absolute-tail fraction $4/240 = 0.01667$. Using $\overline{D}$ with the same assignments gave $5/240 = 0.02083$. Neither construction preserves unknown clone-linked labels across KP-1/KP-2, so equal-animal weighting does not make these allocations dependence-robust. The fractions are section-level sensitivity results, not animal-level P values.

The two-programme maximum-absolute-standardized-statistic fractions were 0.02917 for the stromal programme and 0.175 for the T-cell programme under the same section-label reference. Multiplicity correction does not address its exchangeability assumptions. Pooling all mouse-4 lesions and permuting $\binom{8}{2}\binom{6}{2} = 420$ labels would require exchangeability across annotations and would ignore possible shared-clone constraints and section structure. A clone-level allocation cannot be reconstructed without a cross-section clone map.

SM8. Influence and descriptive molecular coefficients

Primary lesion endpoints, source counts and receiver-window sizes are reported in Supplementary Table 8. Deleting a singleton mutant removed its section from the allocation set, leaving 60 section-label allocations; deleting a KP-4 mutant left 80. The resulting fractions were $4/60$, $4/60$, $1/80$ and $9/80$. Because annotations can share animal and clone dependence, these section-label fractions are not animal-level significance tests.

For the descriptive molecular fits, $X_{c\ell} = 1$ identifies a mutant annotation, $M_{c\ell}$ is its mean Tgfb1/Tgfb2/Tgfb3 source score, $V_{c\ell}$ its mean near-receiver Tgfbr1/Tgfbr2/Tgfbr3 score, and $D_{c\ell}$ the fixed receiver target near–far score from the requested set (Serpine1, Ctgf, Smad7, Acta2, Tagln, Timp1, Col1a1, Col1a2, Col3a1, Fn1 and Tnc). The measured score uses the ten available symbols: Ctgf was absent under that symbol and no alias was substituted (SM7). Ordinary least squares with identity link fitted

$$M_{c\ell} = a_0 + aX_{c\ell} + u_c + \epsilon^{M}_{c\ell}, \tag{12}$$

$$D_{c\ell} = b_0 + bM_{c\ell} + c'X_{c\ell} + dV_{c\ell} + v_c + \epsilon^{D}_{c\ell}, \tag{13}$$

$$D_{c\ell} = t_0 + cX_{c\ell} + w_c + \epsilon^{T}_{c\ell}. \tag{14}$$

Section intercepts are fixed effects, with no offsets or observation weights. These fits summarize 14 annotations nested in two mice and do not assume that their residuals supply independent inferential replicates. The source Tgfbr2-expression endpoint was single-gene log-normalized expression standardized by its section mean/SD. Pooled coefficients and separate mouse fits are computed by the public `R/perturbmap.R` under the descriptive models specified here; fitting a two-cluster sandwich or mixed model would not establish reliable population precision.

The pooled ligand product is $ab = 1.556$, the matrix product 2.675 and the total receiver-target coefficient $c = 1.677$. The total model omits V, so $c = c' + ab$ need not hold. Source matrix and receiver target share Col1a1, Col1a2, Col3a1, Fn1 and Tnc; correlated mixture, overlapping receiver sets and mediator–outcome confounding remain unresolved. No mediated proportion or causal pathway is identified.

The 240 section-wise assignments refitted genotype terms while retaining measured M, V, D; the b-coefficient fraction 0.60 tested reassignment of X, not a separate null $b = 0$. The 5,000-draw section/class bootstrap resampled annotations within strata but left singleton mutant strata fixed and did not resample animals or preserve unidentified shared clones. Its percentiles, positive-draw proportion and six-mediator adjusted fractions therefore do not quantify animal-level uncertainty or mechanistic support. The coefficients are reported descriptively; these resamples are not confidence intervals or biological confirmation.

SM9. Platform-specific application and external tests

Cell-resolved data can be analysed at their original coordinates; the component grid is only a tissue-mask device. Cell-level source calling, guide dropout and segmentation error remain important. In mixed-cell spots, a detected guide or clone label assigns a capture unit, not necessarily every cell in that spot. Programme changes can reflect altered cellular abundance, cell state or both; deconvolution uncertainty should be propagated if composition estimates are used. Distances to capture centers have a resolution floor set by spot diameter and spacing, so windows cannot be copied unchanged across platforms. Library normalization, gene coverage, mask connectivity and null source clustering must be recalibrated to the measurement unit. Adjacent sections are technical/context tests unless an independent specimen design is demonstrated.

Perturb-DBiT used GSE319277 expression, guide and coordinate tables. A multi-gene expression sum served as a library-size proxy where raw whole-transcriptome library counts were unavailable. Spatial background and log_library-proxy smooths were fitted in that platform's own coordinate system. The ATG4A source programme and sign were selected solely by the direct-source contrast in Bru.LC.6 from eight fixed Hallmark choices and frozen for the Bru.LC.8 receiver test. Its 500 pseudo-source configurations retained the observed source-group sizes. The analysis used MSigDB 2026.1.Hs with msigdbr 26.1.0, retained all eight Hallmark membership lists, selected positive TGF-beta signalling in Bru.LC.6 and reproduced Bru.LC.8 at 498/501; the gene-set release used in the initial run was not recorded. The Tgfbr2 test used a fixed 11-gene target programme in two liver-tumour sections and 1,000 configurations per section, with independently generated section configurations. Averaging independent section draws by index gave a product-placement fraction of 0.322; this does not preserve unknown shared-block dependence and is not an animal-level joint test. With common centring and configuration-specific pseudo-source exclusion, the separate section values are 381/1001=0.38062 and 615/1001=0.61439 (SM13). The source Supplementary Table 1 places Bru.LC.6 and Bru.LC.8 in the same block 33-1, and mTSG.LV.2 and mTSG.LV.3 in the same block MSTGN3R. The ATG4A split is a within-block section holdout, not an animal holdout. Across the six audited sections, Brie.LU.1 maps to block 01.3.1, while Bru.LC.1.33.3 lacks an exact Table-1 match and remains unresolved; it is not substituted with the differently accessioned Bru.LC.1 record. These are stress tests in distinct tissue contexts; adjacent sections and assay units are not counted as independent animal replications.

Injury and HCC analyses used proxy sources, not genetically assigned sources. Their proximity effects establish association conditional on a proxy definition. Myocardial-infarction proxy cores were the top 5% of spots with low cardiomyocyte/high myeloid composition; patient labels, not spots, defined the injury–control inference. The primary normalization uses each spot's whole-transcriptome count denominator, published as n_counts and verified against whole-transcriptome integer count sums in all 27 sections. For gene set G, the score is the mean over available genes of $\log(1 + 10^4 C_{ig}/L_i)$, followed by the receiver-based robust scaling. Patient-level two-sided values were fibrotic 0.58803, inflammatory 0.21527, vascular 0.37296 and regenerative 0.46151 (20 patients; 4 controls; 4,845 allocations). MI coordinates were rescaled by each section's median positive nearest-neighbour spacing, assigning the nominal Visium 100-μm centre spacing per neighbour; this is an assay-based scale convention rather than independently recovered image-pixel calibration. The fixed window sensitivity is in SM13. Skin proxy cores were the top 5% inflammation-programme spots; five integrity-passing sections were analysed after two corrupted expression sections were excluded. The frozen skin score is $\log(1 + 10^4 |G|^{-1} \sum_{g \in G} C_{ig}/L_i)$, followed by the section-wide median and 1.4826-scaled MAD (SD fallback), rather than the mean of per-gene logged normalized counts. Different wounds at different days do not identify propagation speed or persistence within the same wound.

SM10. Clinical scores and descriptive reconstruction metrics

For the paired HCC cohort, $y = 1$ denotes the supplied Responder label and $y = 0$ Non-Responder (11 patients: five/six); the 22 pre/post sections are not independent units. Fixed per-sample top-2% apoptosis proxy sources are

excluded from every receiver profile, slope and near–far feature in both cohorts. The exported source-response column is excluded from all reported clinical models. Internally, direct predictors are delta-apoptosis and post-apoptosis q95 (two features), pretreatment state has four features, SPF has four receiver slopes, and combined uses the two direct plus four slopes. Externally, direct uses post-apoptosis q95 alone; SPF has four slopes and combined has five features. Five-bin curves are descriptive rather than predictors. Programme means use the fixed measured gene memberships and log1p(10,000 times gene count divided by the whole-assay count denominator). Direct delta-apoptosis, post-apoptosis q95 and pretreatment-state features use these unstandardized programme means. For receiver profiles and slopes only, the programme mean is centred/scaled by the mean and sample SD over all spots within each sample; source exclusion then applies to receiver features. Patient-level model-feature standardization is fitted separately on training patients only. The percentile q=0.98 is fixed but its absolute cutoff is sample-specific. Programmes, threshold percentile, penalty grid and validation scheme are fixed. Raw-data reconstruction reproduced the all-spot features to numerical precision; source masks and feature assignments were applied consistently across all models.

For each HCC sample and receiver programme p, the feature was the unweighted ordinary-least-squares slope β_p in

$$Z_{ip} = \alpha_p + \beta_p \log(1 + d_i) + \varepsilon_{ip}, \qquad i \in R. \tag{15}$$

Here Z_{ip} is the programme score standardized over all spots in that sample, d_i is nearest-source distance in section-normalized hexagonal spacings, and R excludes the proxy-source spots. Thus β_p is a slope per unit log-distance, not per micrometre or raw spacing.

Nested leave-one-patient-out ridge logistic regression standardized each feature on its training patients only. Its Bernoulli logit objective used an unpenalized intercept and $\lambda \sum_{j>0} \beta_j^2/2$, with $\lambda \in \{0.01, 0.1, 1, 10, 100\}$ chosen by inner leave-one-patient-out log loss. External predictions for seven CABO/NIVO samples use training means, SDs, penalty and coefficients without outcome tuning. After correcting HCC geometry to isotropic hexagonal coordinates, internal SPF-only AUC/Brier are 0/0.365232 and combined-model values 0.766667/0.206239. Direct-only values remain 0.966667/0.066379. SPF outer penalties were 100 in seven folds, 10 in three and 1 in one. A diagnostic intercept-only LOPO also has AUC 0 (Brier 0.3): held-out responders leave a 4/10 training response fraction, while held-out non-responders leave 5/10. The AUC is not post-hoc inverted. Locked external SPF and combined AUCs are 0.666667 (11/35=0.314286 one-sided) and 0.583333 (15/35=0.428571); Brier values are 0.264666 and 0.571410. Independent pixel coordinates gave the same AUCs. Clinical endpoint heterogeneity and post-treatment features remain interpretation limits.

For n_1 responders and n_0 non-responders, ROC AUC is $\sum_{i:y_i=1,j:y_j=0}[\mathbf{1}(\hat{p}_i > \hat{p}_j) + \frac{1}{2}\mathbf{1}(\hat{p}_i = \hat{p}_j)]/(n_1 n_0)$: the probability that a randomly selected responder ranks above a non-responder. The curve varies the probability threshold; individual cells or distance bins are not ROC elements. Accuracy is $n^{-1} \sum_i \mathbf{1}\{\mathbf{1}(\hat{p}_i \geq 0.5) = y_i\}$. The Brier score is $n^{-1} \sum_i (\hat{p}_i - y_i)^2$, for which smaller values indicate better probabilistic prediction. Log loss is the mean Bernoulli negative log likelihood. Exact external AUC inference enumerated assignments of the observed number of responder labels to the fixed predictions, equivalent to a one-sided exact rank/Mann–Whitney comparison conditional on those probabilities. It did not refit the model to each external outcome assignment.

The atlas/reconstruction analyses concern ordinal curve shape. Original distance labels and coordinate units are retained; HCC uses the isotropic hexagonal embedding $x = \text{array\_col}/2$, $y = \sqrt{3}\,\text{array\_row}/2$, divided by median nearest-neighbour spacing. Pixel-coordinate and threshold/window sensitivities are reported alongside the row/column index metric (SM13). B1–B5 are ordered interval labels across assays. The atlas has 62 complete curves, two descriptive groups and mean silhouette 0.48883, using the myocardial-infarction and HCC profiles. A conditional Perturb-map profile is not supported at the animal level, and the Clu evidence label indicates no support under the strict null.

RMSE summarizes score reconstruction. For ordinal energy distance, absolute deviations from the terminal bin are normalized to weights and evaluated with distances $|j - k|$ for $j, k \in \{1, \ldots, 5\}$. Active-bin agreement compares absolute deviations with one quarter of each curve's maximum deviation. Quarter-peak bin discrepancy is the absolute difference in the first post-peak index at or below that quarter threshold. Rank concordance is Spearman correlation over the five ordered bins; near-bin score error is the absolute discrepancy in the mean of B1/B2. Energy and bin discrepancies are dimensionless, not anatomical-boundary accuracy, physical radius error or vector direction.

The scalar receiver-identity predictor excludes its target identity (18 curves; mean absolute error 0.0651, median 0.0579). Perturb-map is classified as Visium. Source holdout excludes every curve from the held-out animal, including other sections. Forty annotation curves represent three animal groups, with training from one or two other animals; two Jak2 curves lack a matching other-animal class/programme and use the recorded all-other-animal fallback. Leave-programme withholds the receiver programme and is not a spatial-niche holdout. Dataset/platform/tissue-context checks

use the remaining-atlas mean and remain descriptive. Platform holdout separates two technologies: Stereo-seq and Visium. Perturb-map, myocardial infarction, skin and both HCC cohorts are all Visium; they are withheld together in a Visium fold, rather than separated by dataset or assay family. Only two technologies are represented, so this is a limited diagnostic of cross-technology profile reconstruction. These reconstruction metrics do not include a lineage map, new intervention, anatomical boundary ground truth or biological transfer validation.

Additional cohort and prediction checks. Clinical programme memberships were harmonized to genes measured in both cohorts: seven cytotoxic and five hypoxia genes, with CD8B, LDHA and BNIP3 excluded; the other three programmes are unchanged. Patient 85567 pairs a liver-primary pretreatment sample with a lymph-node-metastasis post-treatment sample. SM15 reports the metadata-defined ten-liver-pair sensitivity, same-model nested pairwise ranking, fixed training-prevalence probability baseline and a common-library-denominator sensitivity. The primary probabilities use 11 patients; these sensitivity analyses do not establish clinical utility. Tissue-context exclusion pools HCC liver and node samples and is not a single-organ holdout.

SM11. Workflow and reproducibility of the reported tests

The executable analyses are separated into guide calling/QC, non-spatial programme learning, spatial projection, receiver adjustment, source-null inference and biological-block summaries. Their order is:

1. Read expression, guide/lesion calls and calibrated coordinates; audit source/receiver exclusions and independent specimen metadata.
2. Learn source-associated weights only from the dissociated reference or training section, using mouse-paired pseudobulks; freeze gene membership and sign before the receiver test.
3. Project the fixed programme; define receiver identity and within-chip score normalization. Fix distance windows and the tissue mask for the stated analysis.
4. For strict Clu, fit the receiver-only background model once and retain its residuals. Compute observed near–far contrasts and coverage.
5. Generate source-count or cluster-size-matched pseudo-sources within components; recompute nearest distances and contrasts with the fixed receiver scores. Record valid configurations and use the stated centered or uncentered Monte Carlo formula.
6. Summarize effects by chip, retain multiplicity-family labels and report non-estimable cases. For lesions, retain annotation/section/animal nesting, report animal-level descriptive contrasts and verify clone-level exchangeability before any label randomization. Section-label enumeration does not provide animal-level inference when cross-section clone identity is unresolved.
7. Report specimen/section/lesion counts separately from descriptive cell/spot counts. Analyse sensitivities and external contexts without treating them as new biological replication.

Programme construction, matching, source/identity coverage and lesion-level results are reported in Supplementary Tables 2–9; dataset coverage and aggregate animal summaries are in Supplementary Tables 10–11. The corresponding public code modules and input boundaries are identified below. Source-count and strict-cluster comparisons change several controls simultaneously in the archived data; their difference is not, by itself, an isolated estimate of the effect of clustering. Likewise the original synthetic estimator demonstration was not a repeated type-I-error or power experiment and cannot establish P-value calibration. Any new calibration benchmark is reported separately with its own simulation design and output provenance.

The public repository and fixed version are identified in Code availability. Its `DATA_DICTIONARY.tsv` and `DATA_SOURCES.tsv` describe the compact objects and their provenance. The numerical implementation uses `run.R` with modules `R/reference.R`, `clu.R`, `perturbmap.R`, `dbit.R`, `clinical.R`, `atlas.R` and `profiles.R`; the repeated generators are `benchmark.R` and `injection.R`. `R/spf_core.R` supplies reusable component-aware operations, nuisance models and source references. Optional raw-data interfaces require the original public matrices; `optional/apply.R` documents a new-data receiver/source interface. Compact data have an object-level dictionary and provenance. The original reference export uses the joint five-mouse representation; the additional raw foldwise pipeline and its compact Clu/mSafe refit are separate, explicitly labelled interfaces (SM13). Feature-level clinical/injury reruns are distinguished from raw extraction. The complete 14-scenario, 100-realization synthetic generator was rerun after the common-centre/source-exclusion corrections, and its fresh records are the bundled simulation input. Additional

executable modules include foldwise reference validation, guide-count/receiver-error simulation, native generated-case comparison, rigid-footprint diagnostics and fixed threshold/window sensitivities (SM13). The repository separates compact inputs from original public matrices. Dependency records are in `DEPENDENCIES.tsv`, with commands in `run.R`, the README and the optional adapters; no automatic download or installation occurs during an analysis.

The real-source assignment and guide-selected receiver/candidate sets do not satisfy the known-law rank premises demonstrated in SM14. BH-adjusted spatial-reference fractions are descriptive scores and do not establish false-discovery-rate control; the original edgeR gene-testing specification is separate.

Descriptive lesion geometry and composition sensitivity

A covariate sensitivity examined lesion size, tissue-edge proximity and receiver composition alongside the frozen stromal near–far endpoint for 14 lesions in the three informative sections. The three KP lesions in KP-3 were excluded because that section had no focal lesion. Source size was represented by $\log(1 + n_\ell)$, where n_ℓ is the number of labelled source spots. Pixel coordinates were converted using each section's published spot-diameter scale factor (55 µm capture diameter). An observed-mask boundary spot was defined by fewer than six neighbours within 1.25 times the median nearest-neighbour spacing; edge proximity was the mean distance from a lesion's source spots to the nearest such boundary spot. This is an edge of the observed capture mask, not a manually segmented histological boundary. The composition proxy was the fraction of eligible receiver spots within 200 µm labelled "normal" rather than "tumor" by the publisher's coarse k-means annotation. It is not an estimate of fibroblast, immune or tumour-cell fractions.

For fixed lesion endpoint A_ℓ, genotype indicator G_ℓ, source-size term V_ℓ, mask-edge distance D_ℓ and composition proxy Q_ℓ, the exploratory Gaussian identity-link model was

$$A_\ell = \alpha_{s(\ell)} + \beta G_\ell + \gamma_V V_\ell + \gamma_D D_\ell + \gamma_Q Q_\ell + \epsilon_\ell. \tag{16}$$

Unweighted ordinary least squares used fixed section intercepts, no offsets and no interactions. Nested sensitivities omitted the covariates listed below. The genotype coefficient was recomputed for all 240 within-section assignments; the two-sided label-reference P value was $240^{-1} \sum_b \mathbf{1}\{|\beta^{(b)}| \geq |\beta^{obs}|\}$. Independent section-label allocation does not retain unknown cross-section clone labels, so these fractions are not animal-level P values. Because this statistic pools lesions through a common regression coefficient, its section-only value (1.3054) differs from the section-weighted statistic (1.2078).

Covariates in addition to section	Genotype coefficient	Section-label fraction
None	1.3054	0.02083
Source size	1.3790	0.03750
Observed-mask edge distance	1.3507	0.02917
Normal/tumour composition proxy	1.1830	0.04583
Source size and mask-edge distance	1.5988	0.02500
Source size, mask-edge and composition proxy	1.5202	0.05833

The pooled coefficient is positive, but section-label fractions do not provide animal-level significance tests. The fully adjusted coefficient changes sign across separate animal fits: -0.9556 in mouse 4 and 1.0034 in mouse 7 (eight and six annotations, respectively). These small fits are descriptive and provide no reliable standard errors or population inference; the adjusted direction is not stable across animals. Fourteen lesions do not provide enough independent source contexts to establish a stable, fully composition-adjusted genotype effect. Source size and receiver composition may themselves be consequences of perturbation; conditioning on them changes the estimand and can remove part of a genuine total effect. Regression and enumeration do not establish conditional exchangeability of genotypes. Residual confounding and a biological contribution to the unadjusted association both remain plausible.

Applying the same random-source null to lesions and single cells would require a lesion-mask placement model and common spatial support. The observed lesion masks differ in size, shape and location, so random scattering of source spots would not preserve their geometry. Perturb-map is evaluated using real KP annotations and covariate diagnostics. Complete-footprint transport and location/composition matching had insufficient legal-placement support for the original animal estimand. Real-KP matching and coarse composition standardization are also reported as descriptive sensitivities (SM13). Spatial Perturb-Seq lacks real matched KP-like lesions. The two primary designs therefore have complementary controls, and their nulls are not empirically interchangeable.

The executable analysis is `R/perturbmap.R` (`Rscript run.R perturbmap`). The six pooled results above are descriptive and are not interpreted as animal-level significance evidence. The public command writes per-lesion covariates to `focal_geometry_covariates.tsv` and descriptive pooled and animal-specific coefficients to `geometry_coefficients_descriptive.tsv` within its `perturbmap/` output directory. The spot-level count input is `data/perturbmap_spots.tsv.gz` in the public repository.

Supplementary Results

Perturb-map stromal contrasts were positive in both sampled animals across 64 specifications, but remain descriptive because repeated sections and unidentified cross-section clones invalidate an unqualified independent section-label reference. The old section-label fractions and bootstrap outputs are retained only for audit. Animal-specific adjusted coefficients also reveal instability. External Perturb-DBiT sections share tissue blocks and do not provide independent animal confirmation. Injury and clinical analyses were recomputed after correcting myocardial-infarction normalization and excluding HCC proxy sources from receiver features; their revised results and ordinal profile-reconstruction checks are reported below.

The analysis includes independently preprocessed mouse folds, a repeated guide-count/receiver-contamination model, native software on shared generated data, rigid-footprint support diagnostics and coordinate/window sensitivities (SM13). Results include negative estimates and settings with inadequate support. Independent preprocessing does not establish stable programme transfer, and the spatial-source reference fails calibration in specified generative regimes. These analyses do not add Perturb-map animals.

Supplementary Table 1
Design-dependent scope of a spatial field test. Counts of cells or source clusters are not substitutes for biological replication. Minimum coverage is a design condition rather than a universal numeric cutoff.

Design	Source and receiver	Replicate, control and null	Identifiability and minimum coverage
Pooled multi-guide screen	Single-guide cells; guide-undetected non-source receivers	Animal/specimen; control guides and source-niche coverage; component/count and joint geometry-aware nulls	Several specimens and source clusters, with overlap of source niches and receiver identities. A guide confined to one specimen or niche supports only a conditional local association. Marginal guide tests do not separate overlapping exposures.
Repeated single intervention	Assigned intervention cells/regions; non-source cells	Independent animals, sham interventions and matched locations; specimen-level or restricted location null	Replication of the intervention, not repeated cells/sections. Intervention and location must not be inseparable. Supports a local effect within the sampled context.
Clone/lesion design	Assigned clone footprint; non-lesion/peripheral measurements	Animal/lesion nested in specimen; control lesions in the same context; traceable clone/animal blocks before label reallocation, plus geometry/composition controls	Both classes within informative blocks, enough lesions to separate class from size/location. The present four-mutant design has only two contributing mice under the GEO-based grouping and cannot establish broad animal-level generality.
Localized non-genetic intervention	Known injection, ablation or delivery footprint; eligible exterior cells	Independent specimen; sham footprint and repeated randomized locations; footprint-preserving null	Requires edge, injury and delivery controls. Spatial gradients do not separately identify signalling, damage and cell recruitment without extra measurements.
Proxy-source dataset	Core defined by injury, composition or expression; remaining spots	Patient or independent wound; matched control context and patient labels	Source is not randomized and may share genes with the endpoint. Supports proximity association and transfer stress tests, not a genetic perturbation effect or a transmission mechanism.

Supplementary Table 2
Reconstructed Clu nearest-state matching diagnostics. The original processed reference object and the exact input-order, no-replacement 50-nearest-candidate matching algorithm were used. Broad identity matched for every Clu/control pair. Distances are in the fitted Harmony coordinate system, without a universal biological cutoff. SMD denotes target-minus-control standardized mean difference.

Mouse	Target/control	Median d	95th d	Identity	Library SMD	Feature SMD
B1-1	9/9	4.216	8.381	1.00	−0.172	−0.215
B1-2	11/11	3.042	6.047	1.00	0.148	0.125
B2-1	20/20	3.527	4.659	1.00	−0.093	−0.135
B2-2	8/8	3.933	7.627	1.00	0.217	0.243
B3-1	30/30	4.307	6.647	1.00	−0.013	−0.016

These diagnostics do not constitute a held-out-mouse test of programme separation. The public input `data/reference_counts.rds` retains the exact matching pairs and their distance, identity, library and feature-count fields. The raw-reference adapter reconstructs the joint-reference matching; the training-only folds are a separate analysis (SM13).

Supplementary Table 3
Conditional Clu mouse-holdout weight-learning diagnostic. All fits exclude the held-out mouse from Clu and mSafe differential expression, but retain the frozen five-mouse Harmony representation. AUC compares Clu-guide and equally numerous matched guide-undetected cells in the held-out mouse. Effect is the mean target–control score difference divided by held-out-control MAD. These are descriptive within-mouse diagnostics, not propagation tests; independent-preprocessing folds are reported in SM13 and Table 14.

Held out	Programme	Target/control	Genes	AUC	Effect	Status
B1-1	All retained	9/9	11	0.457	0.059	Estimable
B1-1	No IEG	9/9	8	0.426	−0.349	Estimable
B1-2	Both variants	11/11	–	–	–	mSafe < 3 mice
B2-1	Both variants	20/20	–	–	–	mSafe < 3 mice
B2-2	All retained	8/8	22	0.391	−0.323	Estimable
B2-2	No IEG	8/8	19	0.391	−0.386	Estimable
B3-1	Both variants	30/30	–	–	–	mSafe < 3 mice

IEG exclusion removes Fos, Fosb and Junb. In three folds the training-only mSafe comparison has only two eligible mice, below the three-mouse rule; programme construction is unestimable. These folds are reported as unavailable and excluded from inference. Metrics for all folds are retained as `heldout_metrics` in the public input `data/endpoints.rds`.

Supplementary Table 4
Primary spatial platforms and resulting analysis choices. Resolution and assay unit determine what can be called a source or receiver.

Platform/data	Measurement and source	Receiver and distance	Inference and limitation
Spatial Perturb-Seq, GSE274447	229,775 cell bins, three chips; positive guide feature, exactly one guide	No detected guide; original cell coordinates; 0.5 μm per native unit; within-component nearest-source distance	Chip-wise count or cluster-size-matched source null. Segmentation, dropout and identity-transfer errors remain. Published chip groups span mice 1; 2/3; and 4. Chip 2 cannot be split into animal-level effects.
Perturb-map, GSE193460	6,363 Visium spots, four sections; source clone annotation from the study	No recognized lesion class, including periphery; 55-μm capture diameter calibration; nearest labelled source-spot center	Four mutant versus ten KP lesions in three informative sections from two mice; Animal summaries; historical 240 section-label assignments do not control unidentified shared clones. Mixed-cell receivers and lesion size/location are not fully controlled.
Perturb-DBiT, GSE319277	Expression/guide capture units in training/held-out lung and two liver-tumour sections	Guide-undetected capture units; assay-coordinate clustering and platform-specific distance windows	Frozen programme tests, 500 or 1,000 source configurations. Adjacent sections and distinct tumour contexts are stress tests, not new independent animal replications.

Supplementary Table 5
Nearest-state programme support in the five-mouse dissociated reference. Cells are the summed target cells entering retained mouse pairs; an equal number of unique guide-undetected controls entered the paired pseudobulks. Mouse counts below three were excluded. Gene counts follow the FDR/effect filter and nine-gene mSafe subtraction. These training summaries do not measure held-out discrimination.

Programme	Genes	Up/down	Mice	Target/control cells	Target counts by reference mouse
Cfap410	1	0/1	4	29/29	4, 1, 6, 4, 15
Clu	12	12/0	5	78/78	9, 11, 20, 8, 30
Dpp6	1	1/0	5	40/40	7, 7, 10, 3, 13
Fasn	15	8/7	5	49/49	8, 6, 8, 3, 24
Flcn	6	6/0	5	45/45	5, 7, 7, 4, 22
Gfap	1	1/0	5	53/53	7, 9, 7, 3, 27
Rbfox	3	1/2	5	46/46	11, 5, 3, 3, 24
Rraga	8	1/7	5	59/59	5, 5, 10, 7, 32
Stk39	1	1/0	5	65/65	8, 8, 16, 5, 28
Tbk1	11	10/1	3	23/23	4, 2, 7, 2, 12
Trem2	2	0/2	4	37/37	4, 9, 10, 2, 14

Mouse order: B1-1, B1-2, B2-1, B2-2, B3-1. Dpp6 has no identically named spatial guide feature; the spatial label dpp5 was not reassigned. Clu immediate-early-gene exclusion leaves nine genes. Programme summaries and guide counts are retained as `reference_programme_summary` and `reference_guide_audit` in the public input `data/endpoints.rds`.

Supplementary Table 6
Spatial source coverage by guide and chip. Each entry gives single-guide sources / source clusters / source-bearing tissue components. Clusters use the source-only 100-μm graph; their number is geometric coverage, not a biological replicate count. Bold guides have a learned programme with matching spatial name. Cell-level guide and transferred identity labels are retained in the public input `data/clu_cells.rds`.

Spatial feature	C02943C3	B03018A2	A03599E2	Programme/coverage status
c9orf72	3/3/2	51/46/2	6/6/2	No primary learned programme
**cfap410**	8/7/2	118/97/2	13/13/2	Three evaluable chips
**clu**	29/20/2	419/241/2	60/42/2	Three evaluable chips
dpp5	2/2/2	74/67/2	12/12/2	Does not match Dpp6
**fasn**	24/21/2	413/243/2	19/19/2	Three evaluable chips
**flcn**	4/4/1	178/146/2	12/12/2	Two evaluable chips
**gfap**	9/7/2	161/119/2	15/12/2	Three evaluable chips
lrrk2	4/4/1	43/39/2	6/6/2	No primary learned programme
msafe	7/7/2	96/81/2	9/9/2	Calibration control
ndufaf	4/4/2	116/96/2	9/9/2	No primary learned programme
oligo2	19/16/2	357/218/2	28/26/2	No primary learned programme
**rbfox**	4/4/2	100/90/2	7/7/2	Two evaluable chips
**rraga**	9/9/2	147/131/2	9/8/2	Three evaluable chips
sh3gl2	4/4/1	197/150/2	27/25/2	No primary learned programme
srf	2/2/1	72/62/2	9/8/2	No primary learned programme
**stk39**	22/19/2	351/220/2	27/27/2	Three evaluable chips
**tbk1**	3/3/2	82/76/2	16/13/2	Two evaluable chips
**trem2**	8/8/2	228/157/2	17/16/2	Three evaluable chips
All single-guide	165	3,203	301	Exact one-feature calls
Multiple-guide	11	1,054	21	Excluded from sources/receivers
Guide-undetected	39,686	119,664	65,670	Receiver eligibility
Guide-positive fraction	0.442%	3.435%	0.488%	Detection fraction, not sensitivity

Counts summarize the cell-level coordinates and guide labels retained in the public input `data/clu_cells.rds`. Source-bearing components are coarse tissue pieces; anatomical niches and independent source lineages are not identified by this count.

Supplementary Table 7
Clu source and receiver identity coverage. Spatial labels are transferred broad categories, not independent experimental cell-type assignments. The source labels are not constrained in the pseudo-source sampler. Reference counts show the source-composition imbalance in programme learning.

	C02943C3		B03018A2		A03599E2		Reference
Identity	Source	Receiver	Source	Receiver	Source	Receiver	Clu cells
Astrocyte	13	7,433	102	21,986	17	12,682	11
Endothelial	2	5,785	61	19,396	13	8,056	2
Microglia	6	6,517	87	17,724	11	12,585	4
Oligodendrocyte	0	7,971	34	22,272	10	11,013	60
OPC	5	6,772	74	21,503	7	11,125	0
Perivascular	3	5,208	61	16,783	2	10,209	0
Excitatory neuron	–	–	–	–	–	–	1

The reference contains a different cell-type mixture from the spatial assay. A dash denotes an absent spatial reference category, rather than a validated absence of that cell type from tissue. SM2–SM3 specify programme eligibility and identity transfer; the public raw-reference adapter reconstructs per-cell reference labels from the original matrices.

Supplementary Table 8
Lesion-level primary stromal effects in the three informative Perturb-map sections. Annotations are dependent subsamples nested in two animals (KP-1/KP-2 share mouse 4; KP-4 is mouse 7). The effect is the section-standardized receiver score at 0–200 μm minus 400–800 μm. Receiver counts can overlap between lesions and are descriptive. KP-3 has no mutant lesion and is not part of the class comparison.

Section	Lesion	Class	Source spots	Near	Far	Effect
KP-1	Tgfbr2-1	Mutant	75	74	245	1.691
KP-1	KP-1-1	KP	110	419	474	0.890
KP-1	KP-1-2	KP	75	332	479	−0.036
KP-1	KP-1-3	KP	48	93	394	0.302
KP-2	Tgfbr2-2	Mutant	62	88	471	0.637
KP-2	KP-2-1	KP	148	434	408	−0.350
KP-2	KP-2-2	KP	97	453	385	0.238
KP-2	KP-2-3	KP	35	116	298	0.115
KP-4	Tgfbr2-4-1	Mutant	50	39	105	3.736
KP-4	Tgfbr2-4-2	Mutant	37	51	273	0.409
KP-4	KP-4-1	KP	115	306	269	0.749
KP-4	KP-4-2	KP	74	69	134	0.559
KP-4	KP-4-3	KP	38	99	234	−0.597
KP-4	KP-4-4	KP	35	67	204	0.851

Within-section mutant-minus-KP contrasts are 1.305, 0.636 and 1.682, giving the equal-section mean 1.208. The equal-animal summary is 1.3263 (mouse 4: 0.9707; mouse 7: 1.6820). The four mutant annotations are not verified independent clones and their effects are heterogeneous; the high Tgfbr2-4-1 value is explicitly retained in the influence analysis.

Supplementary Table 9
Section-label fractions after annotation deletion. No random draws were used, but independent section-label allocation does not preserve unknown shared-clone labels across sections and the fractions are not animal-level P values. The statistic changes to an equal-two-section mean when the deleted annotation was the only mutant in its section.

Omitted lesion	Sections	Mutants	Assignments	Effect	Historical fraction	Numerator/denominator
None	3	4	240	1.208	0.0167	4/240
Tgfbr2-1	2	3	60	1.159	0.0667	4/60
Tgfbr2-2	2	3	60	1.494	0.0667	4/60
Tgfbr2-4-2	3	3	80	1.762	0.0125	1/80
Tgfbr2-4-1	3	3	80	0.653	0.1125	9/80

Supplementary Table 10
Dataset coverage used in the archived analysis. Cells and spots describe coverage, not independent replicates. Spatial Perturb-Seq has three chip blocks spanning four published mice, with two mice in chip 2; its physical section total is unresolved because the source table and figure legend differ. Perturb-map has four sections from three mice, with only two mice contributing to the focal comparison. These heterogeneous units are not summed into a cross-study biological replicate count.

Dataset	Biological/analysis units	Sections/chips	Cells/spots	Source–region pairs
Spatial Perturb-Seq	4 mice / 3 chips	3 chips	229,775	45
Perturb-map	3 mice (2 focal)	4 sections	6,363	20
Human myocardial infarction	20 patients	27	88,704	27
Paired HCC immunotherapy	11 patients	22	104,418	11
CABO/NIVO HCC	7 samples	7	17,292	7
Skin-wound time course	5 sections	5	3,885	5

Supplementary Table 11

Animal summaries and external conditional diagnostics. Perturb-map has only two contributing animals; its effects are descriptive, without an animal-population P value. The whole-animal sign-flip resolution check is 0.50 only under its explicit symmetry assumption. External liver sections share block MSTGN3R; their separate source-placement references are not independent animal replications.

Comparison	Effect	Reference	Interpretation
Perturb-map stromal, animal mean	1.3263	2 mice	Descriptive
Perturb-map T-cell, animal mean	-0.1477	2 mice	Descriptive
Perturb-DBiT ATG4A section	0.0006	0.994	Conditional; not supported
Perturb-DBiT mTSG.LV.2	0.0597	0.381	Conditional; not supported
Perturb-DBiT mTSG.LV.3	0.0348	0.614	Conditional; not supported

Supplementary Table 12

Descriptive ordinal reconstruction summaries. RFE is score RMSE; ordinal energy and quarter-peak errors use indices 1–5 and are dimensionless. Active-bin agreement is not anatomical boundary accuracy. Perturb-map training excludes the entire held-out mouse, including repeated sections; 40 annotation curves represent three mouse groups. These are curve checks, not type-I error, power or biological generalization (SM10).

Validation	RFE	Ordinal energy	Active bins	Quarter-peak bins	Rank	Near-bin error	Curves
Leave-chip	0.285	0.449	0.793	0.889	0.344	0.280	27
Leave-gene/perturbation (SPS)	0.321	0.396	0.837	0.852	0.011	0.323	27
Leave-Perturb-map-animal (PM)	0.285	0.309	0.845	0.650	0.588	0.275	40
Leave-programme (PM)	0.405	0.348	0.880	0.500	0.620	0.471	40
Leave-dataset	0.331	0.252	0.894	0.565	0.505	0.301	62
Leave-platform	0.354	0.260	0.894	0.565	0.505	0.300	62
Leave-tissue-context	0.332	0.252	0.894	0.565	0.505	0.303	62

Supplementary Figures

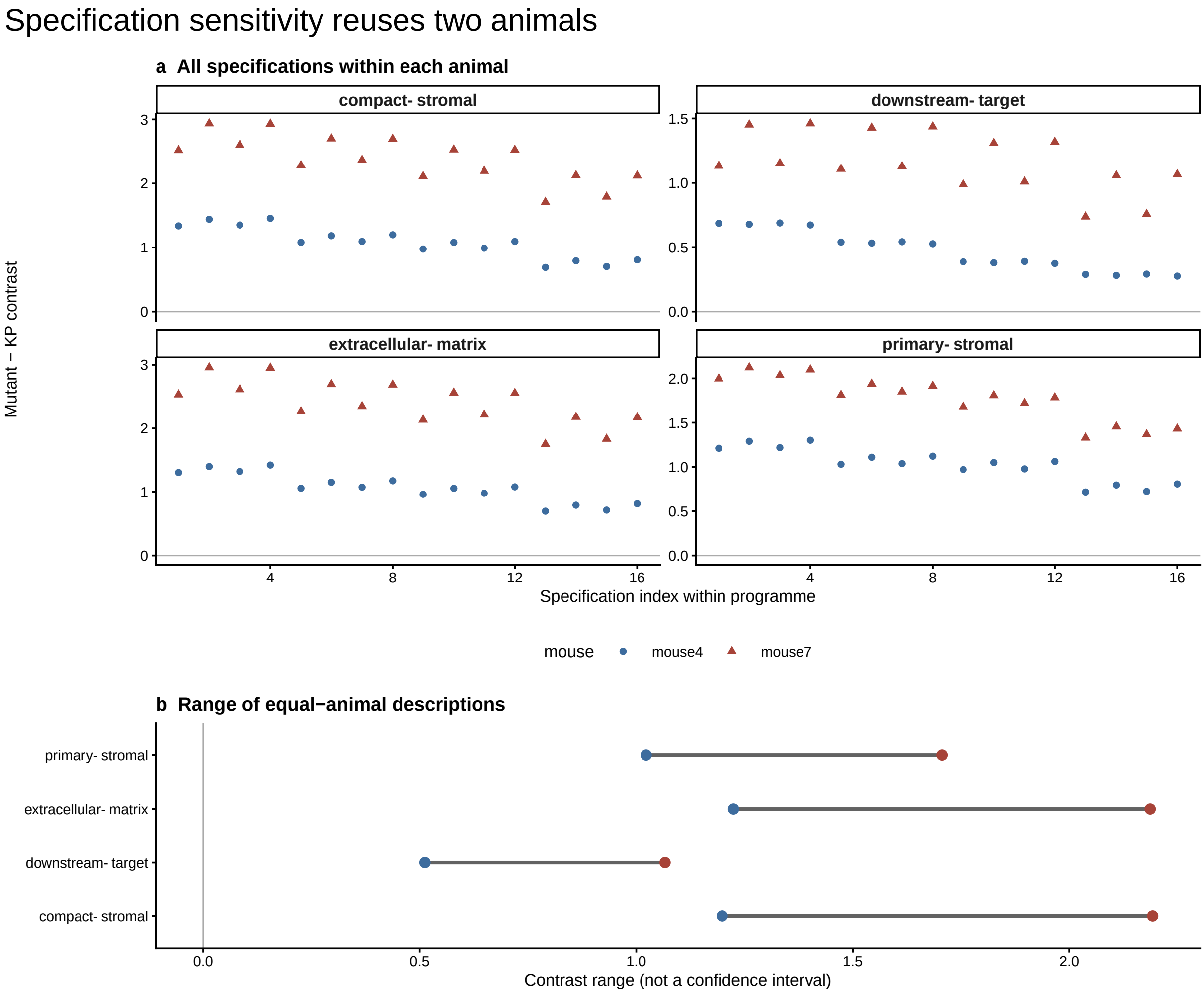

Supplementary Fig. 1: Animal-specific descriptive sensitivity of the stromal contrast. a, Contrasts in each of the two animals across all 64 programme/distance specifications. **b**, Equal-animal effect range for each programme. No specification adds an animal and no lesion-level significance threshold is shown. KP-1/KP-2 share mouse 4; KP-4 is mouse 7. Section-label deletion/reference fractions are reported in SM7–SM8.

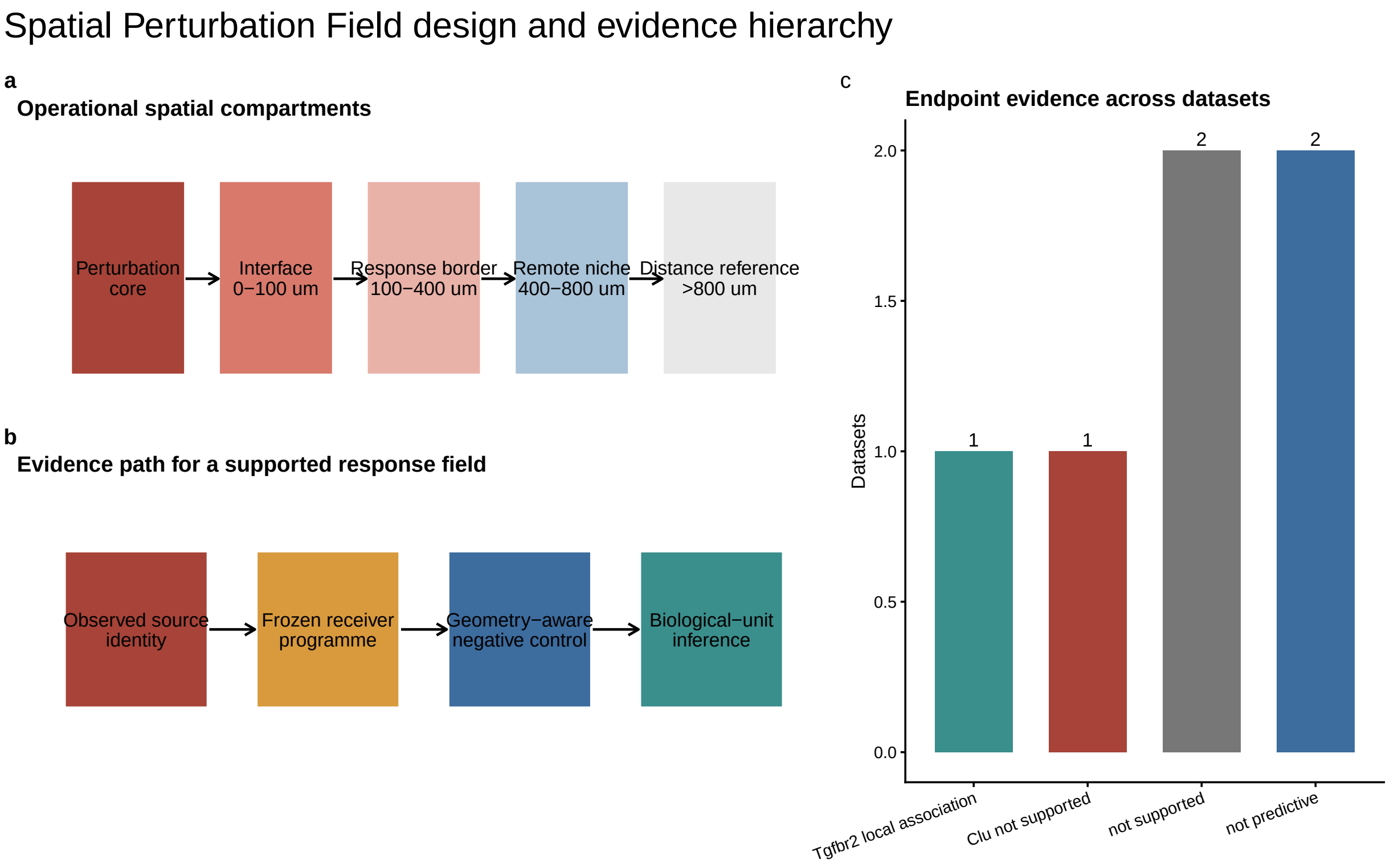

Supplementary Fig. 2: Operational field compartments and evidence hierarchy. The schematic separates perturbation core, interface, response border, remote niche and distance-defined background, and summarizes the evidence requirements used across datasets.

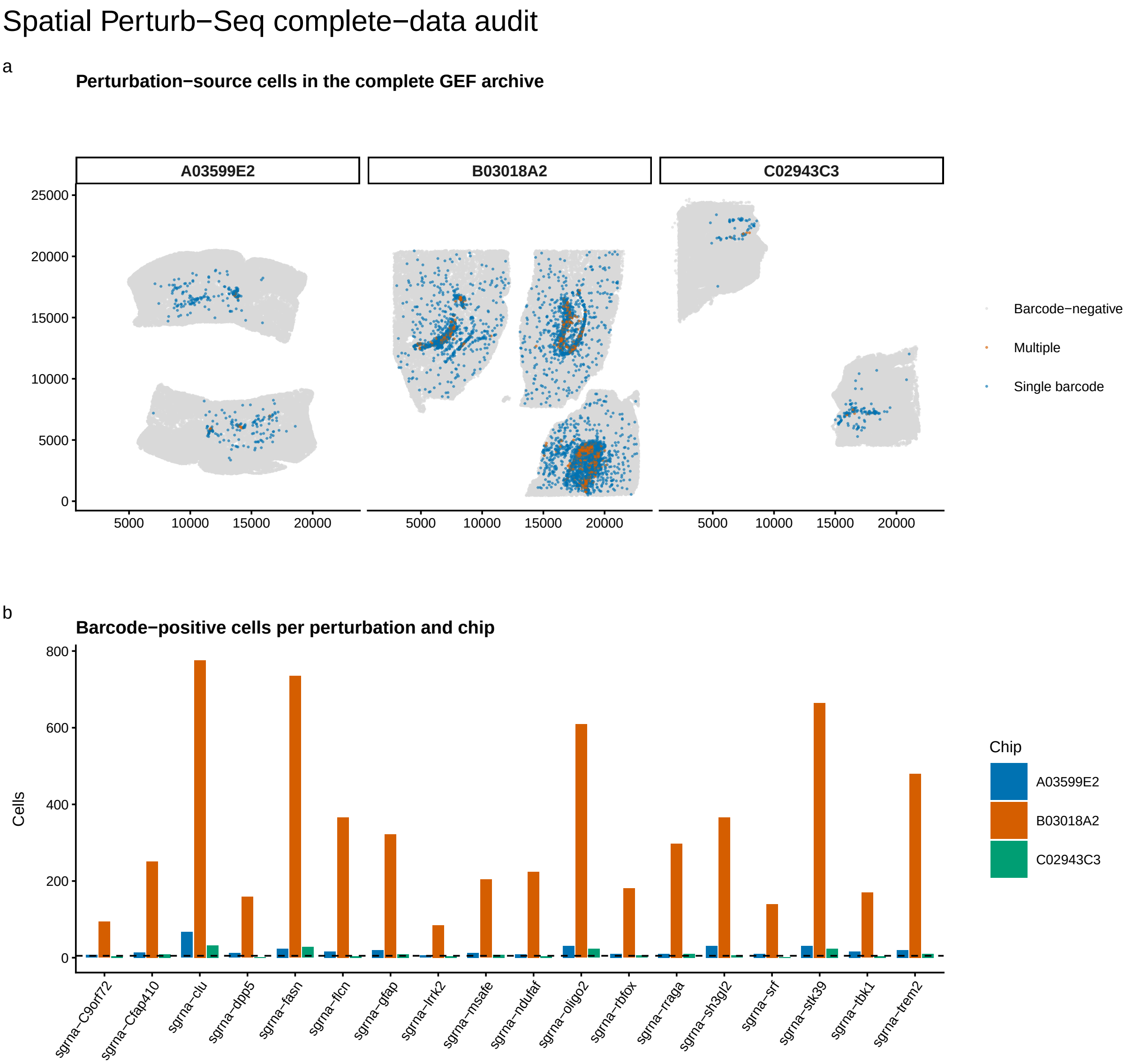

Supplementary Fig. 3: Spatial Perturb-Seq guide-call audit. Tissue coordinates, single-guide eligible sources, multiple-guide ambiguous cells and guide coverage are summarized across the three analysed chips (229,775 cells). The three chip blocks span four published mice: C02943C3, mouse 1; B03018A2, mice 2/3; A03599E2, mouse 4. The two animals in the middle chip cannot be separated using the available cell metadata.

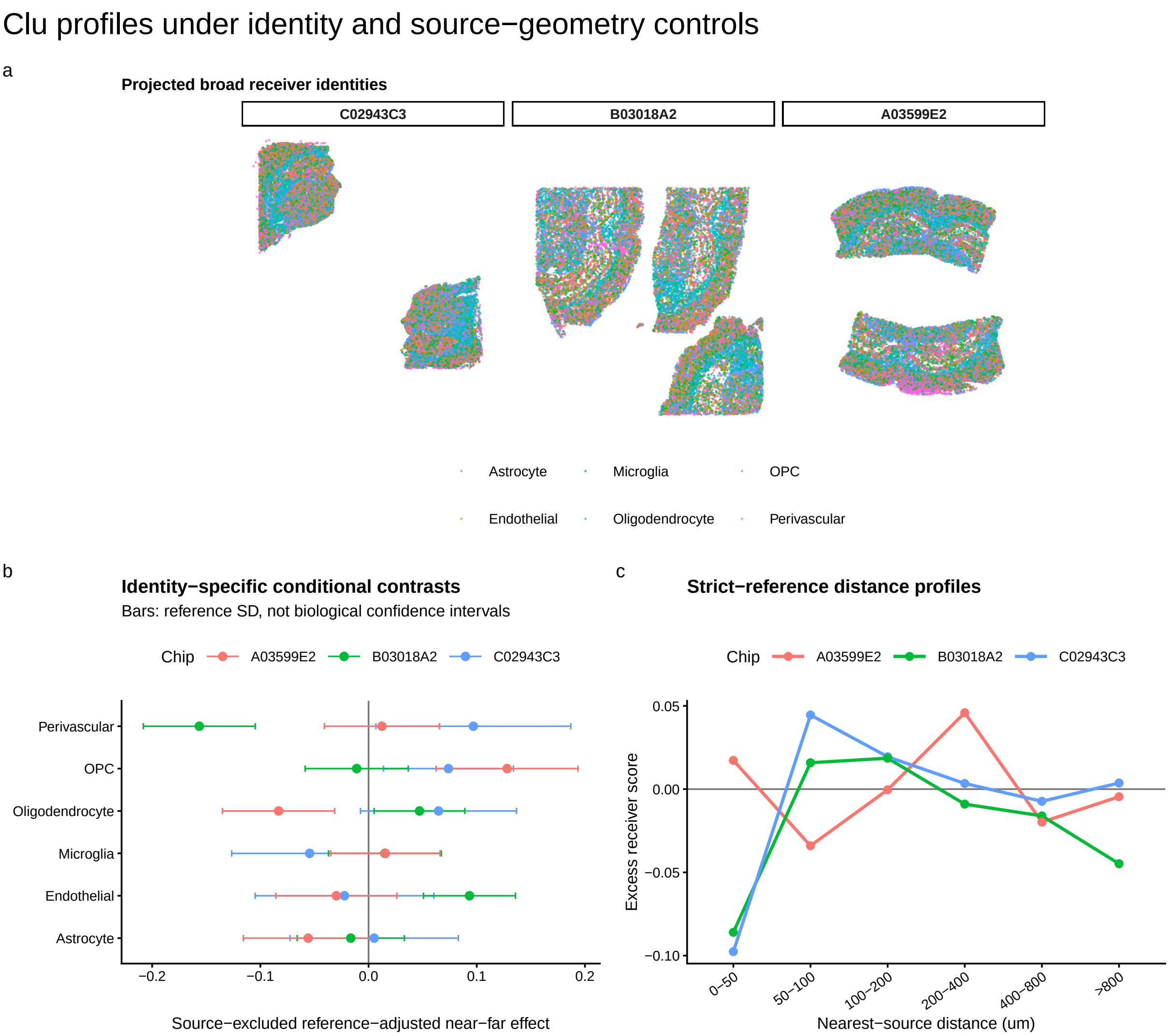

Supplementary Fig. 4: Receiver identity and spatial geometry for the *Clu* candidate. Broad receiver identities, identity-specific effects and strict-reference distance profiles are shown in three chips. There are 29, 419 and 60 Clu sources, with 20, 241 and 42 source clusters. Cell counts describe coverage; chip-wise references use 99 pseudo-source configurations and seven-endpoint BH families, with common observed/null centring and pseudo-source receiver exclusion (SM5). Reference SD bars are not biological confidence intervals.

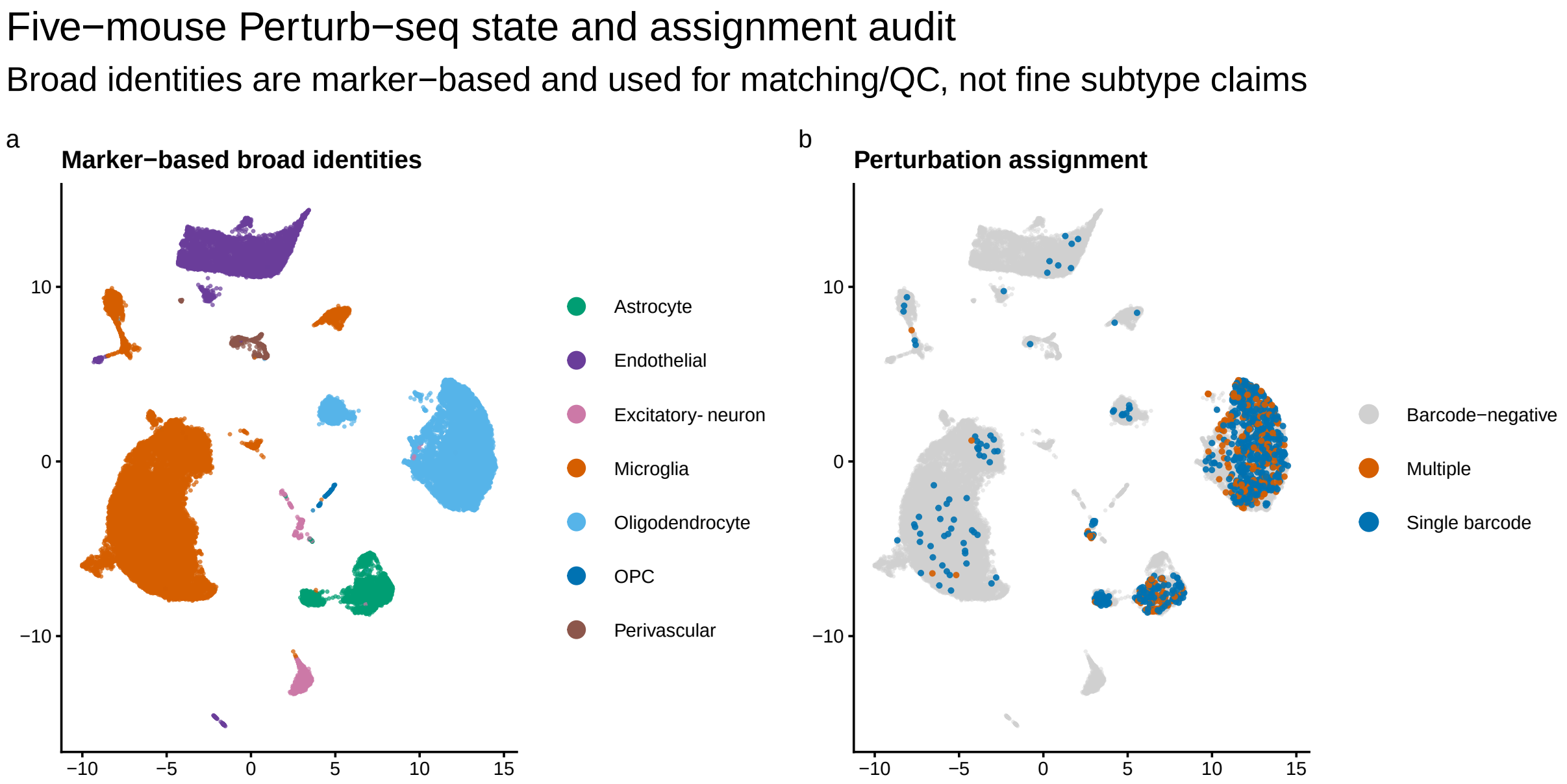

Supplementary Fig. 5: Matched mouse Perturb-seq programme-learning audit. Five-mouse guide coverage, marker-based broad identity and programme-learning context. The reference contains 41,140 QC-passing cells; programme counts and matched pseudobulk support are reported in the programme table. This panel is a training/QC audit and does not show independent held-out mouse discrimination.

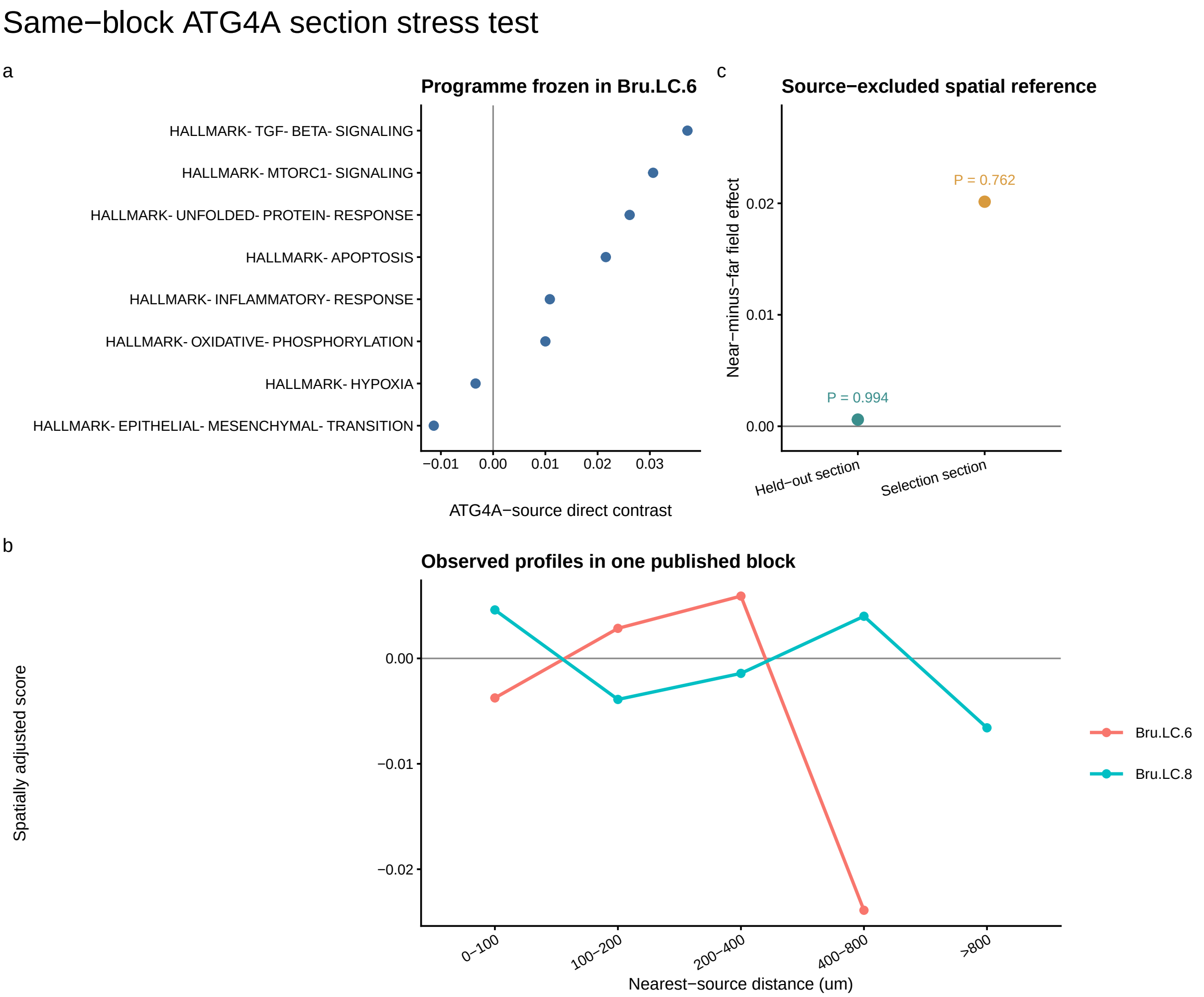

Supplementary Fig. 6: Held-out Perturb-DBiT *ATG4A* stress test. Programme selection was restricted to Bru.LC.6 and receiver-field inference to Bru.LC.8 under a cluster-size-matched source null with 500 configurations. Both sections are assigned to the same published block 33-1 and do not constitute independent animal replication.

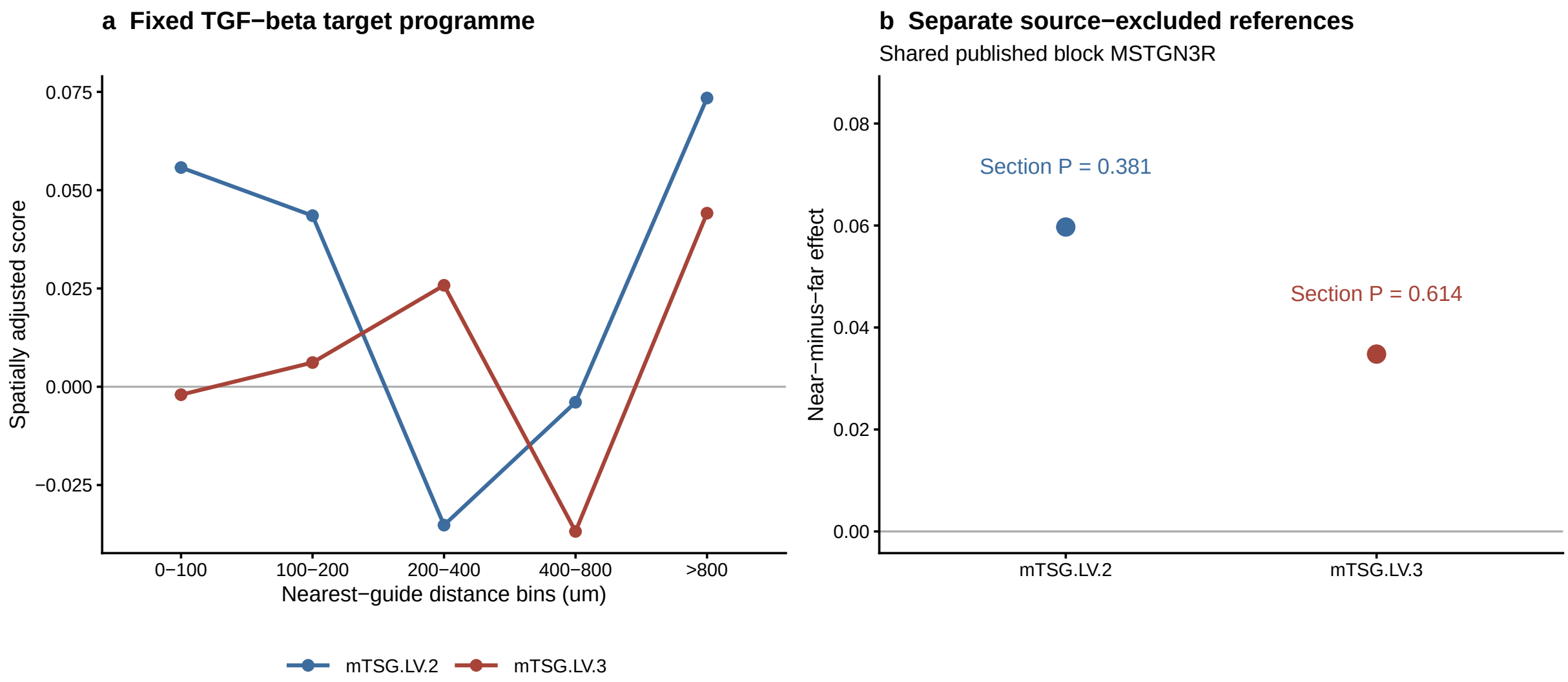

Supplementary Fig. 7: Perturb-DBiT *Tgfbr2* cross-context test. Two liver-tumour sections showed small positive observed effects with separate conditional values 0.381 and 0.614, based on 1,000 source configurations per section and the corrected centre/receiver exclusions. The independently generated product-placement reference is not an animal-dependence-preserving joint test and is not used for that claim. Both sections are assigned to the same published block MSTGN3R; they test cross-context assay compatibility, not independent animals.

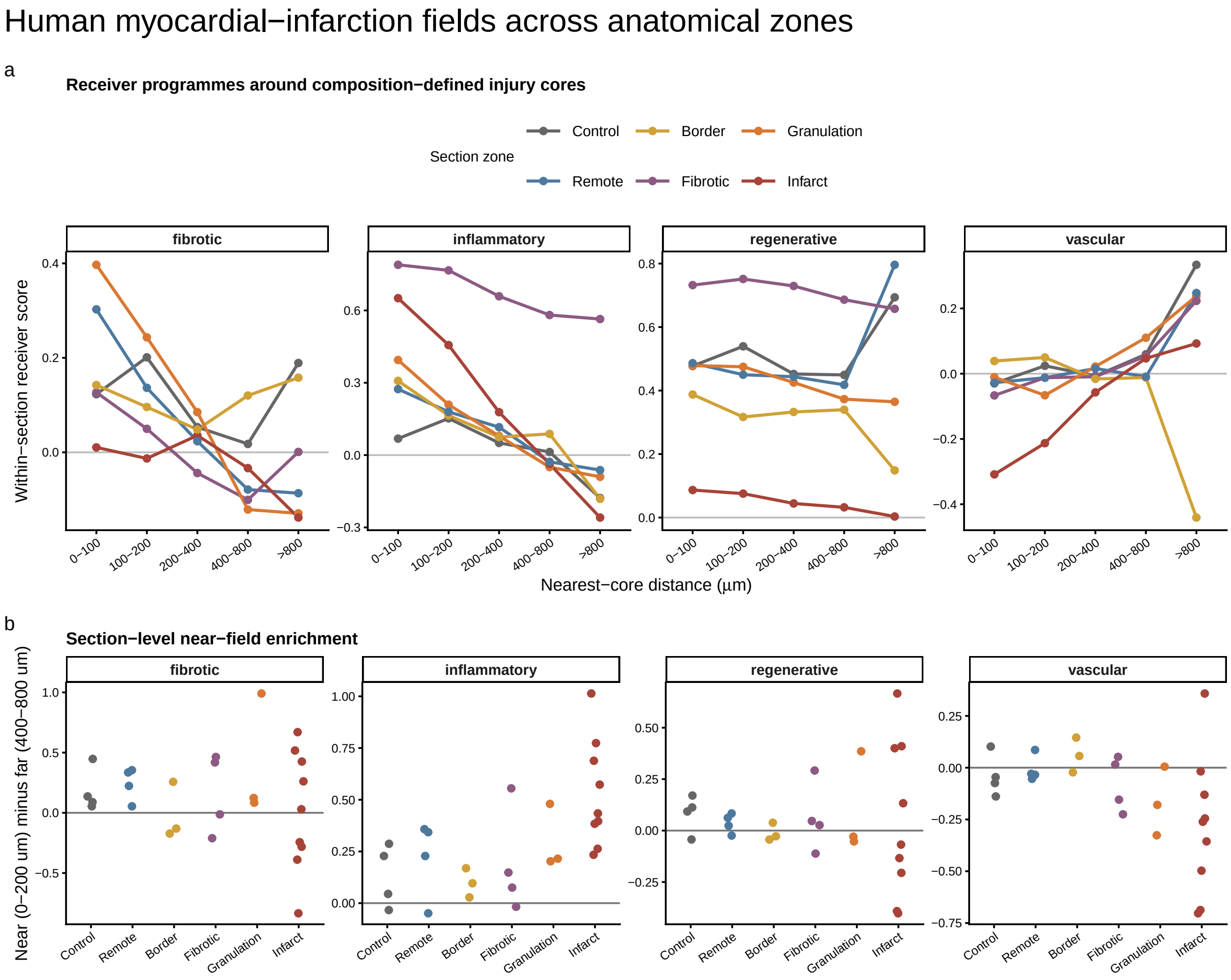

Supplementary Fig. 8: Myocardial-infarction proxy fields. Scores use the corrected whole-transcriptome count denominator in all 27 sections. Exact patient-label comparisons in 20 patients (four controls; 4,845 allocations) gave fibrotic 0.5880, inflammatory 0.2153, vascular 0.3730 and regenerative 0.4615. Spots and sections within a patient do not supply independent patient replicates.

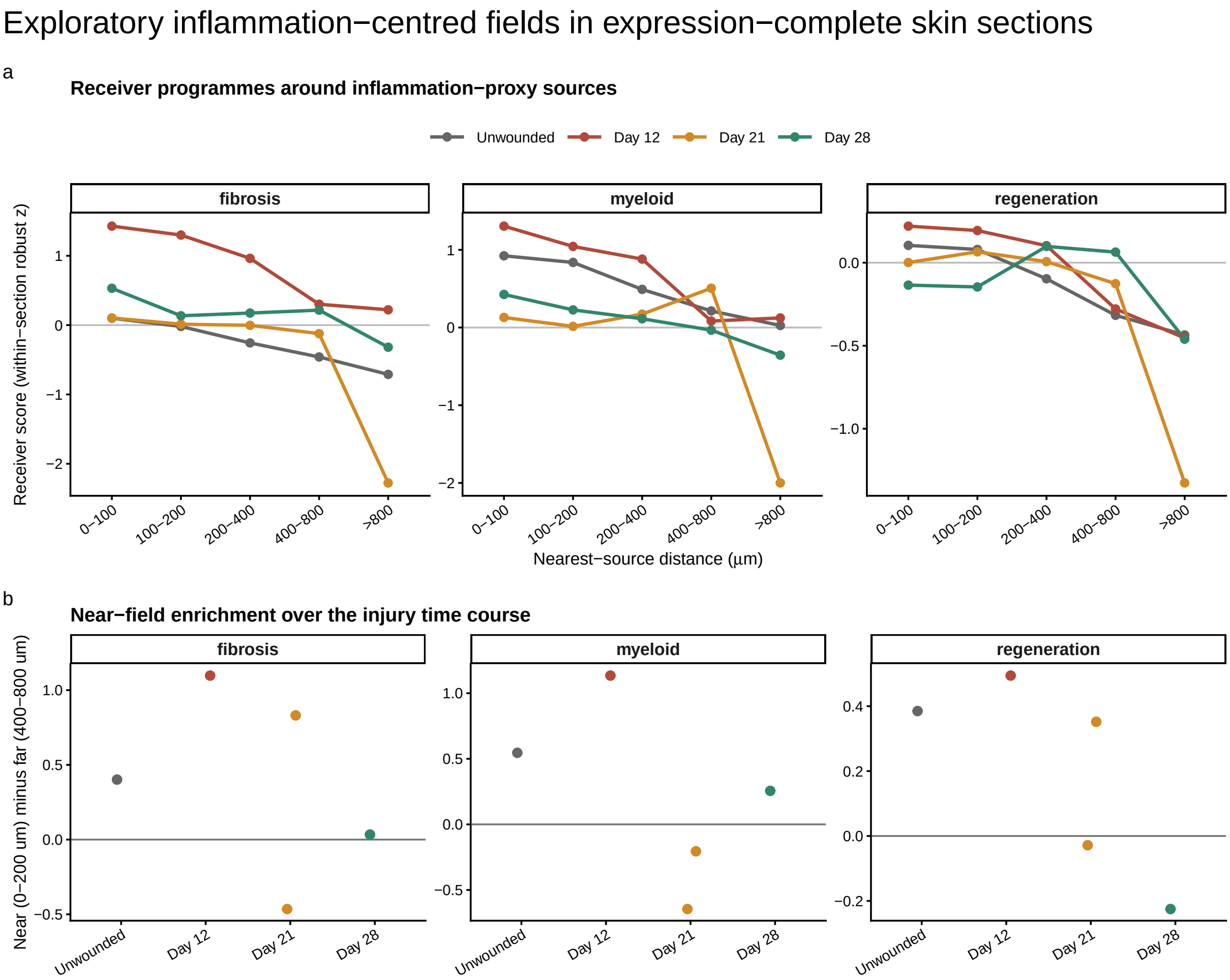

Supplementary Fig. 9: Skin-wound proxy fields. Five integrity-passing sections, scored using log of the mean normalized count, including unwounded and day-12, day-21 and day-28 samples, showed heterogeneous profiles. They did not establish persistence or propagation speed without repeated sampling of the same wound.

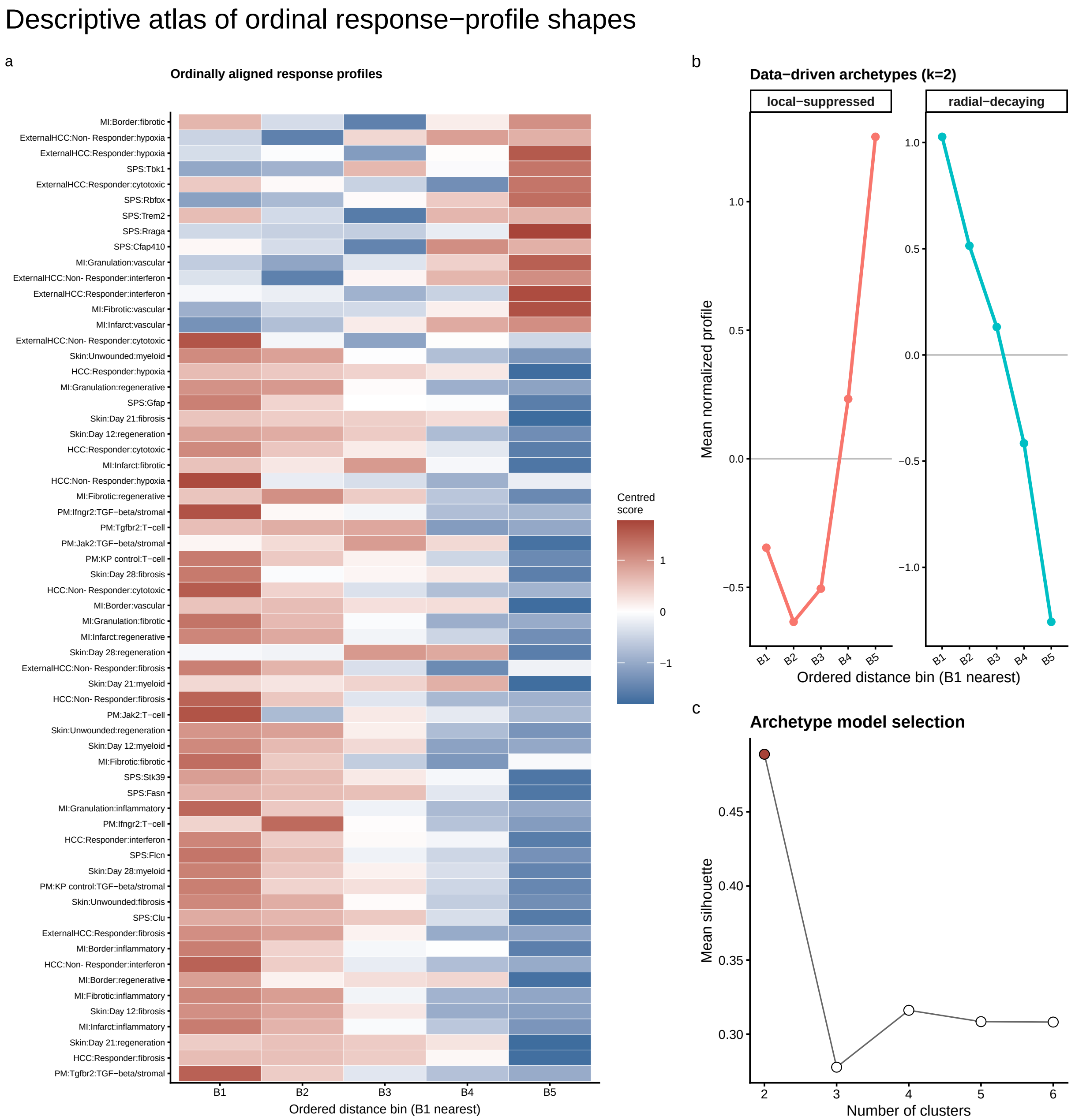

Supplementary Fig. 10: Response-wave atlas. Sixty-two complete curves aligned as ordinal B1–B5 intervals resolved two descriptive shapes (mean silhouette 0.489). Original coordinate units are retained; no shared physical radius is assumed. Cluster labels describe curve geometry and are not interpreted as two distinct transport mechanisms.

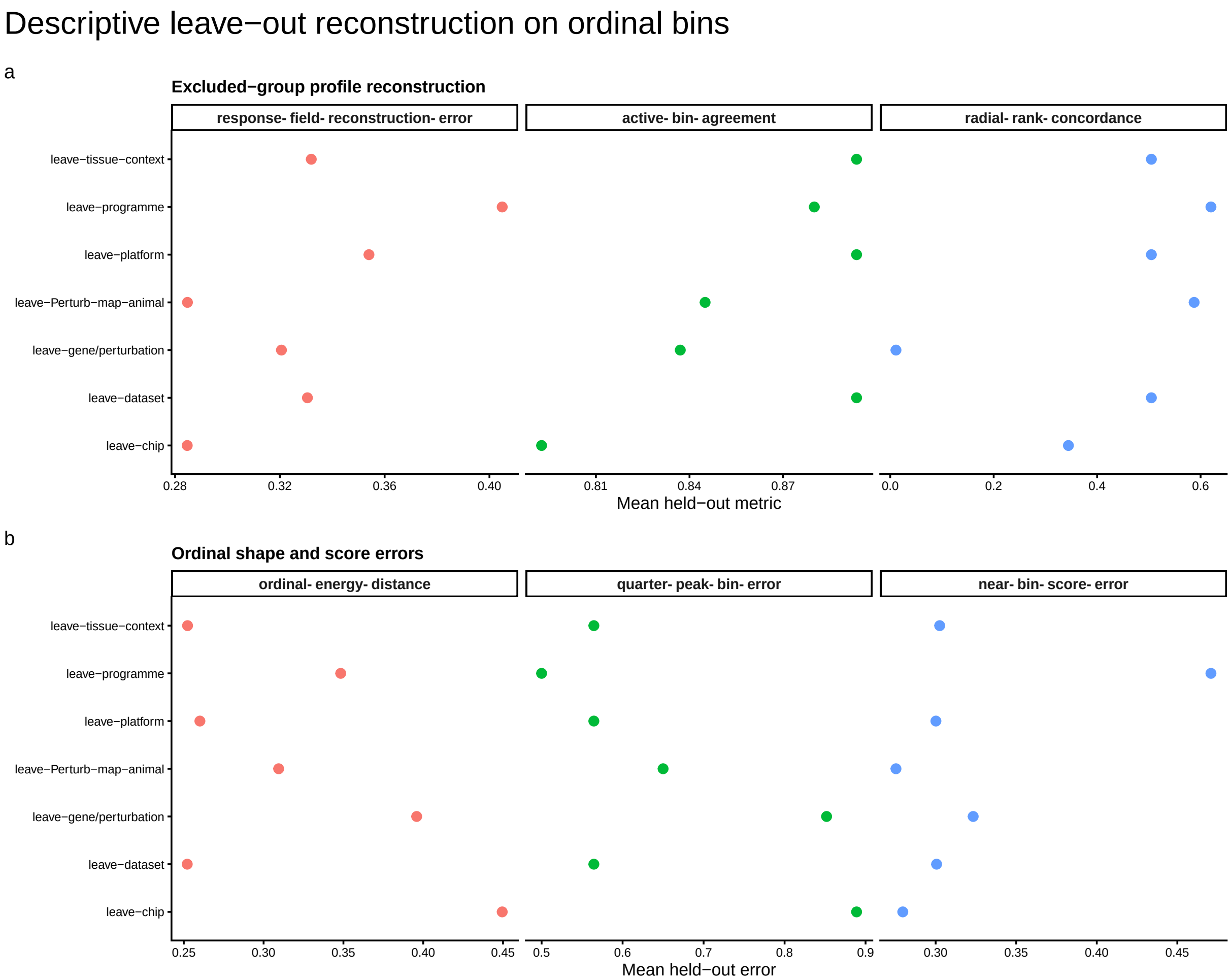

Supplementary Fig. 11: Leave-out reconstruction validation. Ordinal five-bin RMSE, energy distance, active-bin agreement, quarter-peak bin error, rank concordance and near-bin score error are descriptive; no physical cross-assay radius or boundary accuracy is inferred. The corrected platform holdout has two technology groups: Stereo-seq and Visium, with myocardial infarction, skin, Perturb-map and both HCC cohorts withheld together as Visium. The scalar identity predictor excludes its target identity (SM10). These checks do not establish biological cell-type or platform generalization.

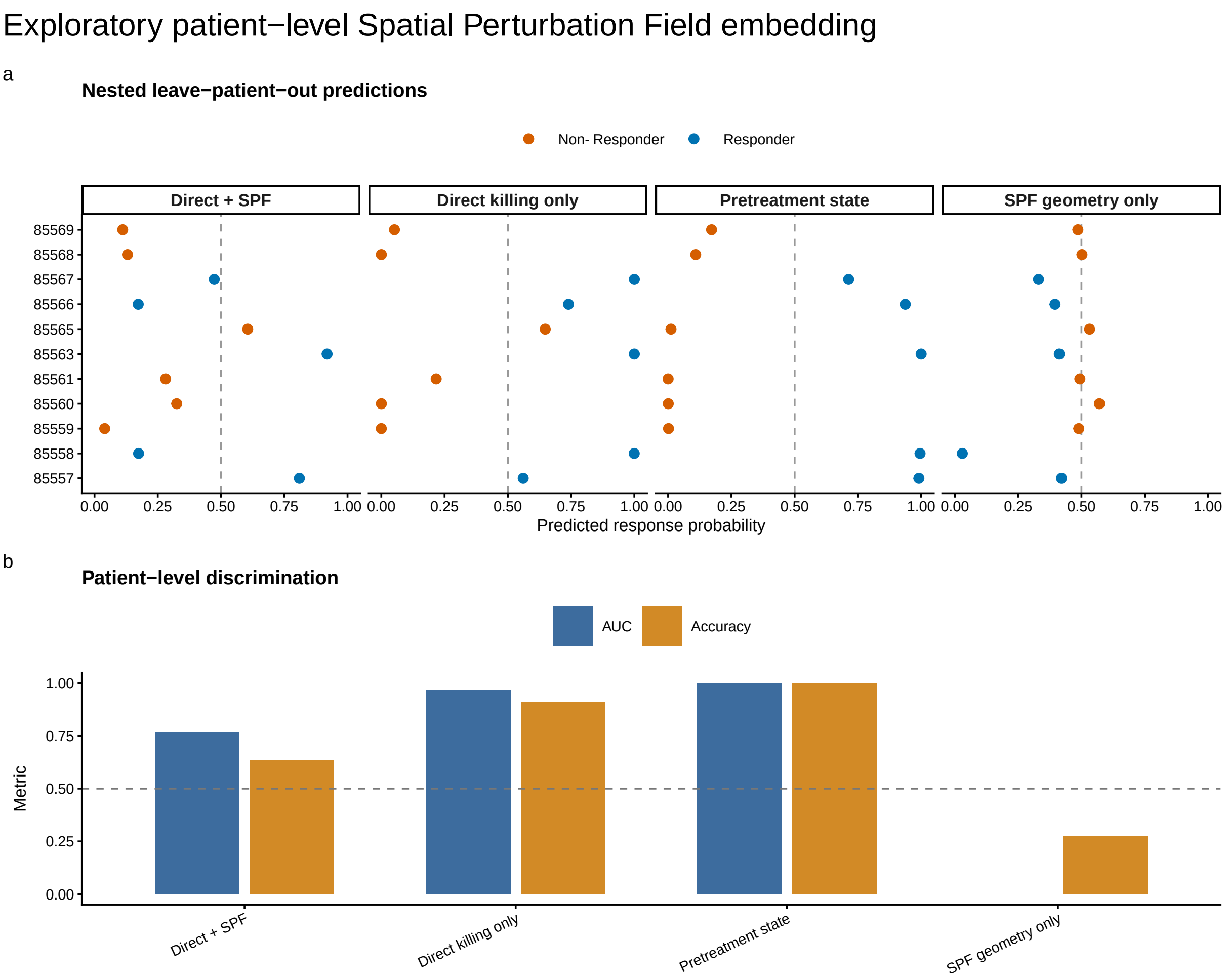

Supplementary Fig. 12: Nested patient-level treatment-response prediction. Eleven patient-level outer predictions compare direct, pretreatment and corrected receiver-only field features. With isotropic hex coordinates, SPF-only AUC/Brier are 0/0.3652; combined values are 0.7667/0.2062. Image-pixel coordinates give the same AUCs. The intercept-only LOPO diagnostic also has AUC 0 because fold training proportions depend on the held-out class; no post-hoc prediction reversal was made. The positive label is the supplied Responder outcome. AUC, 0.5-threshold accuracy and Brier score are defined in SM10.

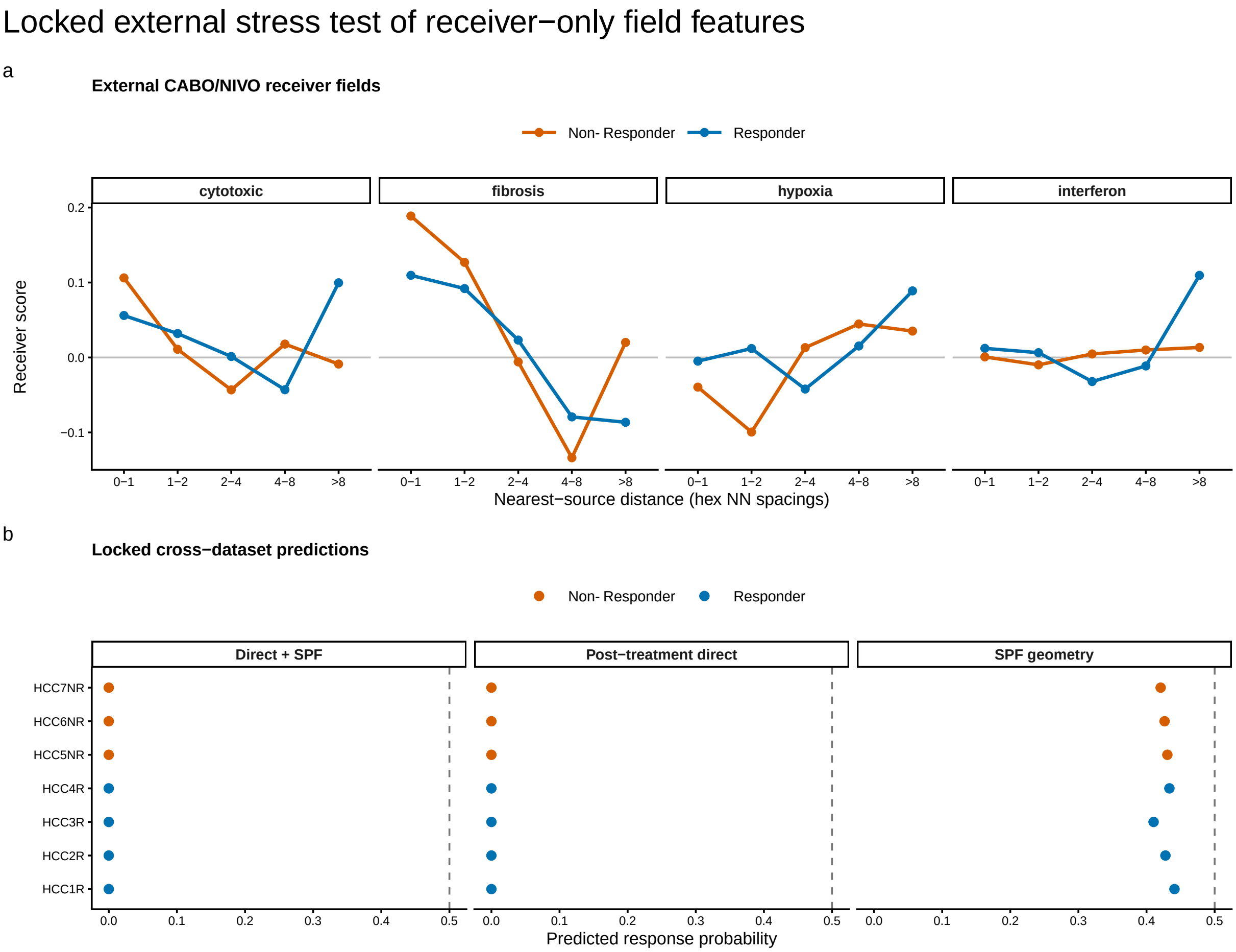

Supplementary Fig. 13: Locked external HCC validation. Preprocessing and coefficients from 11 training patients were fixed before application to seven external cabozantinib/nivolumab samples; with corrected hex coordinates, receiver-only SPF and combined AUCs are 0.6667 and 0.5833 (exact one-sided references 11/35 and 15/35); Brier values are 0.2647 and 0.5714. The image-pixel sensitivity has the same AUCs and reference fractions. This does not establish a clinical biomarker or consistent incremental performance. External AUC inference permuted sample outcome labels at fixed predictions (SM10).

SM12. Known-effect benchmarks and background sensitivity

Design and implementation. The single synthetic fit in R/02 illustrates estimator behaviour but does not estimate type-I error or power. A separate repeated benchmark used 14 parameter configurations, each with 100 independent base realizations, evaluated at injected peak amplitudes 0, 0.5 and 1. Receivers occupied a jittered 28×28 lattice in a $2,400 \times 2,400$ μm domain, with a 21×21 candidate-source lattice. Removing the central band produced 660 rather than 784 receivers in the disconnected configuration. The background combined nonlinear spatial functions, independently sampled low-frequency spatial coefficients, broad receiver identity, log library size and Gaussian noise with SD 0.8. A source-associated increment $a \exp[-d(i, S)/r]$ was added using known amplitude a and scale r; disconnected components had no cross-component exposure. Boundary crossing attenuated the injected increment by 80% in the boundary configuration. The baseline had 12 sources in nominal placement groups of size 3 and $r = 150$ μm.

The one-factor parameter configurations changed source number (6 or 24), nominal group size (1 or 6), boundary attenuation, tissue disconnection, field scale (75, 300 or 600 μm), spatial segregation of receiver identities, source anchors restricted to one coarse x-coordinate niche, 25% identity mislabelling, or 50% score masking. Masking is a score-level dropout proxy, not a sequencing-count model. Synthetic group membership was supplied by the generator's nearest-candidate placement groups; it was not re-estimated with the real-data 100-μm graph. Candidate spacing was 116 μm, so a 150-μm adjacency connects cardinal neighbours. Groups may touch and merge. This is a small conditional benchmark, not a full factorial exploration of tissue architectures or assay noise.

All approaches used the same unweighted contrast, receivers at $d \leq 100$ μm minus those at $400 < d \leq 800$ μm. The comparison therefore isolates specified nuisance models and source generators rather than differences between endpoints. Four generators used unrestricted source labels, component-specific source counts, component-specific nominal cluster-size placements, or the latter with source-anchor niche matching. The default source design fixed the numbers of anchors in each coarse niche; the source-niche configuration restricted all anchors to one niche. Component-only generators deliberately omitted this extra conditioning. Cluster placement uses a seed and its nearest unused candidate neighbours. It matches sizes at placement, not exact cluster shapes, pairwise distances, local density, or the connected-component sizes after placements merge. Niche matching conditions on known supplied coarse strata; it does not recover an unknown latent niche.

For each outcome we compared no background correction, linear adjustment for identity, log library size and x/y coordinates, and Gaussian GAM adjustment for identity, $s(\log L, k = 5)$ and $s(x, y, k = 30)$. The benchmark used `mgcv::gam` with REML; the original full-cell analysis used `bam` with fREML and discrete fitting. These are sensitivity models, not an assertion that the benchmark reproduces the full raw-data fit. Residual scores were held fixed during all source relocations. Every comparator now uses the same two-sided observed-plus-valid-null common centre and plus-one formula (SM5), with each pseudo-source excluded from its own receiver contrast, with 199 configurations and minimum possible $P = 0.005$. In contrast, the archived source-count screen used an uncentred absolute statistic; its published values were not overwritten by the standardized benchmark. At least five receivers in both windows were required in this small simulation; an unsupported contrast returned NA. This support rule is not a biological sample-size requirement.

The fixed seed was 20261015, with recorded deterministic offsets by scenario. All 50,400 test results and all 504 condition summaries are provided. Wilson 95% intervals describe data-realization Monte Carlo uncertainty conditional on each common 199-configuration bank, fixed layout and identity labels. They do not include variation from a newly drawn null bank, generator misspecification, model selection or biological sampling. The approximate source generator, common centring and within-configuration source exclusion do not supply a general exact-randomization theorem.

Complete reproducibility replay. A same-seed replay reproduced the initial generator's 50,400 records and 504 summaries, apart from mean-effect rounding below 10^{-15}. The generator using the common-centre and pseudo-source-exclusion specification (SM5) was run from seed 20261015 across 14 scenarios, 100 base realizations, three amplitudes, three background models, four source generators and 199 configurations. This run generated 50,400 records afresh, and its 504 summaries were independently reconstructed. Results differ from the initial generator where the specification changed. Reusing a seed does not create 200 or 300 independent realizations per condition or new biological replication.

Calibration, power and failure regimes. With cluster-size placement and GAM adjustment, baseline zero-increment rejection is 1/100 (Wilson 95% interval 0.0018–0.0545); the independent-placement implementation yielded 2/100.

Supplementary Table 13
Conditional rejection and detection of cluster-size placement plus GAM adjustment. Each entry is the rejection proportion and Wilson 95% interval from 100 base realizations; the nonzero peak amplitude is 1 in generator score units. These intervals condition on the shared 199-draw null bank. The disconnected zero-increment result demonstrates incomplete nominal calibration. All background and null combinations are retained as `synthetic_expected` in public repository input `data/endpoints.rds`; `Rscript run.R stats` writes `synthetic_summary.tsv`.

Configuration	Zero increment	Amplitude 1
Baseline	0.01 (0.00–0.05)	0.74 (0.65–0.82)
6 sources	0.03 (0.01–0.08)	0.46 (0.37–0.56)
24 sources	0.08 (0.04–0.15)	0.91 (0.84–0.95)
Singleton sources	0.06 (0.03–0.12)	0.90 (0.83–0.94)
Nominal cluster size 6	0.03 (0.01–0.08)	0.55 (0.45–0.64)
Boundary attenuation	0.01 (0.00–0.05)	0.73 (0.64–0.81)
Disconnected tissue	0.10 (0.06–0.17)	0.66 (0.56–0.75)
75 um field scale	0.04 (0.02–0.10)	0.68 (0.58–0.76)
300 um field scale	0.06 (0.03–0.12)	0.47 (0.38–0.57)
600 um field scale	0.02 (0.01–0.07)	0.26 (0.18–0.35)
Segregated receiver composition	0.04 (0.02–0.10)	0.79 (0.70–0.86)
Source–niche association	0.02 (0.01–0.07)	0.61 (0.51–0.70)
25% identity error	0.02 (0.01–0.07)	0.80 (0.71–0.87)
50% score masking	0.02 (0.01–0.07)	0.30 (0.22–0.40)

Detection is 21/100 at amplitude 0.5 and 74/100 (0.646–0.816) at amplitude 1. At amplitude 1 it is 46/100 with six sources, 91/100 with 24 sources, 26/100 at the 600-μm scale and 30/100 under score masking. The disconnected condition rejects 10/100 zero-increment realizations (0.055–0.174). Across all 168 zero-increment parameter–background–generator conditions, 61 observed rates exceed 0.05, and 24 Wilson lower bounds exceed 0.05 without multiplicity correction. These correlated conditions are not independent experiments; they describe settings in which calibration is limited.

In the source–niche condition without background adjustment, component-count and cluster-size placement reject 15/100 and 20/100 zero-increment realizations; adding source-anchor niche matching gives 5/100. With GAM adjustment the cluster and niche versions give 2/100 and 3/100. Baseline linear adjustment yields 13/100 for component-count and 8/100 for cluster placement. Anchor-niche placement plus GAM still rejects 10/100 in the singleton condition (0.055–0.174); its baseline amplitude-1 detection is 77/100, distinct from the cluster-only 74/100. Additional conditioning or a successful baseline does not establish valid source exchangeability on every tissue.

Known-increment injection on the real Clu map. The real-map injection diagnostic represents one chip, B03018A2, containing 123,921 cells, 119,664 guide-undetected controls and 419 observed Clu sources. We kept all source positions and sampled 2,500 controls as receivers and 8,000 controls as candidate source locations. Observed 250-μm grid occupancy reconstructed three tissue components; this corresponds to the original 500-native-coordinate grid at 0.5 μm per coordinate. The observed source graph had 241 components at the 100-μm adjacency threshold, with largest size 16. The sampled near and far windows contained 303 and 719 receivers. No injected signal was allowed to cross disconnected components. The frozen map, candidate pool and cluster specification are retained as `real_injection_design` in the public input `data/endpoints.rds`; seeds and injection operations are implemented in `R/injection.R`.

The injection grid added peak increments of 0, 0.5, 1 or 2 to the original receiver score at scales of 75, 150, 300 or 600 μm. A fixed-background variant fitted the unmodified score once and retained that fitted background after injection; a refit variant re-estimated the background for every increment. Fixed fitting provides a no-refit signal-retention comparator, while refitting quantifies attenuation when a true increment aligns with tissue structure. Library size was not used in this injection diagnostic, which adjusts for identity and position only; it is separate from the original identity–library–position fit. The full-chip compact input includes native whole-gene library counts for regenerating that fit.

With GAM adjustment and cluster placement, the unmodified map had null-adjusted effect −0.0315 and conditional reference fraction $P = 0.775$. Adding peak amplitude 1 at 150 μm gave effect 0.6076 and $P = 0.005$ with fixed

background, and effect 0.4690 and $P = 0.005$ after refitting. Relative to the unmodified map, the refitted near–far increment retained 82.1%, 73.7%, 67.7% and 63.3% of its fixed-background counterpart at scales 75, 150, 300 and 600 µm, respectively. For the 150-µm, unit-amplitude injection, the observed near–far contrasts before source-reference subtraction were −0.024126413 without injection, 0.613136074 with the original background fixed, and 0.445784511 after background refitting. The retained contrast gain was therefore $(0.445784511 + 0.024126413)/(0.613136074 + 0.024126413) = 73.7\%$, distinct from the ratio of the source-reference-adjusted effects reported above. At 600 µm the refitted P was 0.015. Thus background correction can detect a known increment and simultaneously remove a substantial part of it. An absent strict Clu result cannot prove absence of every biological receiver response.

These deterministic injections assess sensitivity on one observed tissue. The original score may already contain unmeasured source-associated structure, so its zero-injection P is not a biological type-I-error estimate, and the injection table is not a repeated-sample power estimate. Known-truth type-I error and power are measured only in the synthetic generator. No biological ground truth, new animal replication or molecular-transport mechanism is supplied by the injection.

Observed-source dropout sensitivity. Because score masking does not represent failed guide detection, a separate one-seed source-label thinning check used the same real-coordinate, peak-1, 150-µm injection. The true response remained generated by all 419 sources, while a nested random ordering retained 419, 314 or 209 observed source labels (0%, 25% or 50% removal). Receiver identities, scores and eligibility were fixed; observed distances, source-graph clusters and 199-draw source banks were recomputed. Lost sources were not added to the receiver set, so this check isolates exposure-location error and does not model contamination by directly perturbed, guide-undetected receivers.

The observed source graphs had 241, 215 and 157 clusters; near/far receiver counts were 303/719, 257/794 and 188/840. The known injected near–far increment decreased from 0.6373 to 0.6137 and 0.5701. GAM-refitted cluster inference nevertheless gave null-adjusted effects 0.4564, 0.4897 and 0.5040, all at $P = 0.005$ under these separately seeded banks. Thus detection was retained in this particular example, but the estimated contrast did not decline monotonically with missing source labels. No general dropout robustness or power conclusion is inferred from one seed and one tissue. The public `Rscript run.R dropout OUTPUT` command regenerates `guide_dropout_sensitivity.tsv`; the source-retention seed and implementation are specified in `R/injection.R`.

Software comparison and reproducibility limits. The controlled known-truth comparison uses explicit unrestricted-label, count, cluster and source-niche generators; these R results are not attributed to external software. We separately executed the complete installed Squidpy 1.6.5 package through its public `gr.nhood_enrichment` API on the full exported B03018A2 chip. All 119,664 guide-undetected receivers and 419 Clu sources entered a 120,083-node graph. The mask used all 123,921 mapped cells and the original 250-µm occupied-grid connectivity, giving three components. A symmetrized up-to-six-nearest-neighbour graph within component had 819,914 directed adjacency incidences (409,957 undirected edges), without self or cross-component edges. Receivers above the full receiver median of zero were high, and the remainder low, with 59,832 receivers in each category. Native inference used `library_key=component`, 499 within-component permutations and seed 20261015.

The full-chip native source-to-high adjacency count was 1,576 with $z = 4.8719$; source-to-low count was 1,247 with $z = -5.8127$. Source self-adjacency had native diagonal count 130 (65 undirected edges), with $z = 26.3545$. The API returned counts and null-standardized z scores, not empirical P values; no Gaussian P values were inferred. The high-score adjacency and archived full-chip strict Clu $P = 0.91$ describe different estimands and controls: categorical edge incidence under component-wise label shuffling versus an identity–library–position-adjusted continuous near–far contrast under cluster placement. Neither test establishes biological ground truth. The native enrichment is not labelled a known false positive or a propagation measurement.

The sampled-graph analysis provides a sensitivity comparison. Keeping all 419 sources but only 2,500 receivers raises the source fraction from approximately 0.35% to 14.35%, changes nearest-neighbour relationships, and changes the global receiver median to 0.0200055. That graph has source-self count $1{,}408/z = 36.1100$, source-to-high count $836/z = -16.6235$, and source-to-low count $800/z = -17.4162$. This reversal shows that the full-chip and sampled graph endpoints are not interchangeable; sampling, graph construction and the associated threshold changed together. The sampled identity-specific median endpoint is also reported. The shared cell input and `optional/native_squidpy.py` and `native_sampled.py` regenerate both graphs and native matrices. Pinned executed versions are recorded; a fresh installation was not tested. The public repository includes the native graph scripts and dependency records; large NPZ/H5AD caches and complete environments are not repository inputs. Giotto and other named packages were not

run. Native Squidpy was run on all 600 generated cases from the count-level guide-error suite (SM13). Its categorical adjacency endpoint differs from the continuous field endpoint, so the results do not rank methods on a common estimand or establish general native calibration.

The public numerical repository contains the revised source-reference code, fresh benchmark records and object-level provenance. Audit execution covers raw five-mouse reconstruction, independent foldwise preprocessing, Clu/DBiT references, raw-count Perturb-map endpoints, corrected clinical/atlas models and fixed sensitivity grids. The repository includes core and interface checks; its `VALIDATION.md` distinguishes completed executions from reconstruction routes. Feature-level compact inputs are distinguished from raw-data reconstruction: the smallest foldwise count input refits Clu/mSafe, while other-guide full refits require the public raw matrices. Native units and whole-animal profile holdouts are retained. The complete new computational checks and unresolved experimental/metadata needs are given in SM13. The public numerical repository, version and fixed commit are given in the main Code availability statement; methods, primary results and sensitivity summaries are reported here and in SM13–SM15.

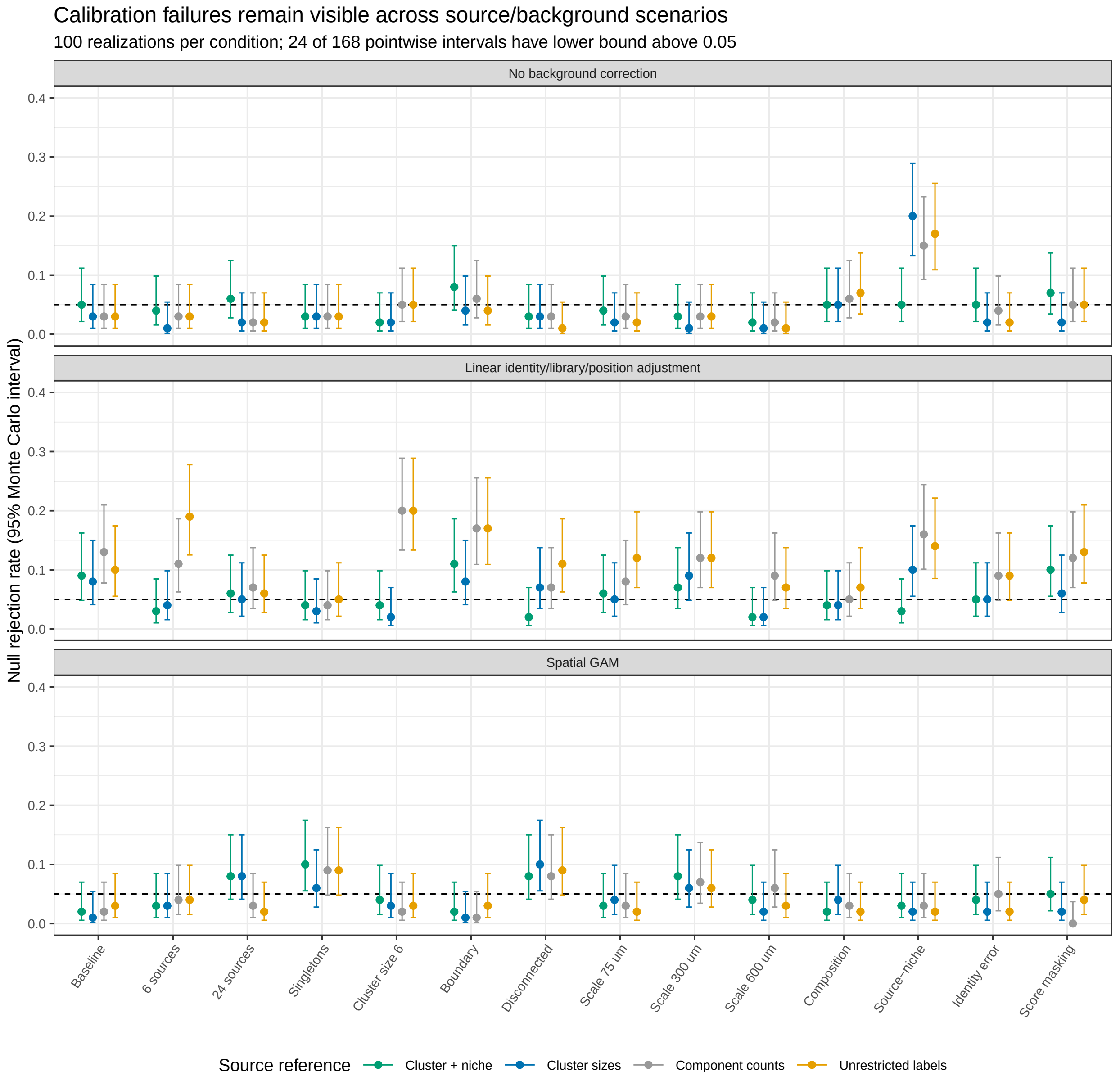

Supplementary Fig. 14: Zero-increment rejection rates across all synthetic parameter configurations. Points and Wilson intervals summarize 100 realizations per condition, conditional on the shared 199-draw source bank, using common observed/null centring and excluding selected pseudo-sources from each receiver statistic. The dashed line marks 0.05. Unrestricted, component-count, cluster-placement and cluster-plus-anchor-niche generators use the same contrast. Both successful and misspecified regimes are retained.

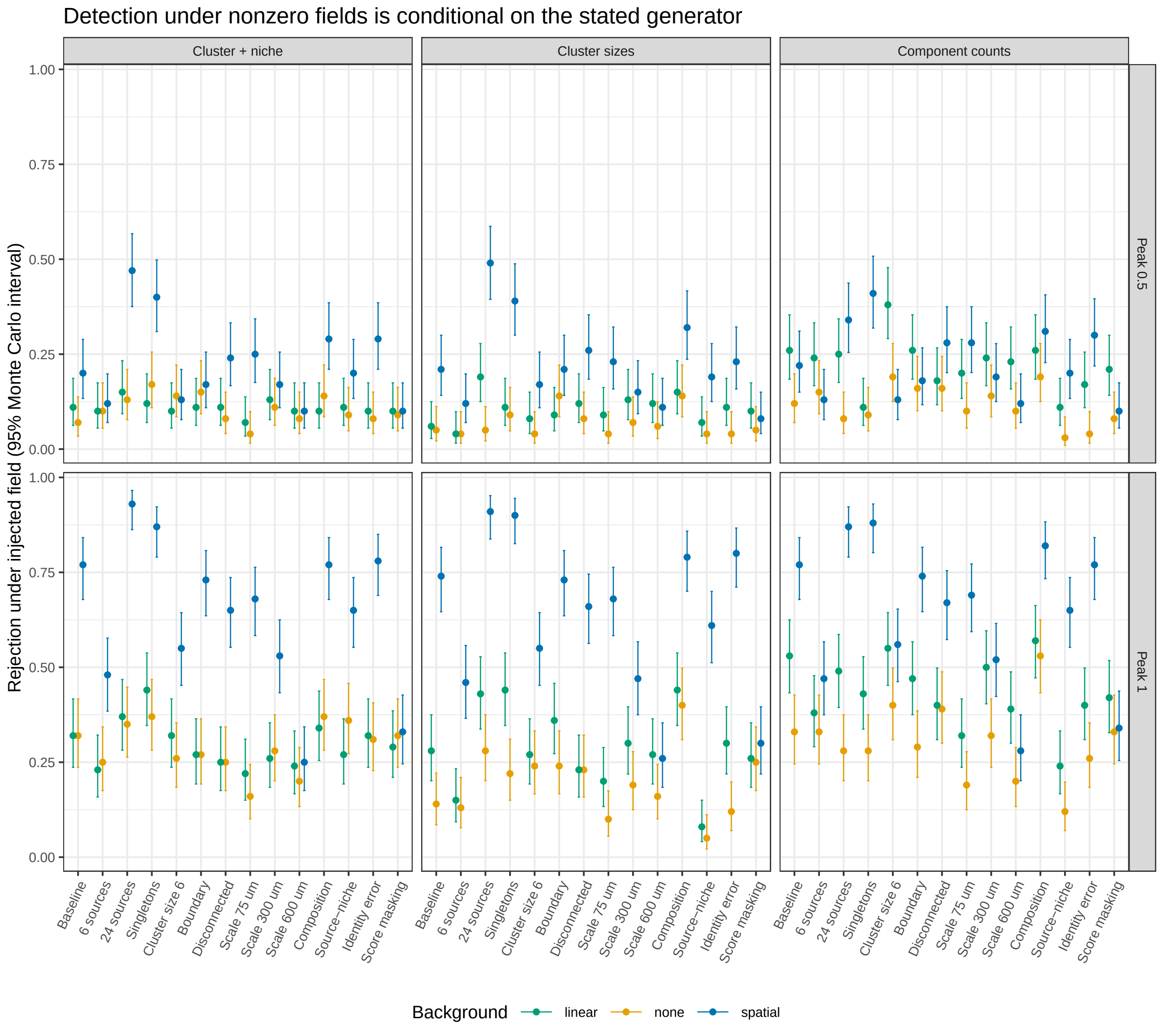

Supplementary Fig. 15: Known-increment detection in the synthetic experiment. Rows show peak amplitudes 0.5 and 1; columns show component-count, cluster-placement and anchor-niche-conditioned nulls. Background correction is varied without changing the response contrast. Intervals are conditional Monte Carlo intervals, not biological confidence intervals.

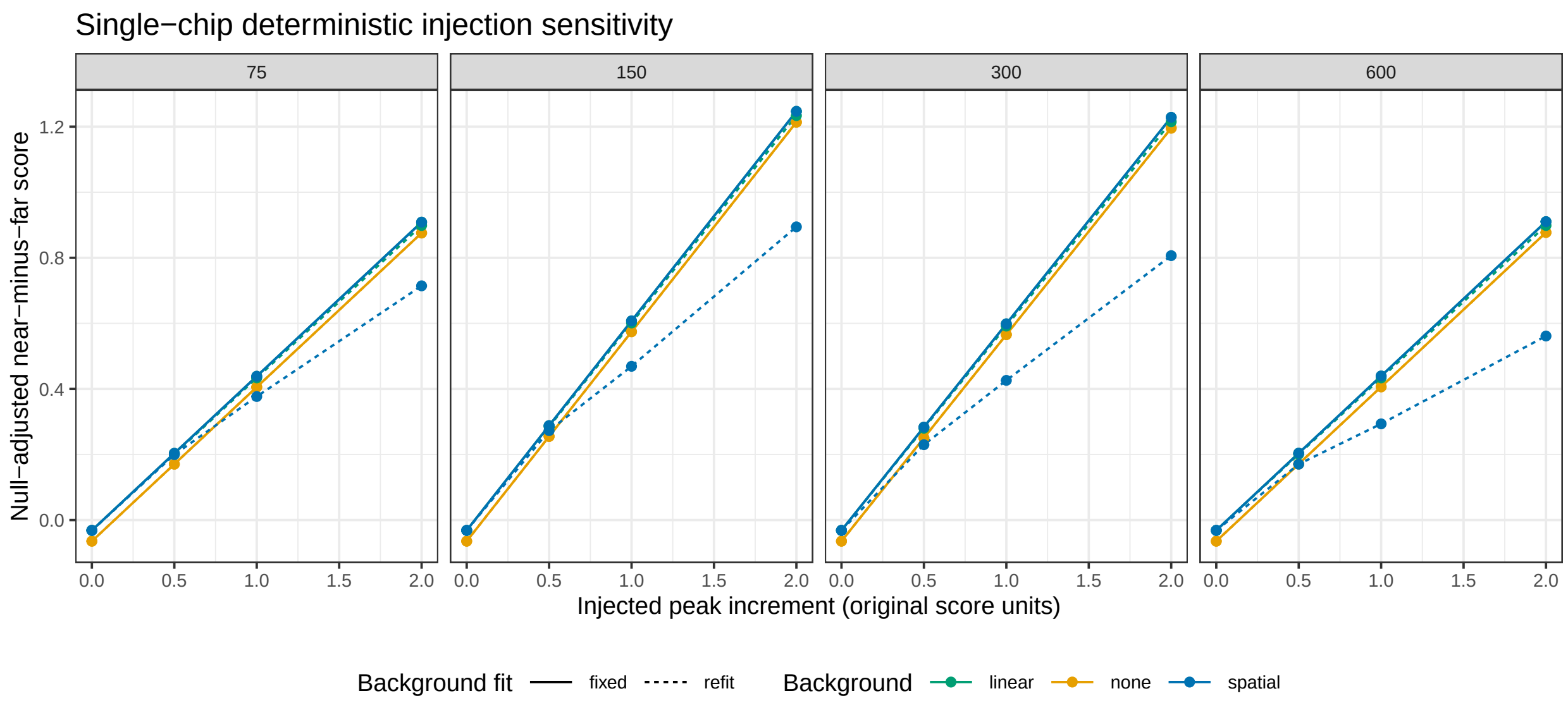

Supplementary Fig. 16: Single-chip known-increment sensitivity on the exported Clu coordinate map. All 419 observed sources are retained; receiver and candidate-control samples and the seed are recorded. Lines compare background fitting fixed before injection with background refitted after injection. Broad injected signals are partly absorbed by the refitted spatial GAM. The original signal is not assumed to be a known biological null.

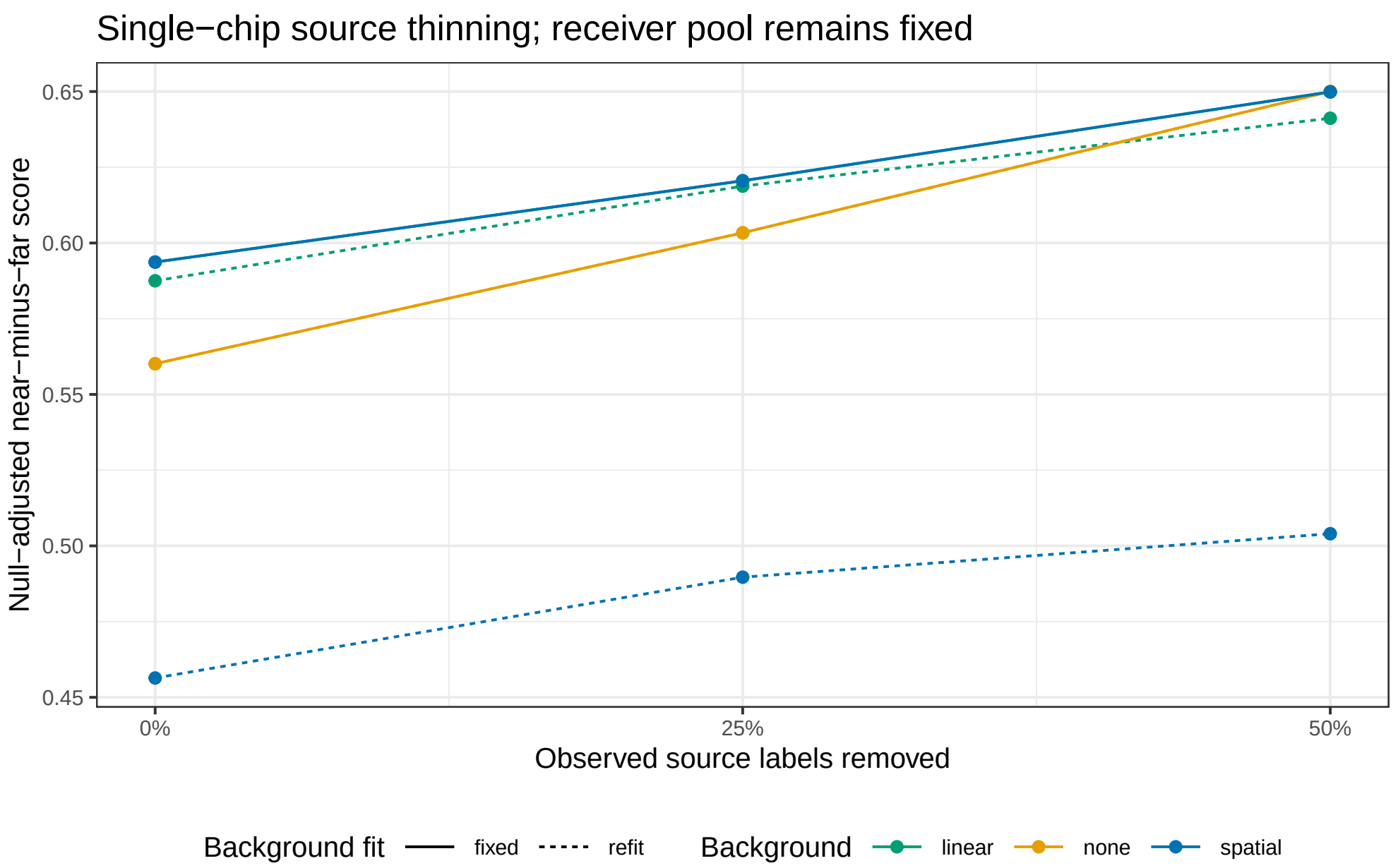

Supplementary Fig. 17: Sensitivity to missing source positions in the single-chip known-increment example. The true injected score is generated by all 419 sources, while only 419, 314 or 209 sources remain observed. Distances, clusters and source nulls are recomputed. This one-seed exposure-misclassification sensitivity keeps receiver eligibility fixed and does not model lost-source contamination; the separate repeated count-level contamination experiment is reported in SM13. It is not a power or calibration estimate.

SM13. Additional computational checks, support failures and remaining evidence needs

The following revision-time protocols were fixed before inspecting their new outcomes. They are computational extensions and exploratory sensitivity grids, not prospective biological preregistration. Completed calculations, unavailable endpoints and failures are reported together. Repeated configurations, folds, programme choices and software calls do not create additional animals or patients.

Common reference centre and configuration-specific receiver exclusion. The source-reference implementation now uses the common observed-plus-finite-null centre in SM5, while reported excess remains observed minus the null mean. Each selected pseudo-source is excluded from its own all-receiver, identity, profile and directional statistics. Scores and the GAM fitted on the original receiver population remain fixed. The source sampler, nominal cluster sizes, candidate pool, seeds and observed statistics are otherwise unchanged; this correction does not prove observational source exchangeability. For Clu the all-receiver excesses are 0.0078115, −0.0024411 and 0.0010659 in C02943C3, B03018A2 and A03599E2; their reference fractions are 0.78, 0.91 and 0.99 with 99 configurations each. None of the seven within-chip endpoints has a descriptively BH-adjusted reference fraction below 0.05 (minimum 0.07); these fractions are not established FDR control (SM14). Directional fractions are 0.93, 0.13 and 0.70. The descriptive quarter-peak radii of 200, 200 and 400 μm are not established propagation distances for unsupported fields.

The same correction was applied to Perturb-DBiT without changing its programme choice or observed contrasts. Bru.LC.6 has 398 finite endpoints from 500 attempted configurations and fraction 304/399; its 102 unavailable configurations remain in the audit. Bru.LC.8 has 500/500 finite endpoints and 498/501. The two liver sections have 1,000 finite configurations each and fractions 381/1001 and 615/1001. These are conditional section diagnostics; Bru.LC.6/8 share block 33-1 and the liver sections share MSTGN3R. In DBiT, candidates include units outside the original receiver pool, so the number additionally excluded can be smaller than the source count. Every configuration retained the requested source count and unique indices. Independently reading back 3,297 configurations and the complete reference tables gave 3,336 passing checks, including zero pseudo-source/receiver overlap. Neither this correction nor the negative references supplies independent animal replication.

Strict foldwise mouse validation. Five raw 10x mouse inputs were used in five separate training/query splits. Every training fold rebuilt the four-mouse gene filter, Seurat object, fixed cell QC, LogNormalize at 10,000, vst 3,000 variable features, scaling, 40-PC fit, Harmony on PCs 1–35, neighbours on dimensions 1–30, clustering at resolution 0.6 and UMAP. PCA seed was 42, Harmony inherited the ensuing RNG state, and clustering/UMAP used seed 17. Query data did not fit gene selection, means/SDs, PCA, Harmony, clustering, DE, mSafe subtraction or thresholds. Query QC used the training-retained RNA genes; normalized query HVGs were centred/scaled with training statistics, clipped above 10 as in the training scale operation, and multiplied by the training PCA loadings. The fitted Harmony workflow did not provide a projection operator for an unseen animal.

The closest-original route uses the fold-specific training Harmony coordinates for training-mouse matching and frozen training-PCA coordinates for within-query-mouse matching. An auxiliary route uses the same frozen PCA map for both training and query. Both retain input-order greedy matching among the 50 nearest available guide-undetected controls, no replacement within a mouse/guide, at least three target cells, at least three training mice, and every DE/mSafe/gene threshold in SM2. All 17 non-mSafe guides, two routes and two gene variants were run (340 rows). Training sets contain 30,731–37,057 cells and 23,724–24,058 RNA genes; queries contain 4,082–10,409 cells. B3-1 has one fewer query cell than the joint-reference QC because of the training-only gene filter and fixed feature threshold, not an outcome-driven exclusion. All training cells were checked by blocked projection against their fitted PCA; the largest difference was 4.50×10^{-12}.

Only 16/55 original-programme/mouse combinations are estimable in this primary no-IEG route. Thirty-three lack the required mSafe training support; six more fail programme, target-training or query support. For Clu, B1-1 gives six no-IEG genes, 9/9 target/control cells, AUC 0.530864 and standardized mean difference 0.074541; B2-2 gives 13 genes, 8/8 cells, AUC 0.359375 and standardized difference −1.582504. The auxiliary no-IEG route gives AUCs 0.580247 and 0.359375 (five/13 genes). The all-gene primary variant has seven/16 genes and AUCs 0.506173/0.281250. B1-2, B2-1 and B3-1 remain unestimable because excluding one of the three eligible mSafe mice leaves only two. Combinations without the required support remain unestimable; no blacklist fallback, threshold relaxation or route selection was applied. The shared-preprocessing diagnostic in Table 3 uses a different protocol from the new-query extension and is not an out-of-sample Harmony prediction. These results do not establish stable mouse transfer or receiver propagation.

Supplementary Table 14
Complete fold support for the original 11 programmes under the primary training-Harmony/query-PCA route with immediate-early genes removed. AUC ranges cover estimable folds only. An unavailable fold is retained as unavailable, not zero AUC or a negative biological finding.

Programme	Estimable folds / attempted	Held-out AUC range
Cfap410	1/5	0.500
Clu	2/5	0.359–0.531
Dpp6	2/5	0.333–0.500
Fasn	2/5	0.333–0.516
Flcn	2/5	0.375–0.480
Gfap	2/5	0.500–0.673
Rbfox	1/5	0.167
Rraga	1/5	0.560
Stk39	2/5	0.281–0.520
Tbk1	0/5	Unavailable
Trem2	1/5	0.375

For these foldwise score results, the standardized difference is the target-minus-control mean divided by the held-out control MAD with factor 1.4826. A non-finite MAD or one below 10^{-8} uses the control SD as fallback; a non-finite or non-positive fallback leaves the standardized difference unavailable. This differs from the pooled-SD matching-balance SMD in SM2. The raw/public CLI completed every fold and the complete refit. Independent checks comprised 297 model/matching/score checks, 1,221 direct raw-count export checks and 48 numerical equivalence checks for the memory-blocked implementation, all passing. These are 1,566 computational checks, not independent validation experiments. The smallest count object retains only Clu/mSafe full-gene training pseudobulks and held-out Clu/control counts; it independently reproduces all 20 Clu rows. A separate small snapshot preserves all 340 metrics, estimable scoring weights and support. Other-guide count refits require the original public matrices and `optional/full_holdout.R`; a frozen snapshot is not described as their count-level reanalysis. No full Seurat object is bundled.

Guide-count error and directly perturbed receiver contamination. The one-seed missing-position experiment in SM12 holds receiver eligibility fixed. A separate repeated count-level model now explicitly assigns false-negative true sources to observed receivers and false-positive non-sources to observed sources. Each base realization has 324 positions on an 18×18 lattice from 40 to 1,400 μm per axis with independent ±5-μm uniform jitter, and 24 latent sources in six nominal size-four placement groups. Groups can merge; they are not necessarily six connected clusters or biological clones. Identity A is sampled with probability $\text{logit}^{-1}[(x_i - 720)/350]$; $\ell_i = 8.5 + 0.15\mathbf{1}(A_i) + \epsilon_i$ with $\epsilon_i \sim N(0, 0.25^2)$, and $L_i = \text{round}(\exp \ell_i)$. The count background is $B_i = \sin(x_i/390) + 0.7\cos(y_i/430) + 0.8\mathbf{1}(A_i) + 0.2(\ell_i - 8.5) + Z \sin[(x_i + y_i)/500]$, with realization coefficient $Z \sim N(0, 0.2^2)$. Source placement uses two coarse x-coordinate niches divided at 720 μm, with all anchors in the right niche in the niche case. Six cases cover ideal detection, nominal dropout 0.25/0.50/0.75, nominal dropout 0.50 plus false-positive probability 0.01, and nominal dropout 0.50 with source-niche restriction. The seed is 20261016 plus recorded realization/case offsets, with 199 source configurations. A library-depth factor $D_i = \exp[0.2(\ell_i - \overline{\ell})]$ modulates guide counts. For non-ideal cases,

$$G_i \sim \begin{cases} \text{Poisson}[-\log(q)D_i], & i \in S_{\text{true}}, \\ \text{Poisson}[-\log(1-f)D_i], & i \notin S_{\text{true}}, \end{cases} \qquad S_{\text{obs}} = \{i : G_i > 0\}. \tag{17}$$

Here q and f are nominal probabilities at $D_i = 1$, not exact realized cell fractions. The ideal case uses guide counts one/zero. A count above one refers to several UMIs for the same focal guide, not a multiple-guide identity. Observed receivers are the complement of S_{obs}, whereas oracle receivers exclude S_{true}. Thus a missed true source retains its direct perturbation effect and enters the observed receiver analysis; a false-positive non-source has no direct perturbation effect in the generating model.

Four equal-mean programme genes follow negative-binomial counts with size 5 and

$$\mu_{ig} = 0.0007 L_i \exp\{0.4 B_i + 0.35 b\, \mathbf{1}(i \in S_{\text{true}}) + 0.35 a \exp[-d(i, S_{\text{true}})/150]\}. \tag{18}$$

Scores are mean gene-wise $\log(1 + 10^4 C_{ig}/L_i)$. The direct parameter $b = 2$ multiplies true-source count means by exp(0.7); it is not an additive score change of two. The extrinsic parameter $a = 1$ changes the log count mean by at most 0.35. These parameters differ from the additive score amplitudes in SM12. The repeated model is deliberately simplified and does not estimate real assay sensitivity, all ambient mechanisms, multiple-guide errors or segmentation errors. Its geometrical latent-kernel contrast is not in the same units as the normalized count score and is not subtracted from score effect as a recovery RMSE.

For each case, 100 base realizations evaluate $(b, a) = (2, 0), (2, 0.5), (2, 1), (0, 0)$, oracle/observed receiver definitions, no/GAM adjustment, and three source generators crossed with retaining/excluding pseudo-sources. This gives 57,600 records and 576 correlated group summaries. Excluding pseudo-sources is the primary rule; retaining them provides an asymmetric-reference sensitivity. Direct-zero/direct-two and retain/exclude comparisons reuse their configuration bank; oracle/observed views use separately sampled banks, and different error cases resample source placement. They are therefore not all single-factor paired comparisons. Each view fits its GAM once and freezes it across source draws. Oracle knows source/receiver truth but still uses the approximate background/source model, so it is not a guaranteed valid null.

The nominal 50% dropout case has realized mean sensitivity 0.5025 and receiver contamination 0.038224; adding nominal 1% false positives gives specificity 0.989367. Under GAM plus cluster placement with pseudo-source exclusion, zero-extrinsic-field rejection in the 50% dropout case is 33/100 for observed receivers versus 3/100 for oracle receivers. Removing the true-source direct effect changes observed rejection to 3/100 while leaving the oracle 3/100, with geometry, guide calls, count uniforms and banks fixed. Thus false-negative sources carrying direct expression changes can be misread as an indirect field in this model. The 33/100 is not an estimated real-assay false-positive rate. With extrinsic amplitude one, observed/oracle rejection is 77/100 versus 12/100; calling the inflated observed value calibrated power would conceal the zero-field failure. All unfavorable results remain reported.

There are 96 missing test records, arising from two distinct geometric failures repeated across analysis/effect branches: oracle replicate 17 of the dropout-plus-false-positive case has only three far receivers, and observed replicate 77 has none, below the minimum five. All groups attempted 100 realizations; affected groups have 99 estimable outcomes. Rejection rates/Wilson intervals use the estimable denominator and retain attempted/failure counts. Finite null counts range from 48 to 199; the core requires at least 20 finite null contrasts. NA is not replaced by zero and new configurations are not drawn until 199 valid ones are obtained. The 324 cells are not independent biological replicates, and 576 summaries are not 576 independent experiments.

Native software on shared generated cases. Squidpy 1.6.5 was actually called through `gr.nhood_enrichment` for 600 generated cases: ideal detection, 50% dropout and source-niche/dropout, each with 100 realizations and extrinsic amplitude zero/one. All retain direct parameter two. A component-restricted symmetric six-nearest-neighbour graph is fixed per geometry, and receiver scores are split at their own median. Native categories are observed source, receiver-high and receiver-low. With 199 within-component category permutations, the frozen rule is $|z_{\text{source,high}}| > 1.96$. Native outputs counts and standardized z, not empirical P; this Gaussian-threshold rule is not an exact permutation test or a continuous field endpoint.

The ideal-case 87/100 and 63/100 exceedances are all source-to-high adjacency depletion. At zero extrinsic amplitude, true source clustering and the direct source effect remain; the native categorical-label null need not hold. Consequently 0.87 is not labelled native type-I error, and 0.63 is not called positive-propagation power. The change after adding a field reflects a different endpoint and conditioning, not an equal-estimand method ranking. Independent one-hot and R graph implementations reproduce all 5,400 count-matrix entries, graph totals and six summary frequencies/Wilson intervals. The single smoke case is excluded from the formal 600.

Rigid-footprint and real-KP controls. The 14 focal Perturb-map annotations comprise four Tgfbr2 and ten KP masks in two mice. A mask is the published set of annotated capture positions, not a newly traced anatomical contour. Original in-tissue/annotated counts are 1,906/1,903, 1,931/1,872 and 2,546/1,355 in KP-1, KP-2 and KP-4. Missing annotation is not assumed to be confirmed lesion-free tissue. Each complete annotation footprint was rigidly transported within its section at six 60-degree orientations, anchoring on every observed mask location; no points/components were dropped or deformed. Physical pair distances were preserved, with discrete lattice coverage requiring all sites

Supplementary Table 15
Native Gaussian-z rule on all 600 generated cases. Every group has 100/100 estimable cases. Counts separate positive and negative exceedances rather than interpreting depletion as detection of positive propagation.

Guide case	Extrinsic amplitude	Absolute exceedance	Positive	Negative
Ideal	0	87/100	0	87
Ideal	1	63/100	0	63
50% dropout	0	24/100	7	17
50% dropout	1	28/100	24	4
Dropout plus niche	0	26/100	11	15
Dropout plus niche	1	36/100	34	2

inside the annotation mask, outside every named lesion and within 0.25 median-neighbour spacing of a capture centre. Pseudo-sources were removed from their own receivers.

All strict/moderate/broad tiers were retained. Calipers were mask-edge distance differences at most 100/200/400 μm, source and near-receiver normal-label fraction differences at most 0.10/0.20/0.30, and near/far receiver-count ratios at most 2/3/4. At least 20 matched placements were required. Of 1,424 complete in-mask footprint placements, only 64 remained after excluding original lesions, covering four annotations. Only Tgfbr2-4-1 had any tier-matched placement (zero/one/two); all other annotations had none. All 252 annotation–tier–programme–endpoint combinations were unavailable. Even a one-placement threshold would not give every class/annotation in any section the support needed for the original section-to-animal estimand. This is an executed positivity failure, not a passed exact-shape null or an omitted computation.

Complementary real-KP matching used source size, mask-edge distance and near normal-label proportion, fixed calipers, no reused control and no expression-outcome selection. It maximized the number of matches before minimizing geometry distance; size-ratio limits were 2/3/4. Strict/moderate/broad support was 1/4, 3/4 and 4/4 mutant annotations; only broad covered both animals. Its equal-animal stromal/T-cell/TGF-beta-target contrasts were 1.492713, −0.093988 and 1.696860. Coarse composition standardization separately estimated normal- and tumour-receiver contrasts (at least five near/far receivers per stratum), then weighted both classes by the same section-wide eligible-receiver proportions. Across all annotations its equal-animal contrasts were 1.207122, −0.156728 and 1.547624; combining broad real-KP matching and this standardization gave 1.272754, −0.124122 and 1.471149. Directions were consistent in both original animals. These sensitivities neither supply extra animals nor resolve fine cell-type mixture/state; size/composition may be treatment consequences, so the adjusted quantities are not causally deconfounded total effects. Independent reconstruction checked 384 candidate endpoints to 6.66×10^{-15}, rigid pair distances to 1.36×10^{-12} μm, all 252 failures, source/receiver exclusions and unique KP controls.

HCC geometry and complete sensitivity coverage. All 11 post-treatment sections and seven external sections were analysed with isotropic Visium coordinates $x = \mathrm{array\_col}/2$, $y = \sqrt{3}\,\mathrm{array\_row}/2$, normalized by their positive median nearest-neighbour spacing. Distances are in neighbour spacings, not micrometres. Original image pixels provide a sensitivity to the uncorrected row/column Euclidean metric. All source masks, score definitions, features, penalties and nested/locked validation rules were otherwise fixed (SM10). Analytic hex distances independently reproduce 110,073 post/external spot distances within 2.5×10^{-13}; 72 slopes were checked by covariance/variance formulas. Clinical results are reported in SM10; pixel-coordinate AUCs agree. Pixel/hex distance correlations of 0.999961–0.999996 do not make profiles identical: rounding and bin boundaries reassign about 14.5–21.7% of receivers.

The three coordinate branches plus the $q = 0.97/0.98/0.99$ and window-multiplier $0.75/1/1.25$ hex grid give 11 distinct specifications. A window-only change does not change the continuous slopes; identical clinical inputs were verified before reusing the corresponding fixed-q predictions. Atlas and reconstruction were actually rerun for every branch. Across the hex grid, internal SPF AUC is 0–0.5333 and combined AUC 0.6333–0.9000, below direct-only 0.9667; combined Brier is 0.16134–0.22809 versus direct-only 0.06638. External SPF AUC is 0.5–0.6667 and combined 0.5833–0.6667, below direct-only 0.8333. These exploratory specifications are not independent validation, and their best result was not selected as primary.

Coverage failures are explicit. At window multiplier 0.75, the first interval 0–0.75 neighbour spacings is structurally empty for non-source receivers (minimum lattice distance one); all 72 patient–programme curves lack that first bin. The

resulting atlas has only 46 non-HCC curves, so its clustering cannot establish HCC window robustness. At multiplier 1.25 with $q = 0.97/0.98$, four external patient–programme curves lack a remote bin, and only 58 complete atlas curves remain; $q = 0.99$ retains 62. Empty bins are neither zero-filled nor interpolated, and silhouettes on different support sets are not ranked as equivalent validations. Main hex geometry retains 62 curves in 48 radial-decaying and 14 locally suppressed groups, with silhouette 0.488826. The two-technology platform holdout has RMSE 0.354009 and ordinal energy 0.260084. These remain descriptive shapes rather than physical transport or clinical utility.

Fixed distance-window sensitivities in Clu and injury datasets. For Clu, near/far windows were scaled together by 0.75/1/1.25: 75 versus 300–600, 100 versus 400–800, and 125 versus 500–1,000 μm. The 100-μm source-graph threshold, GAM and same 99 source configurations were fixed. All-receiver fractions were 1.00/0.78/0.83, 0.37/0.91/0.71 and 0.82/0.99/0.82 across the three chips. Of 63 chip–window–endpoint tests, 62 were estimable; C02943C3 oligodendrocytes at multiplier 0.75 had only 28 near receivers, below 30, and remain NA. Neither seven-endpoint chip/window BH nor the full 63-test family yields $q < 0.05$ (full-family minimum 0.315). Windows are sensitivities, not new biological replications or proof that the spatial reference is calibrated.

For MI and skin, all 27 original H5AD sections and five expression-complete skin matrices were reread. Fixed proxy sources and score/library definitions were retained. MI coordinates were divided by their sample median nearest-neighbour distance and assigned the nominal Visium 100-μm centre spacing; the windows 150 versus 300–600, 200 versus 400–800 and 250 versus 500–1,000 on this scale correspond to 1.5 versus 3–6, 2 versus 4–8 and 2.5 versus 5–10 neighbour spacings. This is not an independently verified image-pixel calibration. Skin used its image-pixel/spot-diameter calibration, with the same numeric windows in micrometres. Twenty-one programme/window conditions and 369 section-level endpoints all retained near/far support. Exact MI patient-label fractions over 20 patients/four controls (4,845 allocations) ranged 0.190093–0.793395; skin section-label fractions over five sections/one control (five allocations) ranged 0.6–1. The two corrupted skin matrices were not silently restored. Independent analytic distances, direct contrasts and subset-sum label enumeration verified every result; primary-window inputs were unchanged. These results do not show absence of a field, persistence within one wound or extra animal/patient replication.

What the computations cannot supply. Additional independent animals, not more lesion/section resampling, are needed for population-level Perturb-map precision. More animals with adequate mSafe and target-guide capture, followed by independent validation, are needed for complete calibrated mouse-transfer folds; existing unknown guide identities cannot be recovered by declaring them negative. Orthogonal guide/editing and cell-resolved measurements are needed to distinguish true indirect responses from contamination and mixture/state alternatives. The actual rates of these errors are not identified by the simulation. A pre-defined, same-endpoint independent clinical cohort is needed to evaluate incremental HCC prediction; post-treatment proxy fields cannot be relabelled pretreatment causal predictors.

Some remaining needs concern records or annotation rather than necessarily new animals: the reference/spatial animal linkage, cross-section clone identities, and complete blinded tumour/tissue/niche boundaries should first be sought from original sample records, registration and available images. Existing coarse annotation cannot demonstrate exact matching positivity or eliminate unmeasured niche confounding. None of the new numerical checks proves source exchangeability, molecular transport, mediation or clinical deployability.

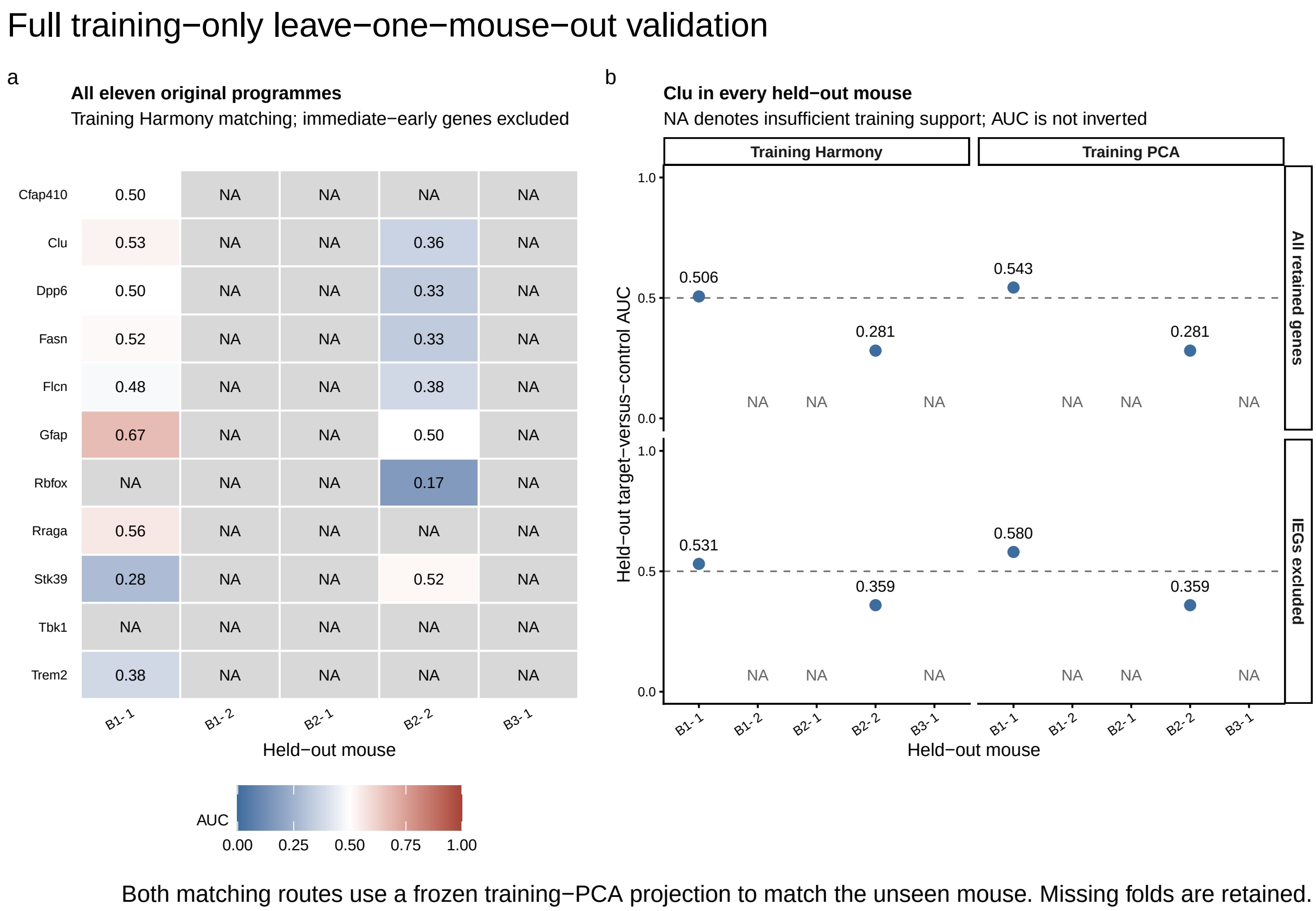

Supplementary Fig. 18: Strict training-only mouse-transfer diagnostic. a, The original 11 programmes under fold-specific training-Harmony matching, frozen training-PCA query matching and no-IEG scoring; all unavailable folds are displayed. **b**, Clu AUCs for primary/auxiliary matching routes and all/no-IEG genes. The 0.5 line is a descriptive discrimination reference. Low AUCs are not reversed; missing mSafe folds are not assigned zero. All weights and thresholds use training animals only. These small within-mouse diagnostics do not validate spatial propagation.

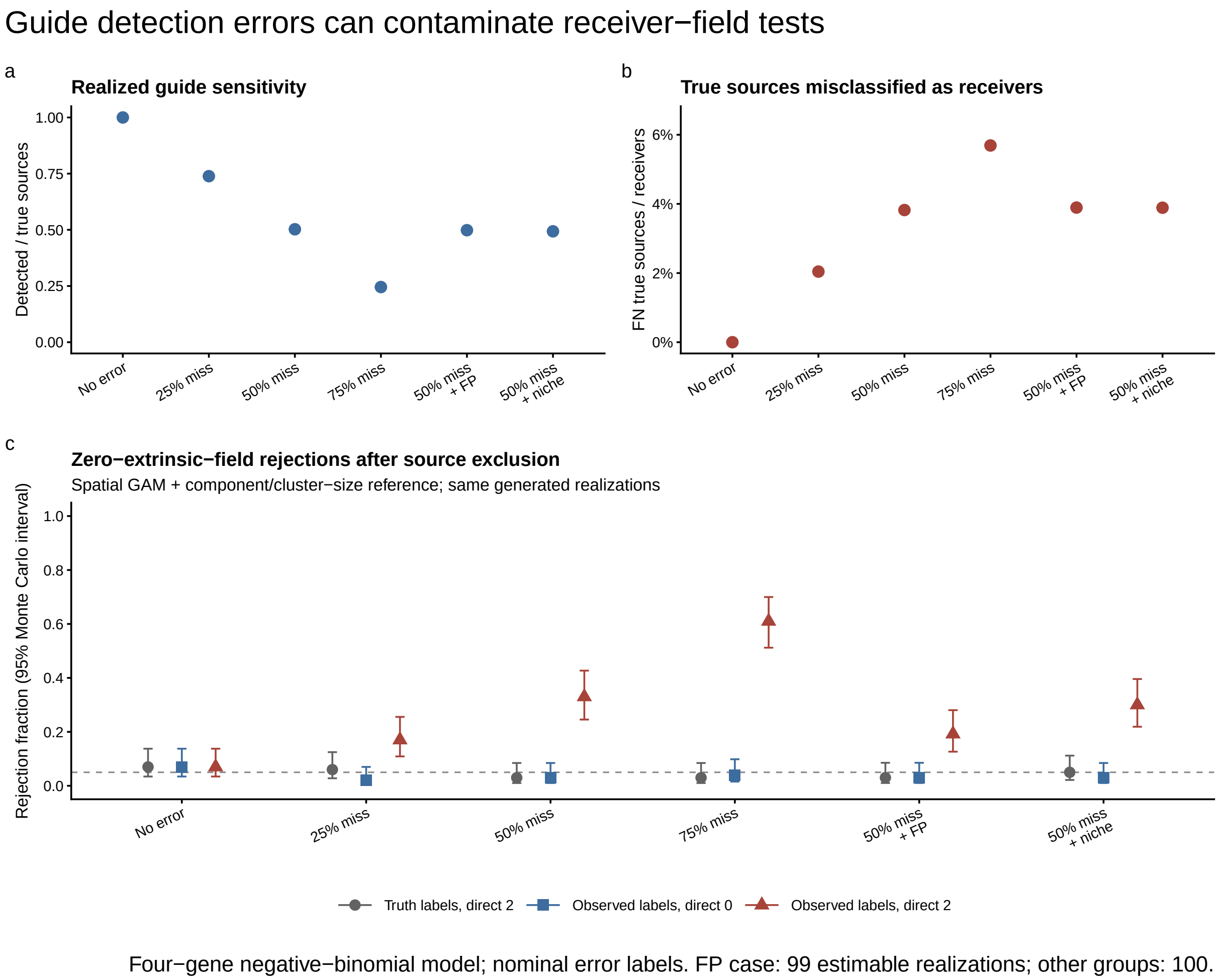

Supplementary Fig. 19: Repeated count-level guide error and receiver contamination. **a**, Mean realized true-source recall across six cases. **b**, Mean fraction of observed receivers that are missed true sources. **c**, Zero-extrinsic-field rejection under GAM and cluster placement with pseudo-source exclusion, showing oracle/direct-two, observed/direct-zero and observed/direct-two series. Missed true sources retain their direct effect in observed receivers. Wilson intervals summarize 100 generated realizations, with 99 estimable in false-positive-case groups; they are not animal confidence intervals or estimated real-assay error rates. The direct-zero control and unavailable outcomes are retained.

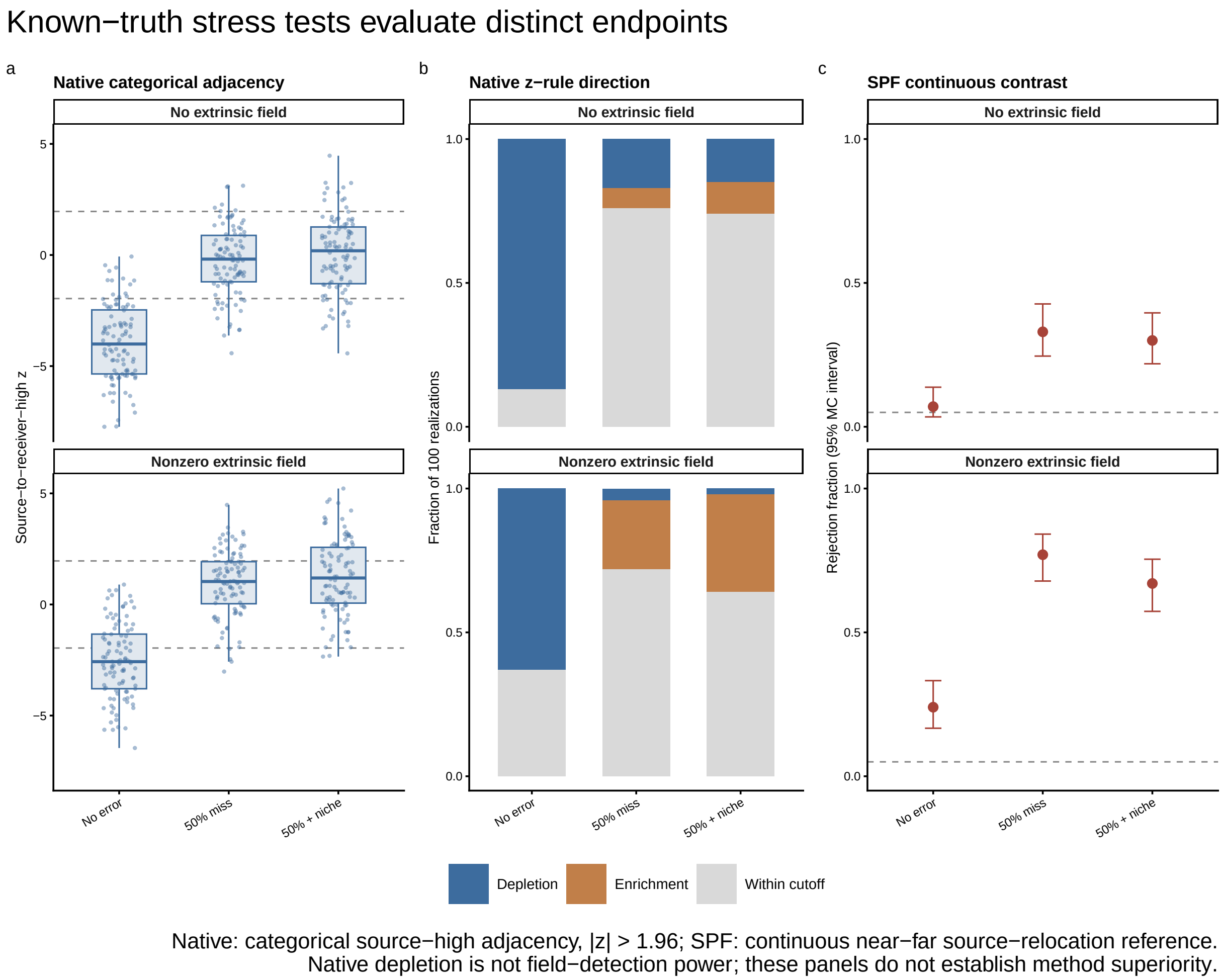

Supplementary Fig. 20: Native categorical adjacency on shared generated data. **a**, Signed source-to-high-receiver z distributions for 100 cases per error/amplitude group. **b**, Depletion/enrichment/within-threshold frequencies under the fixed $|z| > 1.96$ rule. **c**, Continuous receiver-field source-reference rejection on the shared generated cases, displayed as a different endpoint. Ideal-detection native exceedances at both amplitudes are depletion, not positive-field detection. Squidpy did not return empirical P values. The categorical-label null and graph endpoint differ from continuous receiver-field source placement; these frequencies are not a common-estimand software ranking or general type-I-error/power claim.

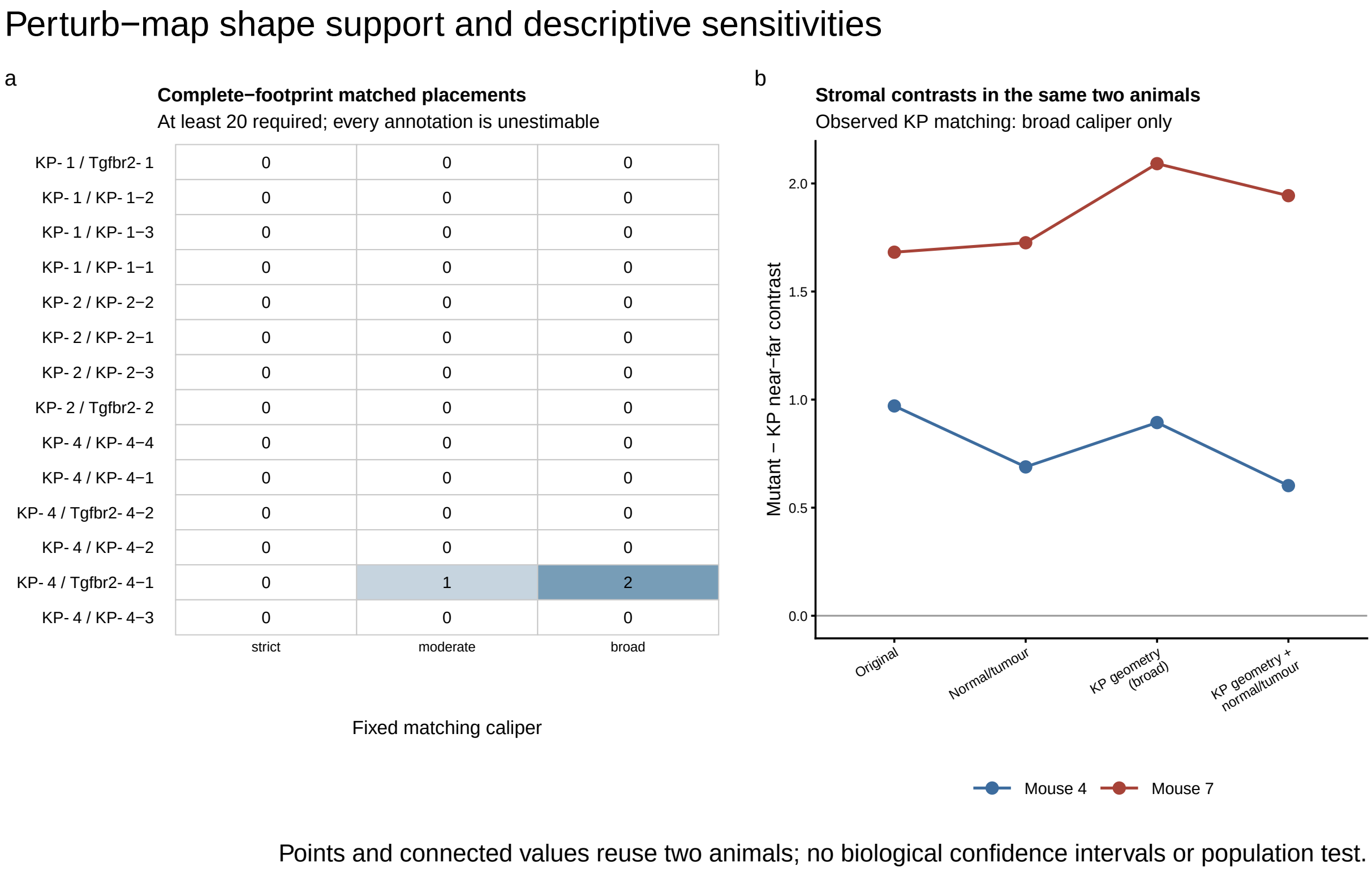

Supplementary Fig. 21: Executed shape/location support and descriptive two-animal controls. Complete rigid footprints are evaluated against the annotated capture mask, not a manually traced anatomical boundary. Legal-placement support fails the pre-defined tiers for the original animal estimand; unavailable contrasts remain unavailable. Broad real-KP geometric matching and coarse normal/tumour receiver standardization retain direction in the original two animals but neither preserve exact shape nor remove causal confounding. No fictitious animal confidence intervals or population P values are shown.

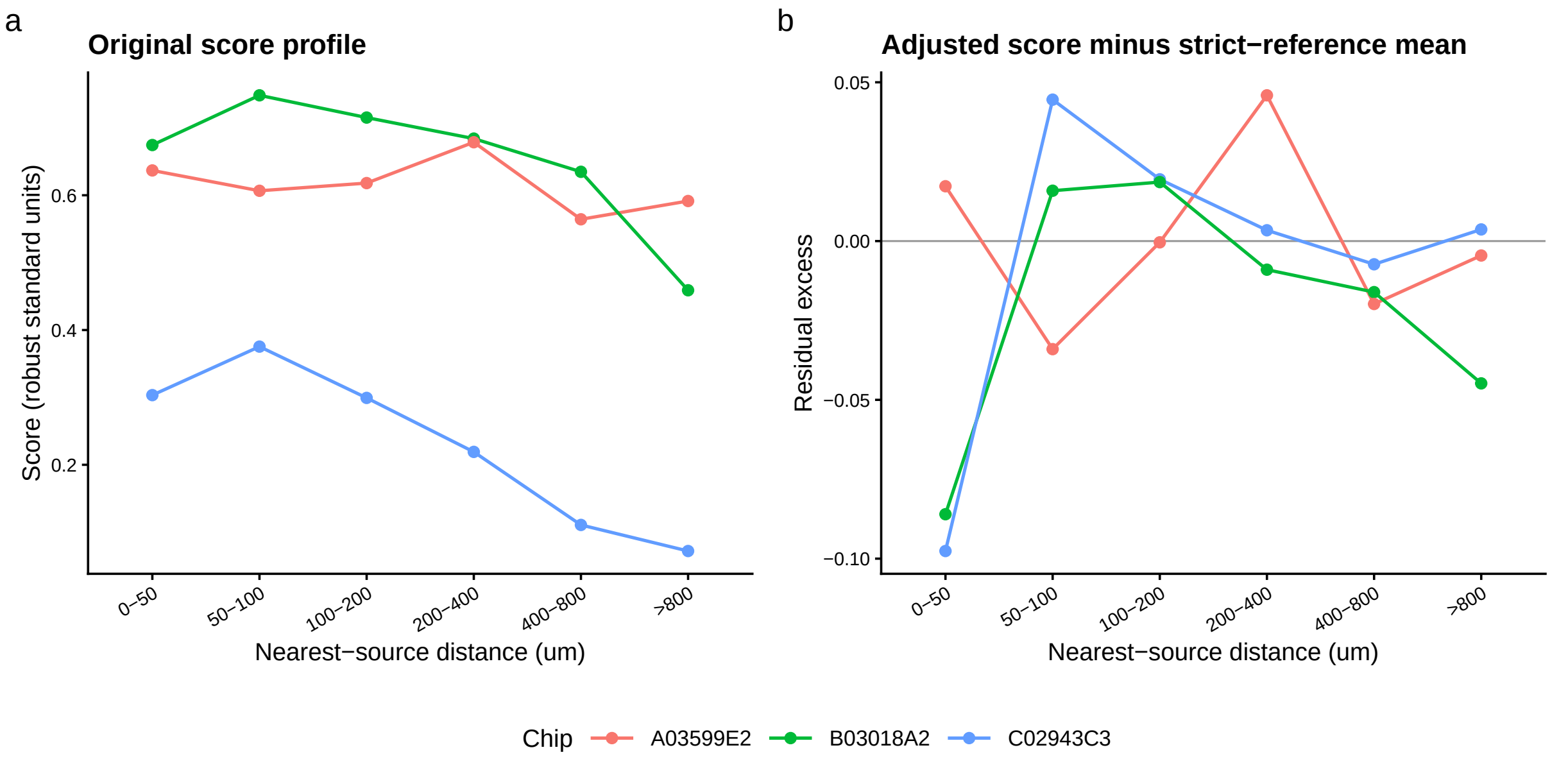

Supplementary Fig. 22: Original and strict Clu receiver profiles. a, Original no-immediate-early-gene score means by chip and nearest-source interval. b, GAM-adjusted means minus the cluster-null mean in the same distance intervals. Points/lines are descriptive chip profiles; cells do not provide independent biological confidence intervals. The two panels differ in both score adjustment and source reference and are not a single-control ablation. The full-chip background/reference decomposition is reported in SM14 and Supplementary Fig.24. Public repository input `data/endpoints.rds` retains `sps_profiles` and `clu_strict_profiles`.

SM14. Diagnostic operating conditions and additional control comparisons

Requirements for interpreting a spatial-reference comparison. SPF reports a receiver-score description until the assumptions needed for a stronger claim are justified. (1) The endpoint must be defined independently of the evaluated neighbourhood, supported by enough observations and validated for its intended biological interpretation. The Clu programme holdouts did not establish stable transfer. (2) Source ascertainment, receiver eligibility and competing exposures must support the claimed receiver population. Zero guide counts do not identify unedited cells; the guide-error simulation and mixed-guide geometry expose this limitation. (3) Reference support and an assignment/exchangeability law must justify the comparison. A source generator that retains some observed features is not automatically the true conditional source law; missing matched-placement support is a failure, not a null result. (4) Independent biological units and the design must match the claim. Two observed animals support descriptions of those animals, not population precision or causal mediation. Passing a computational gate is not sufficient evidence for the others.

For real Clu, observed guide-positive cells are excluded from the guide-undetected candidate pool, the initial receiver/GAM population is selected using observed guide calls, and the nominal group specification is estimated from the observed source graph. Regenerating nearest-neighbour groups and removing their selected positions from receivers does not reproduce an exchangeable full assignment procedure. Thus P_{ref} denotes a finite spatial-reference tail fraction. BH-transformed reference fractions are descriptive adjusted scores, not established false-discovery-rate control. Legacy code column names such as `permutation_p` and `fdr_within_chip` are retained for compatibility and do not add those guarantees. This restriction is separate from the mouse-paired edgeR gene tests and fixed-prediction clinical outcome-label references.

Conditional rank argument under a known assignment law. Let $\mathcal{F}$ contain fixed receiver locations, covariates, a source-independent eligibility rule and score transformation, and the resulting receiver scores under the null. Suppose the observed assignment and B simulated assignments are conditionally independent draws $S_0, \dots, S_B \sim Q(\cdot \mid \mathcal{F})$ from the *same known* law, with the same fixed specification and support rule. Set $T_j = T(S_j; \mathcal{F})$ and $c = (B+1)^{-1} \sum_{j=0}^{B} T_j$. The vector $A_j = |T_j - c|$ is exchangeable because the centre treats all indices symmetrically. Consequently

$$P_{\text{rank}} = \frac{\#\{j \in \{0, \dots, B\} : A_j \geq A_0\}}{B+1} \quad \text{satisfies} \quad \Pr(P_{\text{rank}} \leq \alpha \mid \mathcal{F}) \leq \alpha. \tag{19}$$

The weak inequality includes ties conservatively. If a common validity criterion applied identically to every assignment excludes assignments, the same argument applies conditional on validity and the number of valid reference draws, provided the observed assignment is also valid and the score law remains independent of assignment. This is the standard exchangeable-rank principle, not a new randomization theorem. It does not apply when Q is unknown, specifications are conditioned on the observed assignment without reproducing their correct conditional law, receivers are assignment-selected, or direct source effects enter them. Those are precisely unresolved features of the real assays here. More configurations or a symmetric centre cannot substitute for these premises.

Independent-bank comparison of the same continuous endpoint. A separate protocol was fixed before its outcomes, using six generating settings from the existing family: baseline, disconnected tissue, singleton sources, source–niche restriction, receiver composition and 50% score masking. Each has 100 base repetitions, amplitudes zero/one, GAM adjustment and 199 fresh source configurations *per repetition and generator*. The source-count, nominal-group, niche and score definitions are fixed in the protocol. True sources and the design-matched reference use the identical fixed-specification anchor–niche law Q; cluster-only placement supplies a different conditional law when anchor–niche restrictions add information. In the disconnected setting the coarse niche equals the tissue component, so the two laws coincide; their independently drawn finite banks can still give different tail fractions. Receiver and candidate-source populations are separate in this model, so the fixed-receiver premise is explicit. L'Ecuyer streams separately generate sources, background/masking and each reference bank. Geometry and stream realizations differ from SM12: this is a new conditional calibration study, not a single-factor causal comparison of shared versus independent banks.

All six settings, both references and both amplitudes are retained: 600 base repetitions, 2,400 test records and 24 summaries. All endpoints are estimable and every comparison has 199 finite configurations. At zero amplitude, the design-matched law rejects 5, 6, 7, 3, 1 and 2 of 100 repetitions; cluster-only placement rejects 5, 8, 8, 6, 1 and 1. All

Supplementary Table 16
Fresh-bank known-assignment comparison. Entries are rejections out of 100 base repetitions at the stated amplitude and $P_{\text{rank}} \leq 0.05$. The matched law is known only in this generated model; the table is not a calibration certificate for real observational source calls.

Setting	Matched Q, zero	Cluster only, zero	Matched Q, one	Cluster only, one
Baseline	5	5	72	74
Disconnected	6	8	69	68
Singleton	7	8	88	89
Source–niche	3	6	69	64
Composition	1	1	83	83
Score masking	2	1	36	35

twelve zero-amplitude Wilson lower limits are at most 0.05. These finite frequencies are compatible with the stated model premises; finite simulations do not establish universal calibration. At amplitude one, design-matched detection ranges from 36/100 under score masking to 88/100 with singletons. The table gives every result; the full intervals and individual records accompany the code. The 1,249 independent read-back rank, effect, Wilson, distance, random-stream and replay checks all passed. Inference is conditional on this model's source-assignment and score-masking assumptions; score masking does not model guide misclassification.

Real-map control audit. Five background choices were crossed with two source banks in each of the three Clu chips. The nested path is raw score, identity-only linear adjustment, identity plus a library-size smooth, and the full spatial GAM; a global-linear identity/library/x/y model is a separate sensitivity branch. Within each source generator the same 99 configurations are reused across all background choices, with the same observed receivers, 100 versus 400–800 μm windows and minimum 30 near/far observations. Each pseudo-source is excluded from its own receiver statistic. Cluster configurations use the existing bank; count configurations preserve source numbers by component with a new fixed seed. Count results follow the uniform source-count rule, which differs from the exploratory screen described above.

For the cluster bank, the raw/identity/library/full-GAM P_{ref} sequences are 0.01/0.01/0.32/0.78 in C02943C3, 0.17/0.31/0.95/0.91 in B03018A2 and 0.06/0.13/0.21/0.99 in A03599E2. Full-GAM reference-adjusted effects reproduce 0.0078115, −0.0024411 and 0.0010659. The raw count-bank fractions are 0.01/0.82/0.09; cluster placement is therefore not uniformly more conservative. The 30 endpoints and 2,970 simulated statistics show dependence on background adjustment as well as source reference. They do not allocate causal shares to composition, library effects or spatial confounding, which can also align with genuine biology. All 3,325 primary and 321 independent checks passed, with independent maximum error 5.23×10^{-14}. The separately executed portable full-model route restored all three primary endpoints and mixed-exposure tables.

Detected mixed-guide geometry. Other-source distance is to the nearest non-Clu detected single-guide cell within the same tissue component; mSafe is included as a detected control and is not assumed to cause a biological effect. Multiple-guide cells are evaluated separately. Among guide-undetected receivers in the Clu near window, 84.6%, 75.1% and 62.6% are within 100 μm of another detected single-guide source. Corresponding far-window fractions are 4.8%, 28.6% and 8.7%. The Clu sources themselves have such neighbours in 26/29, 366/419 and 40/60 cases. Single- and multiple-guide neighbourhoods can overlap; their fractions are not added. The high frequency of another guide being nearer than Clu in the far ring is partly induced by conditioning on Clu distance, not evidence for a stronger other-guide effect. These are geometric co-exposure diagnostics, not observed molecular contamination or a multi-guide causal regression. Excluding detected other-guide cells from receivers therefore does not remove potential neighbourhood exposure. No unique Clu attribution, no clean-unexposed subgroup and no resolved guide-interaction effect is established.

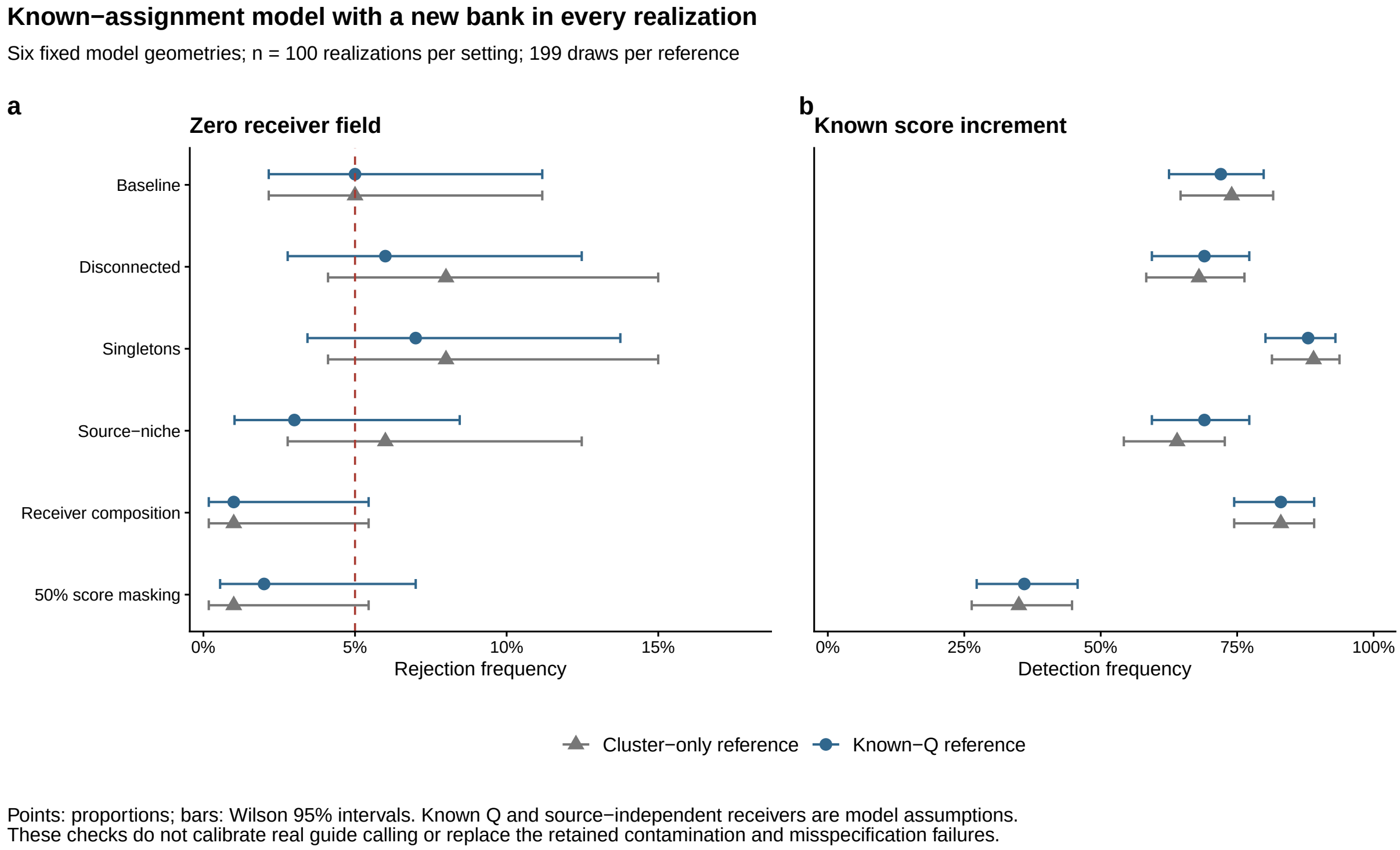

Supplementary Fig. 23: Independent-bank reference comparison under a known assignment model. Six fixed settings each have 100 independent base repetitions and 199 newly sampled configurations per repetition/reference. Zero-amplitude rejection and amplitude-one detection compare the identical continuous near–far/GAM endpoint under the true generating anchor–niche law and cluster-only placement. Wilson bars describe Monte Carlo uncertainty; different methods/amplitudes reuse the same base repetitions. The benchmark assumes a fixed receiver set and known Q; inference is conditional on these generated-data premises and does not calibrate guide-selected observational sources.

a Observed minus source−reference mean

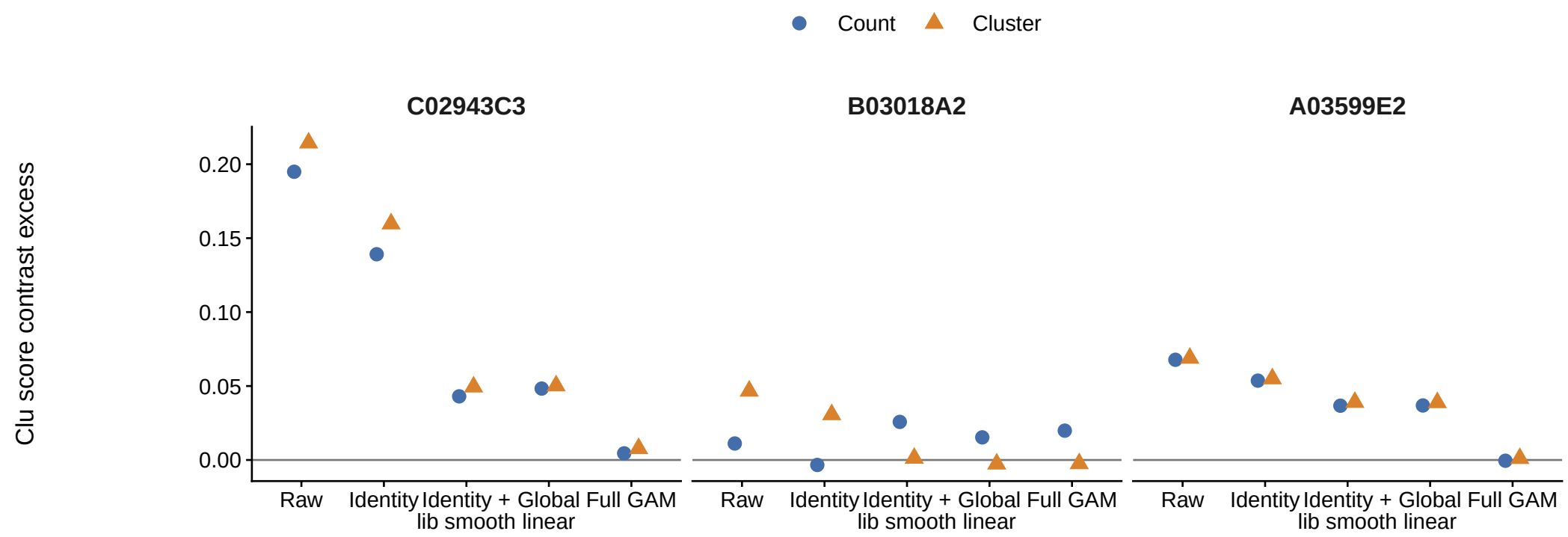

b Conditional common−centre tail fractions; 99 configurations each

	Raw	Identity	Identity + lib smooth	Global linear	Full GAM
C02943C3 | Count	0.01	0.02	0.24	0.20	0.86
C02943C3 | Cluster	0.01	0.01	0.32	0.29	0.78
B03018A2 | Count	0.82	0.93	0.52	0.71	0.60
B03018A2 | Cluster	0.17	0.31	0.95	0.92	0.91
A03599E2 | Count	0.09	0.10	0.27	0.29	1.00
A03599E2 | Cluster	0.06	0.13	0.21	0.18	0.99

c Receiver proximity to other detected guide sources

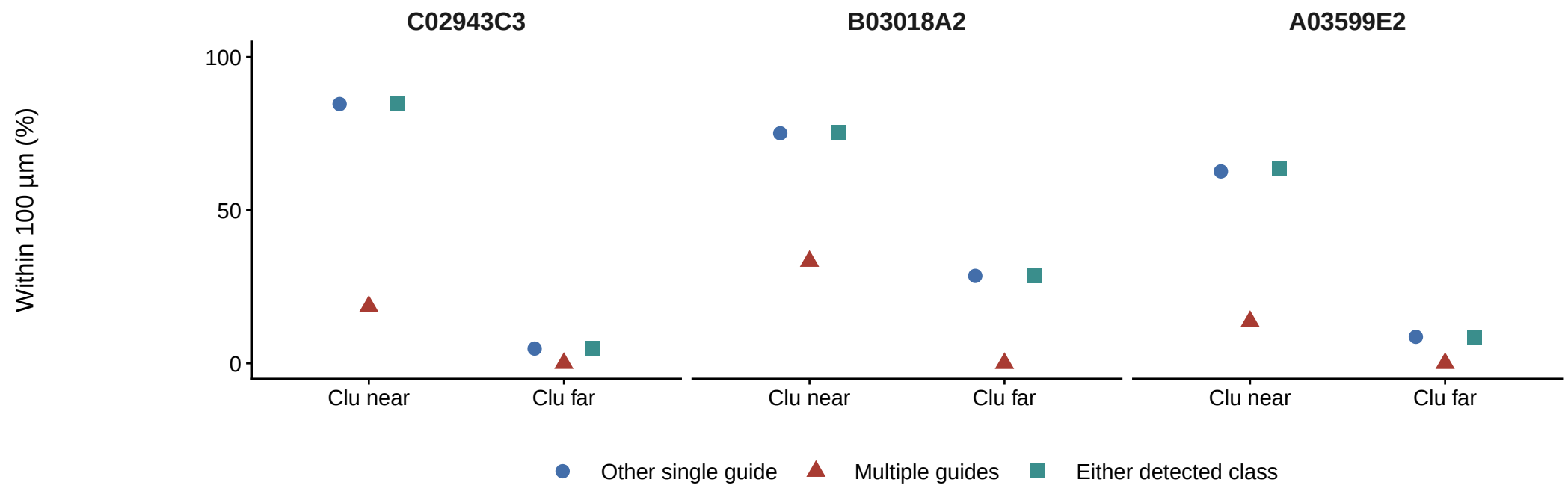

Supplementary Fig. 24: Actual Clu control paths and detected mixed-guide geometry. Thirty fixed chip/background/reference combinations retain the same source banks across adjustments. The identity/library/full-GAM path is nested; global-linear adjustment is a separate branch. Reference fractions are conditional diagnostics, and clustering is not uniformly more conservative. Mixed-guide coverage reports detected spatial co-exposure among Clu near/far receivers, with multiple-guide cells separate; mSafe belongs to the detected other-single-guide category. Geometry does not establish another guide's molecular effect or remove confounding. All analyses reuse three chips spanning four mice, including one pooled chip.

Supplementary Table 17
Fixed-window Perturb-map score diagnostics. All values are descriptive mutant-minus-KP near–far contrasts in the same two animals; columns are not independent study replications. Each changed score is standardized separately.

Definition	Mouse 4	Mouse 7	Equal-animal mean
Original primary	0.9707	1.6820	1.3263
Target without matrix genes	0.7493	1.8026	1.2759
Primary without matrix genes	0.5991	0.3462	0.4726
Omit Serpine1	0.9457	1.6539	1.2998
Omit Col1a1	0.9322	1.4592	1.1957
Omit Col1a2	0.9452	1.4098	1.1775
Omit Col3a1	0.9934	1.5255	1.2594
Omit Fn1	0.8921	1.6520	1.2721
Omit Tnc	0.9345	1.6051	1.2698
Omit Timp1	0.9543	1.5528	1.2536
Omit Tgfb1	0.9588	1.6406	1.2997
Omit Spp1	1.0304	2.1357	1.5830
Mean of gene-wise logs	1.0522	1.7658	1.4090
Mean of gene-wise standardized values	1.1750	1.7536	1.4643

SM15. Additional score, exposure and prediction diagnostics

Gene influence and programme overlap in Perturb-map. These exploratory diagnostics retain the primary 200-µm near and 400–800-µm far windows, original receiver/reference masks, whole-library denominators and section-to-animal aggregation. The original nine measured stromal genes are unchanged. Each leave-one-gene-out score is recomputed from counts using the original log-of-mean formula and its own reference-receiver median/MAD scaling. All nine omissions are retained; their equal-animal contrasts range from 1.1775 to 1.5830, with positive contrasts in both animals. This excludes a sign change caused by removing any one measured gene under this definition, but does not establish independent replication or pathway specificity. Individual Serpine1 and Spp1 scores have negative contrasts in mouse 7, so concordance of the aggregate is not concordance of every gene. Averaging gene-wise logged expression and averaging gene-wise standardized expression are separate sensitivity scores; they do not replace the primary endpoint.

The measured primary, target and matrix programmes contain nine, ten and five genes. All five matrix genes occur in both other sets; primary and target share seven genes (Jaccard $7/12$). Removing the five matrix genes leaves four primary genes and five target genes. Their recomputed equal-animal contrasts remain positive, 0.4726 and 1.2759, respectively (Table 17). These are within-animal descriptive checks of gene overlap, not independent receiver pathways or a mediation analysis. Cell mixture, related transcriptional states and overlapping receiver neighbourhoods remain possible explanations. The repository's `diagnostics` command writes normalized-count gene shares to `perturbmap/primary_gene_count_share.tsv`; these are not interpreted as shares of the nonlinear score effect or variance.

Excluding detected alternative-guide neighbourhoods. Starting with the fixed Clu score, all guide-undetected receivers within 100 µm of any other detected single-guide cell, including mSafe, or a multiple-guide cell were removed. Distances respect the same tissue components. This exposure mask is fixed for observed and reference configurations; each reference additionally removes its pseudo-source positions. The original GAM, fitted on all guide-undetected receivers, and the original 99 cluster configurations are retained to isolate receiver-population sensitivity. Pseudo-source candidates remain all original guide-undetected cells, including positions excluded from this restricted receiver population. It is not a refit on the restricted population, an interaction model or an exchangeable reassignment procedure. Raw scores and these same frozen-GAM residuals are both reported for every chip.

The restricted near windows retain 189, 3,466 and 870 receivers, versus 1,261, 14,085 and 2,382 originally. Frozen-GAM reference excesses become 0.03995, 0.05459 and 0.02181, with $P_{\text{ref}} = 0.35$, 0.05 and 0.54. The pooled middle chip is therefore at the nominal 0.05 reference boundary in this changed population, rather than uniformly insensitive

Supplementary Table 18
Clu-score receiver and source-position diagnostics. Windows are 100 versus 400–800 μm. Every endpoint has 99 finite reference draws. Excluding detected alternative-guide neighbourhoods changes the receiver population; mSafe uses a different source set. Neither comparison identifies biological truth.

Chip	Population/source	Sources	Near	Far	Raw P_{ref}	GAM excess	GAM P_{ref}
C02943C3	Original Clu	29	1261	8880	0.01	0.0078	0.78
C02943C3	Restricted Clu	29	189	8451	0.03	0.0399	0.35
C02943C3	mSafe positions	7	368	7781	0.01	0.0510	0.39
B03018A2	Original Clu	419	14085	34256	0.17	-0.0024	0.91
B03018A2	Restricted Clu	419	3466	24472	0.25	0.0546	0.05
B03018A2	mSafe positions	96	3922	36472	0.09	0.0129	0.67
A03599E2	Original Clu	60	2382	16473	0.06	0.0011	0.99
A03599E2	Restricted Clu	60	870	15042	0.05	0.0218	0.54
A03599E2	mSafe positions	9	447	11232	0.36	-0.1223	0.07

to receiver selection. These descriptive fractions have neither established type-I-error control nor biological-replicate uncertainty. Residual hidden-guide exposure and long-range or overlapping responses are not excluded by a 100-μm mask. No clean-unexposed subgroup or uniquely attributable Clu effect is established.

An mSafe-placement diagnostic uses the same Clu receiver score, original receiver population and background, but the observed mSafe positions (7, 96 and 9 source cells) and a newly seeded 99-draw cluster-size reference. It is a falsification check of score/placement specificity, not a newly learned mSafe response programme. Raw fractions are 0.01, 0.09 and 0.36; frozen-GAM fractions are 0.39, 0.67 and 0.07. Location selection, other-guide exposure and sparse sources prevent treating these three observations as an empirical null calibration or estimating an assay false-positive rate. The public `Rscript run.R diagnostics OUTPUT` command writes all 18 endpoints and 1,782 reference statistics to `clu/clu_exposure_result.tsv` and `clu/clu_exposure_refs.tsv`, respectively, within the selected output directory.

Training-compatible clinical programme membership. The paired assay contains 18,085 feature symbols and lacks CD8B, LDHA and BNIP3. All seven external assays include these symbols, so independently intersecting each programme with each assay had changed cytotoxic membership from seven to eight and hypoxia membership from five to seven. The corrected primary analysis freezes the same 35 measured programme genes in both cohorts (Table 20); apoptosis, interferon and fibrosis membership are unchanged. Training features remain numerically unchanged. External features, all eleven coordinate/threshold/window branches, locked predictions, ordinal atlases and reconstruction summaries were recomputed. At source quantile 0.99, the combined external AUC changes from 0.5833 to 0.6667 at each of the three window scales. Primary external SPF/combined AUCs remain 0.6667/0.5833; their Brier scores change to 0.264666/0.571410. The primary 62-curve atlas retains two groups of 48/14 curves, with silhouette 0.488826 and platform RMSE/energy 0.354009/0.260084.

Membership harmonization does not harmonize measurement universes, tissues or treatments. All seven external direct-intensity features lie below the training minimum; interferon, cytotoxic, fibrosis and hypoxia slopes lie outside their training ranges in 6/7, 6/7, 4/7 and 0/7 specimens. A separate fixed sensitivity normalizes both assays using the 17,779 shared symbols unique in every assay, excluding six shared ambiguous symbols; none of the 35 programme members is affected. All source calls, features and models are then recomputed at the primary hex geometry and threshold. External direct/SPF/combined AUCs become 0.6667/0.7500/0.5833; SPF Brier is 0.2611, versus 0.2586 for fixed training prevalence. The number of direct-intensity features outside the training range falls from seven to three. This sensitivity exposes part of the measurement dependence without assuming batch calibration or selecting its improved SPF ranking as primary.

Patient 85567 has a liver-primary pretreatment sample (1,155 spots) and a lymph-node-metastasis post-treatment sample (3,089 spots), according to the supplied sample metadata. Its paired difference combines treatment, anatomy and sampling. Excluding this pair leaves ten patients with liver samples at both time points (four responders/six non-responders); this metadata-defined sensitivity refits all training/scaling/penalty choices. Internal direct/SPF/combined LOPO AUCs are 0.9583/0/0.6250, and corresponding same-model pairwise AUCs are 0.9583/0.6250/0.8750. Locked external SPF and combined AUC/Brier become 0.5833/0.3471 and 0.6667/0.5451; direct AUC remains 0.8333. The training-prevalence baseline for this branch is 0.4, with external Brier 0.2743. No branch establishes consistent incremental clinical value.

Supplementary Table 19
Same-model held-out-pair ranking versus pooled LOPO predictions. LOPO Brier uses one prediction per patient. Pairwise AUC averages within-fit ranks and has no pairwise Brier or independent-pair interval.

Training cohort	Model	LOPO AUC	LOPO Brier	Pairwise AUC	Pairs
All 11 pairs	Direct + SPF	0.7667	0.2062	0.8667	30
All 11 pairs	Direct	0.9667	0.0664	0.9667	30
All 11 pairs	Pretreatment	1.0000	0.0116	1.0000	30
All 11 pairs	SPF	0.0000	0.3652	0.6000	30
All 11 pairs	Intercept only	0.0000	0.3000	0.5000	30
10 liver pairs	Direct + SPF	0.6250	0.2037	0.8750	24
10 liver pairs	Direct	0.9583	0.0885	0.9583	24
10 liver pairs	Pretreatment	1.0000	0.0364	1.0000	24
10 liver pairs	SPF	0.0000	0.3607	0.6250	24
10 liver pairs	Intercept only	0.0000	0.2963	0.5000	24

Supplementary Table 20
HCC programme membership and cross-cohort support. Counts use the same feature definitions, prior to the separately labelled common-library sensitivity. Range failures are descriptive feature support checks, not tests of population distribution equality.

Programme	Paired training	Unharmonized external	Harmonized external
Apoptosis	8	8	8
Interferon	7	7	7
Cytotoxic	7	8	7
Fibrosis	8	8	8
Hypoxia	5	7	5

External feature	Outside training range	External specimens
Direct apoptosis intensity	7	7
Interferon slope	6	7
Cytotoxic slope	6	7
Fibrosis slope	4	7
Hypoxia slope	0	7

Pairwise ranking and probability baselines. The primary pooled nested-LOPO AUC compares predictions produced by different training fits. With five responders and six non-responders, withholding a responder leaves a training prevalence of 4/10, whereas withholding a non-responder leaves 5/10. This alone gives intercept-only pooled AUC zero, despite no predictor inversion. A nested leave-pair-out diagnostic withholds one responder and one non-responder together; feature scaling and the same five-value ridge grid are refit within the remaining nine patients, including inner LOPO tuning. The held-out pair is ranked under its single shared model. The thirty pair rankings are averaged; patients recur across pairs, so no independent-pair confidence interval or pairwise Brier score is computed. SPF pairwise AUC is 0.6000, combined 0.8667, direct 0.9667 and pretreatment 1.0000; intercept is 0.5000 (Table 19). This resolves the interpretation of the extreme pooled ranking without replacing its primary probability errors or proving predictive utility.

The external probability baseline always uses the training prevalence, 5/11, without external-label tuning. Its external Brier error is 0.258560, compared with corrected SPF 0.264666 and combined 0.571410. Thus SPF's lower error than the direct model is not evidence that it improves over a no-feature probability forecast. The seven external outcomes and heterogeneous response definitions remain inadequate for a clinical calibration claim.

Source/receiver separation in the reusable interface. The coordinate-only new-data interface requires unique component/x/y measurement addresses within each input and disjoint observed-source and receiver addresses. Ambiguous duplicate addresses or source–receiver overlap produce an explicit error before fitting; observations are not silently removed. Distinct biological units at an identical address require a resolved input design before using this interface. In a deterministic 841-position fixture, only nine source positions had an autonomous increment and receivers had no source-associated field. Including those nine sources among the receivers yielded 11 of 12 reference fractions equal to 0.01; excluding them yielded fractions from 0.38 to 0.64. This controlled fixture quantifies the effect of source–receiver overlap on the interface statistic. Guard regressions preserve all twelve values for the valid disjoint input. The published Clu inputs already have disjoint sources/receivers; their primary estimates are unchanged. Invalid observed statistics or entirely unavailable references remain NA in the strict Clu implementation, including the directional statistic.

Reproduction and interpretation. The public repository supplies raw-count score diagnostics, fixed-bank exposure checks, clinical diagnostics and the corrected raw HCC reader. `Rscript run.R diagnostics OUTPUT` regenerates the compact diagnostics from existing inputs. The command writes score-definition, exposure, prediction and validation tables to the selected output directory. Corrected primary HCC features and native distance profiles are retained in `data/endpoints.rds`; `optional/external_features.R` regenerates these HCC feature/profile inputs from the original public matrices. The downstream fits and ordinal reconstruction use `R/clinical.R`, `R/atlas.R` and `R/profiles.R`. The HCC sensitivity objects in `data/computational_checks.rds` are completed-output snapshots (SM13). These checks add neither animals nor verified editing states. Negative or positive sensitivity results retain the score, source-assignment and replication limits stated in SM14.
